# Evolution of genome fragility enables microbial division of labor

**DOI:** 10.15252/msb.202211353

**Published:** 2023-02-02

**Authors:** Enrico Sandro Colizzi, Bram van Dijk, Roeland M H Merks, Daniel E Rozen, Renske M A Vroomans

**Affiliations:** ^1^ Mathematical Institute Leiden University Leiden The Netherlands; ^2^ Origins Center Leiden The Netherlands; ^3^ Sainsbury Laboratory Cambridge University Cambridge UK; ^4^ Department of Microbial Population Biology Max Planck Institute for Evolutionary Biology Plön Germany; ^5^ Institute of Biology Leiden University Leiden The Netherlands; ^6^ Informatic Institute University of Amsterdam Amsterdam The Netherlands

**Keywords:** division of labor, evolution, evolvability, multiscale modeling, *Streptomyces*, Computational Biology, Evolution & Ecology, Microbiology, Virology & Host Pathogen Interaction

## Abstract

Division of labor can evolve when social groups benefit from the functional specialization of its members. Recently, a novel means of coordinating the division of labor was found in the antibiotic‐producing bacterium *Streptomyces coelicolor*, where specialized cells are generated through large‐scale genomic re‐organization. We investigate how the evolution of a genome architecture enables such mutation‐driven division of labor, using a multiscale computational model of bacterial evolution. In this model, bacterial behavior—antibiotic production or replication—is determined by the structure and composition of their genome, which encodes antibiotics, growth‐promoting genes, and fragile genomic loci that can induce chromosomal deletions. We find that a genomic organization evolves, which partitions growth‐promoting genes and antibiotic‐coding genes into distinct parts of the genome, separated by fragile genomic loci. Mutations caused by these fragile sites mostly delete growth‐promoting genes, generating sterile, and antibiotic‐producing mutants from weakly‐producing progenitors, in agreement with experimental observations. This division of labor enhances the competition between colonies by promoting antibiotic diversity. These results show that genomic organization can co‐evolve with genomic instabilities to enable reproductive division of labor.

## Graphical abstract

A computational model of the multicellular bacteria *Streptomyces* reveals that if antibiotic production trades off with replication, genome architecture can evolve to support a mutation‐driven division of labor.

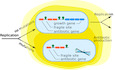

## Introduction

Multicellular organisms provide a clear example of a reproductive division of labor, where the germline produces gametes that generate offspring, while the somatic tissues carry out all the other functions that improve survival. Similar divisions of labor are found in colonies of social insects, where one or few individuals are responsible for all of the reproduction, whereas the other individuals perform tasks that mirror those of somatic cells in multicellular organisms. Recently, several striking examples of reproductive and other divisions of labor have been described in the microbial world (Ratcliff *et al*, 2012; Claessen *et al*, 2014; Gestel *et al*, 2015; Dragoš *et al*, 2018; Giri *et al*, 2019); it has been proposed that reproductive division of labor existed already before the Origin of Life, among prebiotic replicators (Takeuchi *et al*, 2011; Boza *et al*, 2014; Colizzi & Hogeweg, 2014). Thus, such divisions of labor are ubiquitous, and the mechanisms driving them may be diverse.

In a multicellular reproductive division of labor, somatic cells typically carry the same genetic information as the germline. Cell specialization is brought about by a combination of gene regulation and epigenetics, ensuring that only a small subset of the genome is expressed. Recently, an alternative route to reproductive division of labor has been observed in the antibiotic‐producing bacterium *Streptomyces coelicolor*—which generates somatic cells through mutations rather than gene regulation. Here, we propose that the genome of *S. coelicolor* has become structured over evolutionary time such that mutations occur frequently to yield differentiated cells, giving rise to a reproducible division of labor.

Streptomycetes are multicellular bacteria that grow from haploid spores, first producing a vegetative mycelium, and then differentiating into aerial hyphae that produce environmentally resistant spores. During differentiation into aerial hyphae, colonies produce a diverse repertoire of secondary metabolites (McCormick & Flärdh, 2012), including antibiotics that are used to regulate competitive interactions between strains (Abrudan *et al*, 2015). The genus is estimated to produce about 10^5^ different antibiotics (Watve *et al*, 2001), with each species carrying up to 30 biosynthetic gene clusters for specialized metabolites (Genilloud, 2014), some of which encode antibiotics and/or resistance to them (Mak *et al*, 2014). Recent results with *S. coelicolor* suggest that antibiotic production and spore formation are carried out by distinct cell types. Antibiotic synthesis and secretion are metabolically expensive tasks that trade‐off with replication (Zhang *et al*, 2020b); accordingly, colony fitness is expected to be higher when these tasks are partitioned into separate cells (Ispolatov *et al*, 2011). The antibiotic‐hyperproducing subset of cells in *S. coelicolor* colonies arises due to massive and irreversible deletions at the left and right arms of the *Streptomyces* linear chromosome (Zhang *et al*, 2020b). Cells with larger deletions produce more antibiotics but also produce significantly fewer spores, a deficit that effectively ensures their elimination during each replicative cycle (preprint: Zhang *et al*, 2020a). These antibiotic‐hyperproducing cells are instead repeatedly re‐generated independently in each colony following spore germination. This process gives rise to heterogeneous colonies containing a diversity of mutants with different chromosome sizes that produce different combinations of antibiotics, as well as a larger fraction of cells specialized for spore production (Zhang *et al*, 2020b).

The irreversible mutational mechanism used to generate division of labor in *S. coelicolor* may be widespread in the genus, which is well known for its genome instability (Volff & Altenbuchner, 1998; Chen *et al*, 2002; Hopwood, 2006). Many bacterial genomes are organized such that some regions of the chromosome show higher genetic variation than others, for example through the targeted insertion of mobile genetic elements, that can also carry functional genes (Oliveira *et al*, 2017), or through deletion at specific chromosomal fragile sites (Mei *et al*, 2021). In *Streptomyces*, these variable chromosomal regions are located towards the telomeres of their linear chromosome and are known to evolve rapidly through DNA amplification, insertion, deletion, and recombination (Hoff *et al*, 2018; Tidjani *et al*, 2019, 2020). In particular, megabase‐long deletions occur frequently in the *Streptomyces* chromosome (Birch *et al*, 1990; Leblond & Decaris, 1994; Volff & Altenbuchner, 1998; preprint: Zhang *et al*, 2020a; Zhang *et al*, 2020b). These mutations are generated through homologous and nonhomologous recombination (possibly of sister chromatids; Fischer *et al*, 1998) of repeated genomic elements, typically transposons, which are unusually abundant towards the telomeres of the *Streptomyces* chromosome (Volff & Altenbuchner, 1998; Chen *et al*, 2002). But how does this instability reliably generate antibiotic‐producing cells? Here, we hypothesize that chromosomal gene order has evolved such that some functional groups of genes have localized at the telomeric ends of the chromosome, making them more susceptible to deletion due to genome instability. By this argument, genome instability becomes adaptive within the context of this genome organization, because it facilitates the generation of sterile antibiotic‐producing mutants from replicating cells. We show that a genome architecture capable of generating a mutation‐driven division of labor evolves in a computational model of antibiotic‐producing bacterial colonies.

## Results

### Model overview

We formulated a computational model based on a simplified description of the multicellular life cycle and ecology of *Streptomyces*. We focus on the vegetative growth stage, especially during the developmental transition to sporulation (Claessen *et al*, 2014). In this phase, colonies grow, interact and compete with one another for space and resources (Hopwood, 2006; Vetsigian *et al*, 2011; Abrudan *et al*, 2015). This growth‐dominated phase is followed by a phase dominated by investment in secondary metabolism (Bibb, 2005; McCormick & Flärdh, 2012; Liu *et al*, 2013; Schlatter & Kinkel, 2015). Secreted antibiotics diffuse around the producing colony, protecting it from competing strains and allowing it to claim the space into which it can grow (Westhoff *et al*, 2020). Finally, sporulation is induced when colonies experience resource limitations.

**Figure 1:**
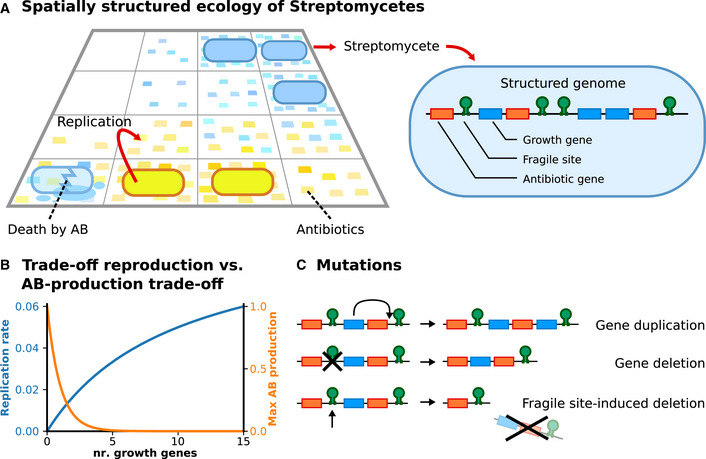
The model: a population of virtual *Streptomyces* evolving through many cycles of colonial growth of duration *τ*_*s*_ = 2,500 time steps Bacteria replicate locally on a two‐dimensional surface. They produce antibiotics, which are placed in their vicinity, to which other bacteria may be susceptible. Each bacterium contains a genome—a linear sequence of genes and genetic elements. We consider two gene types—growth‐promoting genes and antibiotic genes—as well as fragile sites.The metabolic strategy of a bacterium is determined by its genome: A larger number of growth‐promoting genes translate to higher growth and lower antibiotic production. Default parameter values are: $\alpha_{g}=0.1$, $h_{g}=10$, $\beta_{g}=1$, unless explicitly stated otherwise.Bacterial genomes mutate during replication: Single gene duplications and deletions occur at random locations on the genome with probability $\mu_{d}=10^{-3}$ per gene, whereas large‐scale deletions occur at the genomic location of fragile sites with probability $\mu_{f}=10^{-2}$ per site.

We model individual *Streptomyces*‐like cells inhabiting a two‐dimensional surface, on which they replicate and secrete antibiotics. The genome organization of these virtual cells evolves through multiple cycles of vegetative growth and sporulation. Each cycle of vegetative growth starts with a population of germinating spores. Bacteria replicate locally, into empty lattice sites in their direct neighborhood. Due to replication and limited dispersal, related bacteria remain close to one another and form colonies (Fig 1A). Colonies develop for a fixed number of time steps $\tau_{s}$ (in each time step, all lattice sites are updated in random order)—after which we assume that resources are depleted and colonies sporulate. We model sporulation by randomly selecting a small fraction ξ of the bacteria (each cell is chosen with the same per‐capita probability), which will seed the next growth cycle. Spores do not disperse between cycles (i.e., there is no spore mixing). In practice, we end the growth cycle by killing at random a fraction 1−ξ of all the cells, leaving the remaining cells to initiate the next growth cycle.

Each bacterium possesses a genome that determines replication rate and antibiotic production. The model incorporates the mutational dynamics observed in the genome of *S. coelicolor*, as follows. We model the *Streptomyces* linear chromosome with a beads‐on‐a‐string model, which represents genomes as a linear sequence of genes and other genetic elements (Crombach & Hogeweg, 2007; Hindré *et al*, 2012). In addition to growth‐promoting and antibiotic production genes, we include fragile genomic sites that are mutational hotspots. These fragile sites can represent, e.g., long inverted repeats or transposable elements, that are common within bacterial genomes (Lovett, 2004; Darmon & Leach, 2014; Mei *et al*, 2021) and highly abundant in the subtelomeric regions of the Streptomyces chromosome (Chen *et al*, 2002). A genome can contain a variable number of genes and fragile sites. For simplicity, we ignore genes not directly involved in the division of labor (e.g., genes for central metabolism, homeostasis, and growth‐neutral genes). Because of this, a bacterium with no growth‐promoting genes remains alive, but it is assigned a growth rate equal to zero.

We make the simplifying assumption that the metabolic strategy of each cell, i.e., the amount of resources dedicated to growth vs. antibiotic production, is determined solely by its genotype—and ignore that these strategies may be regulated by density‐dependent or other secreted cues from other bacteria (Safari *et al*, 2014; Bednarz *et al*, 2019; Mukherjee & Bassler, 2019). Based on the finding that the deletion of part of the genome leads both to reduced growth and to increased antibiotic production (Zhang *et al*, 2020b), we assume that growth‐promoting genes are (partly) responsible for regulating the switch from primary to secondary metabolism. In order to focus on genome architecture and its mutational consequence, we also do not explicitly include gene regulation and assume that the metabolic switch between primary and secondary metabolism is genetically determined. Growth‐promoting genes inhibit the expression of antibiotic genes (i.e., regulation is fixed), and this inhibition is lifted when growth genes are in low numbers. The model abstracts away the details of how this regulation takes place and instead specifies a direct correspondence between the number of growth‐promoting genes g and replication rate $k_{\text{repl}}=\alpha_{g}\mathrm{Rg}/\left(g+h_{g}\right)$, and an inverse relationship between growth rate and antibiotic production $k_{\mathrm{ab}}=\alpha_{a}A\left(a\right)\exp\left(-\beta_{g}g\right)$ (plotted in Fig 1B; $\alpha_{g}$ is the maximum replication rate, R is a function that specifies the cell's resistance to the antibiotics it is in contact with (see Materials and Methods for details), $h_{g}$ is the number of growth‐promoting genes producing half maximum growth rate, $\alpha_{a}$ is the maximum antibiotic production rate, $A\left(a\right)$ is an increasing function of the number of antibiotic genes a in a genome, $\beta_{g}$ scales the inhibition of antibiotic production with the number of growth‐promoting genes). In summary, this assumption allows us to study the genetic contributions to the division of labor and neglect regulatory contributions. It results in a trade‐off because bacteria cannot simultaneously maximize both growth and antibiotic production, since the number of growth‐promoting genes g trades antibiotic production for replication. For low values of $\beta_{g}$, both growth and antibiotic production can be optimized at once, corresponding to an unrealistic situation where bacteria have arbitrary amounts of available energy. Therefore, $\beta_{g}$ must be sufficiently large to make the model realistic.

Bacteria can produce different antibiotics. The type of an antibiotic is determined by a bit‐string of length ν, so the total number of possible antibiotics is $2^{\nu }$. We set ν=16, which approximates the number of antibiotics produced by the genus *Streptomyces*, and ensures sufficient scope for the evolution of antibiotic diversity. We do not include a specific antibiotic resistance, e.g., arising from efflux pumps (Nag & Mehra, 2021). Antibiotics produced by a bacterium are secreted into its neighborhood, within a circle of radius $r_{a}=10$ lattice sites—as a proxy for diffusion. Multiple types of antibiotics can be present at each lattice site. To reduce computational load, we do not consider antibiotic concentration but only its presence or absence. The probability per time step of producing an antibiotic is proportional to $k_{\mathrm{ab}}$. Bacteria are resistant to antibiotics if they encode an antibiotic production gene with the same bit‐string in their genome. Resistance decreases when the difference between the two strings increases (see Materials and Methods for details). Cells die and are removed from the lattice, after contact with an antibiotic to which they have no resistance.

Mutations occur during cell division, when the genome is replicated, and consist of single‐locus (gene or fragile site) duplications and deletions with probability $\mu_{d}$ per locus, and fragile site‐induced mutations, occurring with probability $\mu_{f}$ per‐fragile site, that cause large deletions spanning from their genomic location to the right end of the chromosome (Fig 1C). We set $\mu_{d}\leq \mu_{f}$ because we assume that small‐scale duplications and deletions reflect the rare random mistakes occurring during DNA duplication, while fragile sites specifically enhance the likelihood of catastrophic failure of DNA replication, which results in large‐scale deletion. Fragile sites effectively introduce an asymmetry in the model genome, with the left part of the chromosome being mutationally more quiet than the right side. This mimics the increased telomeric deletion rate observed in the *Streptomyces* chromosome (Chen *et al*, 2002; Hopwood, 2006; Hoff *et al*, 2018; Tidjani *et al*, 2020), though we note three points: (i) We model only one arm of the *Streptomyces* chromosome, from the centromere to one telomere; (ii) in the model, the distinction between centromeric and telomeric regions depends solely on fragile sites, whereas *Streptomyces* telomeres are specific genomic structures that can be recognized independently from the mutational dynamics; (iii) in *Streptomyces*, fragile site‐induced mutations transiently cause a complex interplay of duplications and deletions, which only after several mutational events result in the large‐scale elimination of DNA (Altenbuchner & Cullum, 1984; Zhang *et al*, 2022)—here we simplify this complexity by letting activated fragile site delete the entire chromosome to their right. Novel fragile sites are spontaneously generated at random genomic locations with a small probability $\mu_{n}$ per replication. Moreover, antibiotic genes can mutate and diversify the antibiotics they encode. See Materials and Methods section for the details of the model, and Table 1 for parameter values used in all simulations (unless explicitly stated otherwise).

**Table 1:** Parameters used in the model: names, description, and default values.

Parameter	Explanation	Values
$L^{2}$	lattice size	300×300
η	neighborhood size for replication	8 (Moore neigh.)
$\tau_{s}$	Growth cycle duration	2,500 time steps
ξ	Fraction of spores to seed a growth cycle	0.001
*Replication*		
$\alpha_{g}$	max replication probability per unit time	0.1
$h_{g}$	nr. of growth genes for 1/2 max growth rate	10
$\beta_{r}$	antibiotic resistance factor	0.3
$\mu_{d}$	Duplication/deletion probability per gene	0.001
$\mu_{f}$	Fragile site‐induced deletion probability	0.01 (per‐fragile site)
$\mu_{n}$	Probability of new fragile site formation	0.01 per genome
$\mu_{a}$	Probability of antibiotic‐type mutation	0.005 (per ab gene)
*Antibiotic production*		
$\alpha_{a}$	max antib. production probability per unit time	1
$h_{a}$	nr. of antib. genes for 1/2 max production rate	3
$r_{a}$	max distance of antib. placement	10
$\beta_{g}$	antib. production decrease due to trade‐off	1
μ	length of antib. bit‐string	16
*Other parameters*		
$p_{\mathrm{mov}}$	prob. of migration into empty adjacent site	0.01

### Eco‐evolutionary dynamics of virtual *Streptomyces*

**Figure 2:**
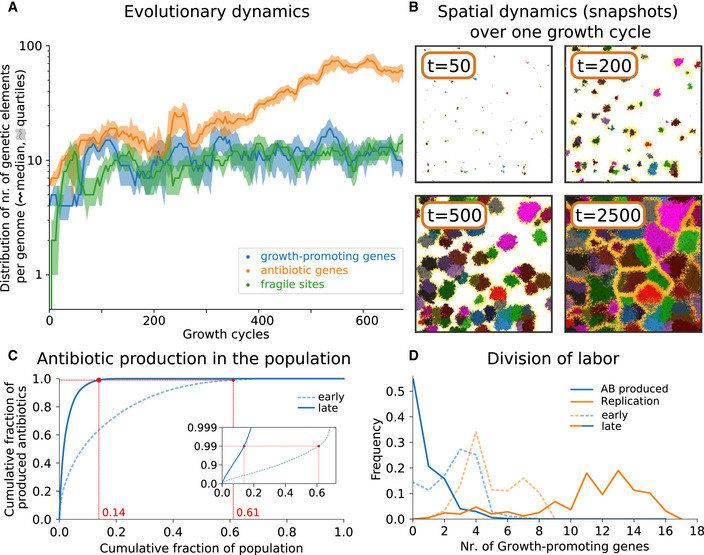
Evolution of genome composition and division of labor Evolutionary dynamics of gene content. For each time point, the median and quartile values of the number of each type of genetic element are calculated from all genomes in the population (at the end of the growth cycle). Growth cycle duration $\tau_{s}=2,500$ time steps. Notice the logarithmic y‐axis. The system is initialized with a population with genome: 5’‐FAAFAAFAAF‐3′, where F is a growth gene and A is an antibiotic gene, with each antibiotic gene encoding a different antibiotic type.Spatial dynamics during one growth cycle, after evolution: Different colors represent different colonies, yellow shading around the colonies indicates antibiotics, and time stamps in the pictures indicate the time elapsed in the growth cycle.Most antibiotics are produced by few bacteria after evolution. We collect all bacteria alive at the midpoint of the growth cycle of an early (after 40 cycles, i.e., $100\times 10^{3}$ time steps) and later stage (after 400 cycles, i.e., $1,000\times 10^{3}$ time steps) in the evolutionary dynamics (i.e., at 1,250 time steps). For each time point, bacteria are sorted on antibiotic production, the cumulative plot (blue line) is normalized by population size and total amount of antibiotics produced, and the red dot indicates the fraction of the population that produces 99% of all antibiotics. Inset: semi‐log plot of the same data.Division of labor between replication and antibiotic production, shown as a function of the number of growth‐promoting genes. Using the same data set as (C), the plot shows the frequency of replication events (orange) and antibiotic production events (blue) per number of growth genes, for early and later stages in evolution.

Starting from short genomes (initial size = 10 genes) without fragile sites, containing homogeneously distributed antibiotic genes and growth‐promoting genes, we let bacteria evolve over at least 500 vegetative growth cycles, with each growth cycle consisting of $\tau_{s}=2500$ time steps. Over this time, bacteria incorporate about 10 fragile sites in their genome (Fig 2A), evolve a large and diverse set of antibiotic genes (30–130 genes), and approximately 10 growth‐promoting genes (see Appendix Fig S1, Appendix Fig S2 for additional runs). Their genome architecture becomes highly nonhomogeneous. Growth‐promoting genes are progressively clustered to the right side of the chromosome. Fragile sites are initially incorporated at random genomic locations and evolve to localize to the left of growth‐promoting genes (Appendix Fig S3). After evolution, the spatial dynamics of the model over the course of one growth cycle qualitatively reproduce those of *Streptomyces* colony formation (see, e.g., pictures in Westhoff *et al*, 2020; Zhang *et al*, 2020b): Colonies expand and produce a growing halo of antibiotics and a corresponding zone of inhibition of competing strains. Colony expansion continues until colonies encounter antibiotics to which they are susceptible—i.e., antibiotics produced by other colonies. The invasion dynamics reach a quasi‐stable spatial configuration once all colonies have become enclosed by antibiotics from competing strains. At this stage bacteria born at the edge of the colony are killed by an adjacent colony's antibiotics (Czárán *et al*, 2002; Fig 2B; for a time course see Appendix Fig S4 top panes, and Movie EV1).

### Evolution of genome architecture leads to division of labor between replicating and antibiotic‐producing bacteria

To understand the population dynamics produced by the model, we compare populations after different stages in the simulations. At an early stage (after 40 growth cycles, i.e., $100\times 10^{3}$ time steps), antibiotics are collectively produced by the whole population. By contrast, at a later stage (400 cycles, i.e., $1000\times 10^{3}$ time steps) nearly all antibiotics are produced by a small fraction of the bacteria (Fig 2C, 99% of all antibiotics are produced by 61% of the population at time $100\times 10^{3}$, and by just 14% at time $1000\times 10^{3}$). We also observe that replication and antibiotic production are performed by genetically distinct bacteria in the later stages: Bacteria with few growth‐promoting genes produce most antibiotics but do not replicate frequently, whereas bacteria with a larger number of growth‐promoting genes replicate frequently but do not produce antibiotics (Fig 2D). Because antibiotic‐producing bacteria do not replicate, or replicate at very low rate, they cannot form an independent lineage and are instead generated in each growth cycle from the bacteria that have high replication rates. Therefore, antibiotic‐producing bacteria are found in irregular dotted patterns over the colony (Appendix Fig S4 bottom panes, and Movie EV2).

We characterize the fitness cost associated with generating these mutants by performing a series of competition experiments where evolved colonies (wildtype) are pitted against generalists with a broad range of antibiotic production and growth rates. These “artificial” generalists do not pay a fitness cost for being able to replicate and produce antibiotics at the same time, and thus do not experience a trade‐off. A generalist with similar growth and antibiotic production rate to a wildtype only barely outperforms the wildtype (Appendix Fig S5). This shows that the fitness cost associated with generating the mutants does not greatly affect colony competitiveness, in agreement with (Zhang *et al*, 2020b). A steeper trade‐off between replication and antibiotic production (corresponding to the metabolic shift from primary to secondary metabolism) makes the division of labor more likely to emerge. This is achieved when fewer growth‐promoting genes are required to inhibit antibiotic production (i.e., larger $\beta_{g}$; Appendix Fig S6), or when more growth‐promoting genes are required for faster growth (higher $h_{g}$; Appendix Fig S7). For $h_{g}\beta_{g}>5$, division of labor can evolve and the number of genes at steady state depends on the numerical value of the two parameters (Appendix Fig S8). Lower antibiotic production rate could be expected to further hinder the evolution of division of labor and instead favor the evolution of a generalist (because a specialized mutant with few growth genes makes fewer antibiotics, while the generalist is proportionally less affected). We find the opposite, instead: Division of labor evolves when bacteria produce fewer overall antibiotics (lower $\alpha_{a}$), under shallow trade‐off conditions ($h_{g}\beta_{g}=5$; see Appendix Fig S9). A lower overall antibiotic production thus broadens the range of trade‐off strength that allows division of labor to evolve. Altogether these results suggest that the requirements for evolving division of labor in *Streptomyces* might be rather broad, and therefore that it is likely to evolve in species other than *S. coelicolor*: It suffices that bacteria that grow rapidly cannot meet the antibiotic production requirements for the entire colony.

### Genome architecture supports the mutation‐driven division of labor

**Figure 3:**
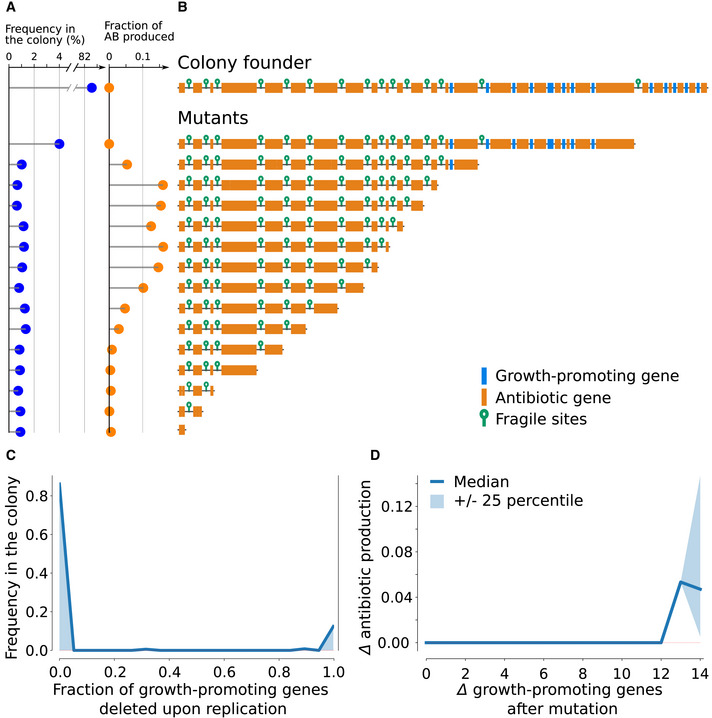
Genome architecture enables division of labor within an evolved colony: The colony founder generates antibiotic‐producing somatic cells through large‐scale chromosomal deletions, caused by fragile sites The data are generated by seeding a simulation with one bacterium—the colony founder—and letting the colony grow until it reaches a diameter of 70 lattice sites (which is the approximate colony size at the end of a growth cycle, cf. Fig 2B).Genomes in high abundance do not produce many antibiotics. All bacterial genomes are collected after colony growth, they are sorted by sequence length and they are left‐aligned to emphasize fragile site‐associated deletions. The bar plots contrast genome frequency in the colony (blue) and the fraction of antibiotics produced by bacteria with that genome (orange). Note the broken axis for genome frequency.Genome architecture of the colony founder and all its descendants. Growth‐promoting genes in blue, antibiotic genes in orange, and fragile sites depicted as green hairpins.Deletions of all‐or‐no growth‐promoting genes during replication. Shown is the fraction of growth‐promoting genes deleted because of fragile site instability upon replication during colony development (ranging from 0, i.e., no genes are deleted, and 1, all growth genes are deleted).Increased antibiotic production as a result of chromosomal deletions. Difference in antibiotic production as a function of the growth‐promoting genes lost during replication (colony median ± 25th percentile).

To understand the role of genome architecture in the division of labor, we extracted the founder of the most abundant colony after long‐term evolution and tracked its diversification after it was reinoculated into an empty grid (for ease of interpretation the only mutations we allow in this experiment are fragile site‐induced deletions). We observed that the genome of the evolved colony founder had two distinct regions: a region on the right of the chromosome that contains growth‐promoting genes but lacks fragile sites, and a region on the left side that lacks growth‐promoting genes and has abundant fragile sites (Fig 3B). After colony growth from this founder, nearly all the bacteria capable of replicating are genetic copies of the founder, while most of the antibiotic production is carried out by a diverse suite of mutants arising through genomic instabilities (Fig 3A). When bacteria divide, mutations induced at fragile sites lead to the deletion of the genomic region downstream (to the right) of their location. Because growth‐promoting genes are over‐represented in these regions, they are frequently deleted as a group (Fig 3C). Mutants generated from these deletions lack growth‐promoting genes while retaining many antibiotic genes, and will therefore produce antibiotics at much higher rates (Fig 3D). By this process, colonies eventually evolve to become functionally differentiated throughout the growth cycle. On average, a colony contains 2–7% mutants with large deletions that specialize in antibiotic production while foregoing replication themselves (Appendix Fig S10). In Appendix Figs S11 and S12, we show this genome architecture is prevalent in the evolved population.

**Figure 4:**
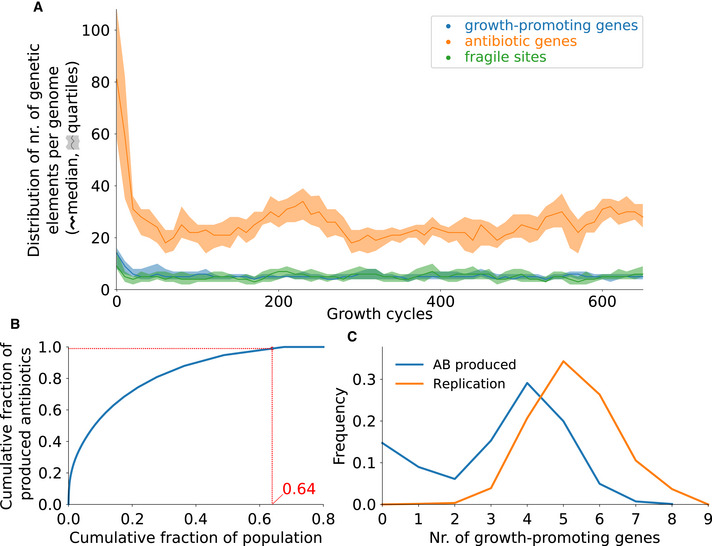
Genome shuffling between growth cycles leads to bacteria with small genomes that do not divide labor Evolutionary dynamics of the genome composition. As in Fig 3, the median and quartile values of the number of each type of genetic element are calculated from all genomes in the population, at the end of every growth cycle. Growth cycle duration $\tau_{s}=2,500$ time steps. We start the simulation from an evolved genome capable of mutation‐driven division of labor.Cumulative antibiotic production as a function of the cumulative fraction of the population. Bacteria from the midpoint of the growth cycle are sorted on antibiotic production (after evolutionary steady state is reached). The curve is normalized by population size and total amount of antibiotics produced. In red we indicate the fraction of the population that produces 99% of all antibiotics.Frequency of replication (orange) and antibiotic production (blue) as a function of the number of growth‐promoting genes. The same data are used as in (B).

To assess whether genome architecture is required for division of labor, we initiated an evolutionary run where we shuffled the order of the genes in the genome of each spore at the beginning of every growth cycle. This disrupts the genomic architecture of colony founders without changing its composition, in terms of the number and type of genes and fragile sites. Starting from an evolved genome, we observe a rapid decrease in antibiotic genes and in growth‐promoting genes, resulting in small genomes (Fig 4A). At an evolutionary steady state, a large fraction of the population contributes to antibiotic production (Fig 4B). Because these bacteria do not divide labor through mutation (Fig 4C), they can only express sub‐optimal levels of both growth‐promoting genes and antibiotic genes. In other words, they are generalists that do not resolve the trade‐off but compromise between replication and antibiotic production. Importantly, these evolved generalists always lose in direct competition experiments with bacteria that can divide labor when the genome is not mixed (section 9 of the Appendix). We conclude that genome architecture is a key prerequisite for the maintenance of a mutation‐driven division of labor in the model, and therefore that genome architecture is subject to natural selection.

We also studied what genome architecture evolves when additional genes are introduced that are essential for survival but are not directly involved in the division of labor (e.g., homeostatic genes). We modeled these genes as an additional gene type and assumed that they must be present in at least $n_{h}=10$ copies for the cell to remain alive. Any number of genes lower than $n_{h}$ is lethal and the bacterium carrying such genome is removed from the lattice, while a larger number is neutral. We find that the mutation‐driven division of labor evolves, and that the centromeric part of the evolved genomes contains always more than $n_{h}$ homeostatic genes, ensuring the survival of the antibiotic‐producing species that arise through mutation (Appendix Fig S13).

### Effect of spatial competition dynamics and antibiotic diversity on evolution of division of labor

We next examined the effect of spatial structure on the evolution of division of labor in our model. Previous work showed that spatial structure can promote the coexistence and diversity of antibiotic‐producing cells because antibiotics secreted in a cell's neighborhood prevent invasion by adjacent strains (Pagie & Hogeweg, 1999; Czárán *et al*, 2002; Kerr *et al*, 2002; Vetsigian *et al*, 2011; Abrudan *et al*, 2015; van Dijk & Hogeweg, 2016; Vetsigian, 2017). In our model, antibiotic‐based competition occurs at the boundaries of two colonies. Formation of colony boundary requires sufficiently long growth cycles (≥1000 time steps; Appendix Fig S14). Consequently, no division of labor evolves when cycles are shorter because selection only favors growth. Growth cycles shorter than 500 time steps lead to extinction as populations do not recover after sporulation. To further test the importance of competition at colony interfaces, we can perturb the spatial structure by mixing the system after each time step. Evolved bacteria maintain mutation‐driven division of labor in such a mixed system (Appendix Fig S15), but division of labor did not evolve *de novo* from a simulation initialized with random genomes (Appendix Fig S16).

We find that colonies produce a large number of different antibiotics, which likely results from a combination of selection for diversity (in line with Czárán *et al*, 2002) in the genes upstream of the fragile site cluster, and genetic drift in the genes downstream of it (Appendix Fig S17). Moreover, the large number of antibiotic genes in the evolved genomes is likely made possible by the simplified mutational dynamics we implemented, which allow new antibiotic types to evolve from pre‐existing antibiotic genes through single mutations. When the total number of possible antibiotics is large (about $6.5\times 10^{5}$ different antibiotics, see Materials and Methods), colonies are highly susceptible to the antibiotics produced by other colonies (Appendix Fig S18). The spatial dynamics allow for the coexistence of multiple strains because they mutually repress each others' invasion through the local production of antibiotics (Appendix Fig S4). With a smaller number of possible antibiotics, bacterial genomes evolve to contain every possible antibiotic gene, which results in low susceptibility (Appendix Fig S19, also cf. Pagie & Hogeweg, 1999). Mutation‐driven division of labor evolves as long as the number of possible antibiotics is not too small (Appendix Fig S20 and Appendix Fig S21). Colonies also evolve division of labor when the deposition radius around the producing bacterium is much reduced (the deposition radius is a proxy for diffusion; Appendix Fig S22). This reduces the benefit afforded by antibiotics because a single antibiotic‐producing bacterium can protect fewer colony members. Division of labor does not evolve when the antibiotic deposition radius is unrealistically small, and instead growth rate alone is maximized (similarly to genomes that evolve with short growth cycles, see Appendix Fig S14). The benefit of antibiotic production can also be reduced by decreasing the specificity of bacterial resistance to antibiotics (lowering $\beta_{r}$, see Materials and Methods). However, division of labor evolves when specificity is decreased by more than two orders of magnitude (Appendix Fig S23).

### Fragile site mutation rates co‐evolve with division of labor

**Figure 5:**
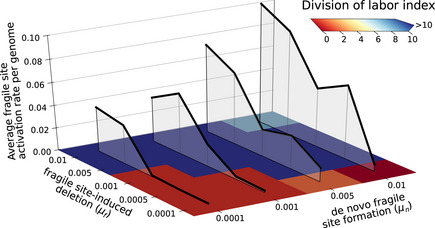
Mutation‐driven division of labor and mutation rates evolve over a wide range of fragile site mutation rates The plot shows the average genomic mutation rate (black lines) and the degree of division of labor (red to blue tiles) for a series of populations evolved under different $\mu_{n}$ (probability of *de novo* fragile site formation), and $\mu_{f}$ (fragile site‐induced deletion probability). Each tile shows the difference between the median numbers of growth‐promoting genes in antibiotic producers and replicating bacteria, after evolution has reached a steady state (after at least 600 growth cycles). The genomic mutation rate is calculated from the number of fragile sites f, as $\sum_{i=0}^{i=f}\mu_{f}{\left(1-\mu_{f}\right)}^{i}$.

Two parameters control the influx and activity of fragile sites: the rate of *de novo* fragile site formation $\mu_{n}$ and the fragile site‐induced deletion probability $\mu_{f}$. We assess the robustness of our results when these two parameters are systematically varied. In Fig 5, we characterize the effect of mutation rates on the evolution of division of labor with a “division of labor index” (defined as the difference between the median number of growth‐promoting genes for replicating bacteria and antibiotic producers). A larger value indicates a larger genetic distance between the cells that replicate and those that produce antibiotics, and thus a stronger division of labor. We find a sharp boundary between mutation rates that allow the evolution of division of labor (blue tiles) and mutation rates for which division of labor does not evolve (red tiles; see Appendix Fig S24 for more data). The evolution of division of labor is accompanied by the evolution of the high rate of genome deletion (black line in Fig 5). A larger number of fragile sites is incorporated in the genomes when each fragile site has a smaller probability of causing deletion (Appendix Fig S25). The evolution of division of labor also results in the incorporation of a larger number of growth‐promoting genes. When division of labor does not evolve, genomes contain few growth‐promoting genes, so that each bacterium grows and produces antibiotics. Bacteria that do not evolve division of labor behave like generalists and are similar to those evolved with genome shuffling, see Fig 4. However, once division of labor evolved with $\mu_{n}>0$, it persists in the evolved populations when $\mu_{n}$ is set to zero (Appendix Fig S26).

## Discussion

We studied how an association between genome architecture and division of labor evolves, inspired by recent experimental findings in *Streptomyces* (Zhang *et al*, 2020b). We constructed a computational model of *Streptomyces* colony development where cells have a linear genome with mutational hotspots, and experience a trade‐off between replication and antibiotic production. We showed that replicating bacteria can exploit mutations to generate a new cell type that specializes in antibiotic production, thereby resolving the trade‐off between these traits (Fig 2). This mutation‐driven differentiation is coordinated by an evolved genome architecture with two key features: fragile sites that destabilize the genome by causing large‐scale deletions, and an over‐representation of growth‐promoting genes at the unstable chromosome end. Because of this organization, mutations at fragile sites preferentially delete growth‐promoting genes, leaving mutant genomes containing only genes for antibiotic production. Although these cells are unable to grow, their terminal differentiation into antibiotic‐producing cells is advantageous to the colony as a whole. Altogether, the model provides a novel hypothesis for the evolution of the observed mutation‐driven division of labor in *Streptomyces*.

*Streptomyces* genomes contain a conserved (mutationally quiet) core region located at their centromere and telomeric regions, which are prone to various forms of recombination and instability and contain much of the accessory genes (Kirby, 2011; Kim *et al*, 2015). This leads to high within‐ and between‐species variability at the chromosome ends (Lorenzi *et al*, 2021). Both fragile sites (including genomic inverted repeats and other recombination hotspots, associated with intrachromosomal recombination and with mobile genetic elements) and biosynthetic clusters are unevenly distributed along the chromosome of *Streptomyces* (Kim *et al*, 2015; Tidjani *et al*, 2019; van Bergeijk *et al*, 2020), suggesting that they are positioned non randomly. Our model suggests that telomeric instabilities have evolved to coordinate the division of labor between replication and antibiotic production, explaining recent experimental results in *S. coelicolor* (Zhang *et al*, 2020b). Thus, these instabilities may not be accidental byproducts of a linear chromosome or other aspects of unstable replication (Chen *et al*, 2002); our model suggests that they are instead an evolved and functional property of *Streptomyces* genomes. Based on the robustness of our results to parameter changes, and because genome instabilities are extremely common in *Streptomyces*, we expect that other species in the genus also divide labor through this mechanism. For instance, recent results show that the expression of biosynthetic pathways in *S. ambofaciens* is related to DNA folding (Lioy *et al*, 2021). Based on our model, it is tempting to speculate that fragile site‐induced deletions can change DNA folding by removing parts of complementary DNA, exposing genes that were previously hidden to the transcription machinery. All these lines of evidence support the idea of the evolution of a precise genome architecture that increases the evolvability and the dispensable nature of the telomeres—providing support for the idea that the genome architecture of the telomeres in *Streptomyces* results from an evolutionary interplay between biosynthetic genes and fragile sites.

*Streptomyces* produce a broad diversity of metabolically expensive compounds like cellulases and chitinases that can also be considered public goods (Chater *et al*, 2010). The production of these compounds is also expected to trade‐off with growth, and may, therefore, also be sensitive to division of labor through genome instability. Such examples of division of labor could alternatively be organized through differential gene expression—as is common in multicellular eukaryotes, eusocial insects, and other microbes. This is currently precluded in our model because for simplicity, we did not include the evolution of gene regulation. Division of labor, when selected for, can only be achieved through the evolution of genome architecture. An advantage of the mutation‐driven division of labor presented here is that it makes social conflicts (West & Cooper, 2016) impossible because altruistic somatic cells do not possess the genetic means to reproduce autonomously and participate in social dynamics (Frénoy *et al*, 2017). Validating these predictions will require detailed experiments in other species, as well as bioinformatic analyses of *Streptomyces* genome structures. These analyses may then inform a more detailed model of *Streptomyces* division of labor, accounting for the interplay between genome organization, gene regulation, and the metabolic network, underpinning the trade‐off between growth and antibiotic production.

The genes for an antibiotic biosynthesis pathway are usually clustered in the genome of *Streptomyces* and include genes for antibiotic resistance (Liu *et al*, 2013). Incorporating this genomic organization in the model would generate a more realistic mutational landscape for biosynthetic genes. For instance, it would allow to study the evolutionary consequences of acquiring resistance genes decoupled from the biosynthetic pathway of the cognate antibiotic (Peterson & Kaur, 2018), which would lead to a kind of “cheating” behavior as the resistant bacterium would not pay the cost of producing antibiotics. Interestingly, novel gene acquisition including antibiotic resistance occurs predominantly via horizontal transfer (Pettis, 2018), which is known to also drive mutational hotspot formation in *Streptomyces* (Tidjani *et al*, 2019). Moreover, exposure to some antibiotics induces DNA damage and significantly increases instability (Volff *et al*, 1993a,b). Preliminary experiments have found that antibiotic production and genome instability increase in some species of *Streptomyces* during competition between colonies (Chopra & Rozen, Unpublished data). This suggests that the sensing of antibiotics increases mutation rates at contact points between colonies, so that mutants appear preferentially where colonies compete with one another. Investigating the consequences of this additional layer of evolutionary dynamics will be interesting in future extensions of the model. A more complete model of the *Streptomyces* genome could also include both telomeres and their complex recombination dynamics (Tidjani *et al*, 2020) (only one telomere was considered here for simplicity), as well as an additional gene type (and the regulation) for sporulation, which was not explicitly included in this work. Besides better capturing the developmental dynamics of *Streptomyces*, this extension to the model would make the evolutionary dynamics more complex, e.g., by allowing a broader range of phenotypic or genetic mechanisms of resistance evolution.

Computational models indicate that the organization of genetic information along the chromosome is influenced by the mutational operators that act on it (Hogeweg & Hesper, 1992; Crombach & Hogeweg, 2007; Colizzi & Hogeweg, 2019; van Dijk *et al*, 2022). As a consequence of this organization, mutations may be more likely to generate mutant offspring with specific characteristics, such as reduced competition with the wildtype due to low fitness (Sanjuán *et al*, 2004; Eyre‐Walker & Keightley, 2007; Sarkisyan *et al*, 2016; Rutten *et al*, 2019), lower propensity for social conflicts (Frénoy *et al*, 2013) or accelerated re‐adaptation to variable environments (Crombach & Hogeweg, 2008). In yeast, whole chromosome duplications increase survival to stress (Yona *et al*, 2012). Taken together, these examples show that evolvability can be shaped by genome architecture—which in turn is the result of evolution (Hogeweg, 2012). In an Origin of Life model, it was found that mutants could provide a benefit to the wildtype. There a germline RNA replicator evolved whose mutants were sterile but altruistically replicated the germline and protected it from parasites (Colizzi & Hogeweg, 2014). Building on these earlier results, our model shows that a genome architecture can evolve to incorporate mutational hotspots, exploiting mutations to generate functional phenotypes and divide labor.

In prokaryotes, mutational operators that can drive functional mutagenesis include horizontal gene transfer, which drives the rapid evolution of gene content (Madsen *et al*, 2012; van Dijk & Hogeweg, 2016; Stalder *et al*, 2020; van Dijk *et al*, 2020), the CRISPR–Cas system, that generates immunity to viral infections through targeted incorporation of viral genomes (Makarova *et al*, 2020), and so‐called Diversity‐Generating Retroelements that accelerate the evolution at specific genomic sites and can generate functional diversity (Macadangdang *et al*, 2022). Reversible intrachromosomal recombination allows phenotype switching in *Staphylococcus* (Guérillot *et al*, 2019), and fragile site‐associated DNA deletions are more common at the replication terminus of circular bacterial genomes (Mei *et al*, 2021), further highlighting the interplay between mutation and genome organization. Beyond *Streptomyces*, mutation‐driven division of labor occurs in the genome of many ciliates, where functional genes must be carefully excised from a transposon‐riddled genomic background before being transcribed (Bracht *et al*, 2013; Yerlici & Landweber, 2014). Programmed DNA elimination in somatic cells is common in multicellular eukaryotes (Wang & Davis, 2014), and targeted recombination is essential for the functioning of the adaptive immune system in vertebrates (Flajnik & Kasahara, 2010). These examples highlight the ubiquity of functional mutagenesis across the tree of life. Our model may therefore serve as a proof‐of‐concept to understand the evolutionary origin of division of labor through functional mutagenesis more broadly.

## Materials and Methods

The model is a lattice‐based stochastic simulation system. We consider a population of bacteria that can replicate and produce antibiotics. The model is inspired by the life cycle of *Streptomyces coelicolor*, and focuses on the hyphal growth phase when colonies develop and compete by producing antibiotics (Claessen *et al*, 2014). We therefore model the eco‐evolutionary dynamics occurring during several colony growth cycles, each of fixed duration $\tau_{s}$. At the beginning of each cycle, spores germinate and colonies begin to form through bacterial replication. If antibiotics are produced, they are deposited around the producing bacterium, forming a halo that protects the colony and “reserves” space to replicate into. Bacteria die if they come into contact with antibiotics to which they are sensitive, and therefore bacteria of a colony cannot invade into the antibiotic halo of another one—if they are genetically different. At the end of the cycle, corresponding to the sporulation phase in *Streptomyces*, a small random sample ξ of the population is selected to form spores. These spores seed the next cycle, and other bacteria and antibiotics are removed from the lattice. Spores are deposited at the same location where the bacterium lived (we do not shuffle the location of the spores) unless explicitly stated.

We model bacteria and antibiotics on two separate lattices $\Lambda_{1}$ and $\Lambda_{2}$. Both lattices have size L×L and toroidal boundaries to avoid edge effects. Every site of $\Lambda_{1}$ can either be occupied by one bacterium or be empty. Every site of $\Lambda_{2}$ can be occupied by multiple antibiotics. Each bacterium possesses a genome that determines its replication rate, as well as the rate and type of antibiotics it produces and is resistant to. The genome consists of a linear sequence of three types of genetic elements (so‐called beads‐on‐a‐string model; Crombach & Hogeweg, 2007; Hindré *et al*, 2012). We consider two gene types—growth‐promoting genes and antibiotic genes. The third type of genomic element is a fragile genomic site—a hotspot for large chromosomal deletion. Growth‐promoting genes increase replication rate and inhibit antibiotic production, in accordance with *Streptomyces* growth being favored over secondary metabolism. Antibiotic genes encode both the toxin and its resistance (Mak *et al*, 2014). Antibiotic type is encoded in the genetic sequence of the antibiotic gene. This sequence is modeled as a bit‐string of fixed length ν, which can be mutated to encode different antibiotics. Each bacterium can have multiple growth‐promoting and antibiotic genes, as well as multiple fragile sites.

### Replication

Replication rate $k_{\text{replication}}$ depends on the intrinsic replication rate function G and on the resistance R of the bacterium to antibiotics in the spatial location in which it lives:

$$k_{\text{replication}}=\mathrm{GR}\left(\text{antibiotics}\right).$$

We do not explicitly model the regulation of gene expression, and instead, we let the intrinsic replication rate $G\left(g\right)$ directly increase with the number of growth‐promoting genes g. We assume no growth with zero growth genes $G\left(g=0\right)=0$, and that growth cannot be arbitrarily large even if the genome hosts many growth genes, i.e., $\lim_{g\to \infty }G\left(g\right)=\alpha_{g}$, where $\alpha_{g}$ is the maximum growth rate. A simple function that satisfies these requirements is a Hill function (a hyperbole):

$$G\left(g\right)=\alpha_{g}\frac{g}{g+h_{g}},$$

The additional parameter $h_{g}$ controls the curvature of the function and is equal to the number of growth‐promoting genes producing half maximum replication rate: $G\left(h_{g}\right)=\alpha_{g}/2$. We expect that other functions that phenomenologically reproduce G, e.g., an exponential function of the form $G\left(g\right)=\alpha_{g}\left(1-\exp\left(-g/h_{g}\right)\right)$, will not change the results.

Antibiotic resistance R depends on whether a genome has at least an antibiotic gene sufficiently similar to the antibiotics present on $\Lambda_{2}$ at the corresponding location of the bacterium, where similarity is determined from the bit‐strings of the antibiotics and the antibiotic genes. For each antibiotic n on $\Lambda_{2}$, the Hamming Distance D (the number of different bits) between the antibiotic and the gene with the minimum distance from n is calculated. All these minimum distances are summed into a susceptibility score S=∑D, and the overall resistance is a decreasing function of S:

$$R=e^{-\beta_{r}S^{2}}.$$

Each bacterium is resistant to the antibiotics it produces, because for each antibiotic D=0, and thus R=1. Moreover, the Gaussian function ensures that small mutations in antibiotic types do not decrease resistance too rapidly.

Replication occurs when bacteria are adjacent to an empty site on $\Lambda_{1}$. These bacteria compete on the basis of their probability of replication. For each competing bacterium i, the probability of replication is $k_{\text{replication}}\left(i\right)/\eta$, with η the neighborhood size (we use the Moore neighborhood throughout this study, thus η=8). Dividing by η scales the probability of replication so that a bacterium with maximum replication rate surrounded by empty sites replicates on average once per time step. Upon replication, the new bacterium inherits the genome of its parent, with possible mutations.

### Mutations

Mutations occur during replication, and can expand or shrink genomes, and diversify antibiotics.

#### Duplications and deletions

Duplication and deletion of genes and fragile sites occur with equal per‐gene probability $\mu_{d}$. When a gene or a fragile site is duplicated, the copy is inserted at a random genomic location. This ensures that gene clustering is not a trivial consequence of neutral mutational dynamics, and must instead be selected upon to evolve. We expect that there is an upper limit for $\mu_{d}$ above which the genome becomes effectively scrambled (therefore no genome structure could evolve) because fast duplications randomize gene position and fast deletions decrease local genomic correlations.

#### Fragile site large chromosome deletions

Fragile sites are the cause of genome instability in the model. We let fragile sites cause large‐scale chromosomal mutations with a per‐fragile site probability $\mu_{f}$. We take into account that large‐scale mutations in *Streptomyces* preferentially disrupt telomeric regions (Chen *et al*, 2002; Hopwood, 2006; Hoff *et al*, 2018; Tidjani *et al*, 2020) by letting fragile site‐induced mutations delete the entire chromosomal region downstream (i.e., to the right) of the genomic location of the fragile site (see Fig 1C). Effectively, this means that we model one arm of the chromosome, and that the model centromere and telomere result from the asymmetric effect of fragile site deletions. No other type of mutation has any left/right preference in the model.

#### Mutations of the antibiotic genes

The antibiotic genes consist of a genetic sequence modeled as a bit‐string of length ν. Mutations flip bits with a uniform per‐bit (per antibiotic gene) probability $\mu_{a}$. This changes the antibiotic type, and thus the antibiotic repertoire of the bacterium.

#### Influx of new fragile sites

Fragile genomic sites, such as inverted repeats or transposable elements are common in the genome of *Streptomyces*. Because they are easily copied (or translocated) we assume that they can also be spontaneously generated with a small probability $\mu_{n}$ (independent of genome size). The new fragile site is inserted at a random location in the genome.

### Antibiotic production, in a trade‐off with replication

Antibiotic production rate is modeled as an increasing function A of the number of antibiotic genes a a bacterium has. In analogy with the function derived for replication, we model antibiotic production as a Hill function. At the same time, production is strongly inhibited by growth‐promoting genes—a function $I\left(g\right)$ with g the number of growth‐promoting genes in the genome, in accordance with a likely trade‐off between growth and secondary metabolism (Zhang *et al*, 2020b). Antibiotic production rate, per time step, is the function:

$$k_{\mathrm{ab}\;\text{production}}=A\left(a\right)I\left(g\right)$$

with

$$\begin{array}{l} A\left(a\right)=\alpha_{a}\frac{a}{a+h_{a}},\\ I\left(g\right)=\exp\left(-\beta_{g}g\right). \end{array}$$

According to this function, the trade‐off becomes rapidly steeper with larger $\beta_{g}$. We expect that results would not qualitatively differ with if I was written in terms of a decreasing hyperbolic function, e.g., as $I=1/\left(1+\beta_{g}g\right)$. For simplicity, we assume that antibiotics are deposited at a random location within a circle of radius $r_{a}$ around the producing bacterium. Moreover, we do not take into account concentrations of antibiotics and only model the presence/absence of an antibiotic type in a spatial location (antibiotics of different types can be in the same location). Analogous to the Gillespie algorithm (Gillespie, 1976; see also methods in Takeuchi & Hogeweg, 2007; for the spatially extended version), antibiotic production is a first‐order (linear) process. As $k_{\mathrm{ab}\;\text{production}}$ takes values in $\left[0,1\right]$, we treat it as the probability of producing an antibiotic over one time step. We normalize the probability of antibiotic production by the area $A\left(r_{a}\right)$, i.e., the number of lattice sites in the circle of radius $r_{a}$ around the bacterium, and we draw the number of antibiotics deposited from a binomial distribution with parameters ($A\left(r_{a}\right)$, $k_{\mathrm{ab}\;\text{production}}/A\left(r_{a}\right)$). This makes antibiotic production independent from the deposition radius and allows us to compare results across simulations with different $r_{a}$. If a bacterium has multiple antibiotic genes, each antibiotic deposited is chosen randomly from the antibiotic genes with uniform probability.

### Death

Death can occur when a bacterium is sensitive to an antibiotic located at the same site as the bacterium itself. The probability of a bacterium dying is calculated as 1−R, where R is the bacterial resistance to the antibiotic defined above. When a bacterium dies, it is removed from the lattice, leaving behind an empty site.

### Movement

Bacteria have a small probability $p_{\mathrm{mov}}$ of moving if there is an empty site adjacent to them. This speeds up colony expansion and competition between colonies and avoids strong grid effects that could make spatial patterns too rigid.

### Initial conditions and updating of the dynamics

Unless differently specified, at time t=0 a small population of spores is seeded on the lattice. The initial spores have a small genome of length 10, i.e., a random sequence of growth‐promoting genes and antibiotic genes (of random types) but no fragile sites. The lattice is updated asynchronously: Over one time step, each lattice site is updated in random order.

The source code is written in c and uses the CASH libraries (de Boer & Staritsky, 2000). Analysis and plotting custom software are written in python. Genome schematics in Fig 3B are partly drawn with dnaplotlib (Der *et al*, 2017).

## Author contributions

**Enrico Sandro Colizzi:** Conceptualization; software; formal analysis; investigation; methodology; writing – original draft; writing – review and editing. **Bram van Dijk:** Conceptualization; software; investigation; methodology; writing – original draft; writing – review and editing. **Roeland M H Merks:** Supervision; investigation; methodology; writing – review and editing. **Daniel E Rozen:** Supervision; investigation; methodology; writing – review and editing. **Renske M A Vroomans:** Conceptualization; software; supervision; investigation; methodology; writing – review and editing.

## Disclosure and competing interests statement

The authors declare that they have no conflict of interest.

## Supporting information



Appendix S1

Movie EV1

Movie EV2

## Data Availability

The computer code produced in this study is available in the following databases: source code: strepto2 (https://github.com/escolizzi/strepto2).
