# Supplementary material for: Evolution of genome fragility enables microbial division of labor
Source: Mol Syst Biol. 2023 Feb 2;19(3):e11353. doi: 10.15252/msb.202211353 (PMC9996244; doi:10.15252/msb.202211353)
Supplement: Supplementary file 1 — Appendix S1 [file MSB-19-e11353-s001.pdf]

Appendix to: Evolution of genome fragility enables microbial division of labor

E.S. Colizzi*, B. van Dijk, R.M.H. Merks, D.E. Rozen, R.M.A. Vroomans

* corresponding author: enricosandro.colizzi@slcu.cam.ac.uk

Contents

1 The evolutionary dynamics of genome composition **4**
Appendix Figure S1 5
Appendix Figure S2 6
Appendix Figure S3 8

2 Snapshot of the eco-evolutionary dynamics within one growth cycle **9**
Appendix Figure S4 10

3 The mild fitness costs of dividing labor through mutation **11**
Appendix Figure S5 13

4 The evolution of division of labor depends on the trade-off between antibiotic production and replication - the effect of β_g and h_g **14**
Appendix Figure S6 15
Appendix Figure S7 17

5 A scaling relationship between growth and antibiotic production controls trade-off strength **18**
Appendix Figure S8 19

6 **Weaker trade-off and lower overall antibiotic production enable division of labor** **20**
Appendix Figure S9 21

7 **The fraction of mutants during colony development** **22**
Appendix Figure S10 23

8 **Genome architecture: growth-promoting genes** **24**
Appendix Figure S11 24
Appendix Figure S12 25

9 **Competition between wildtype and genomes evolved with shuffling** **26**

10 **Evolution of genome architecture with an additional gene type** **27**
Appendix Figure S13 27

11 **Division of labor evolves if colonies have sufficient time to develop and compete** **28**
Appendix Figure S14 29

12 **The effect of destroying spatial structure** **30**
Appendix Figure S15 30
Appendix Figure S16 31

13 **The number of antibiotic genes is partly due to selection for diversity** **32**
Appendix Figure S17 33

14 **High and diverse antibiotic production** **33**
Appendix Figure S18 34

15 **The total number of possible antibiotics determines the evolution of colony susceptibility** **35**
Appendix Figure S19 36

16 **The architecture of genomes evolved when antibiotic volume space is small** **37**

Appendix Figure S20 37
Appendix Figure S21 38

17 Division of labor evolves when the deposition zone of antibiotics is smaller and when the resistance to antibiotics is broader 39
Appendix Figure S22 40
Appendix Figure S23 42

18 Mutation-driven division of labor evolves over a wide range of (per-fragile sites) mutation rates. 43
Appendix Figure S24 44

19 The genome composition of populations that evolve division of labor over a wide range of (per-fragile site) mutation rates. 45
Appendix Figure S25 45

20 Division of labor persists in evolved genomes when de-novo fragile site formation is set to zero 46
Appendix Figure S26 46

1 The evolutionary dynamics of genome composition

For clarity, in the main text we showed only one example of the evolutionary dynamics. Appendix Figure S1 shows that qualitatively the same evolutionary trends are followed when the experiment is repeated. The figure also shows that population size at the end of each growth cycle remains approximately constant at about 60000 individuals over evolutionary time.

The mutation-driven division of labor described in the main text is robustly driving the eco-evolutionary dynamics also when we run the system for a very long time (2000 growth cycles), see Appendix Figure S2. Interestingly, we also observe large fluctuations in the number of antibiotic genes.

In Appendix Figure S3, we show the evolutionary dynamics of genome architecture for the simulation presented in main text Fig. 2. We extract the genome of all the individuals at the end of growth cycle 0, 20, 40, 100, 400 (corresponding to 0, 50000, 100000, 250000, 1000000 time steps), and plot, for each time step, the distribution of the position of each gene type on the chromosome. Because genome size is highly variable in the population, we normalize the position of each gene by the genome length, so that position 0 corresponds to the most centromeric region, and 1 corresponds to the most telomeric region. The system is initiated with genomes consisting of growth-promoting genes and antibiotic genes (without fragile sites, see caption of main text Fig. 2). These genomes replicate slowly because they have few growth-promoting genes, and produce some antibiotics, but cannot divide labor because they lack fragile sites. The number of growth-promoting genes, antibiotic genes and fragile sites increases rapidly at an early stage of the simulation. A larger number of growth-promoting genes results in faster-replicating genomes, indicating selection for growth. We observe that the genomic location of growth-promoting genes shifts towards the right of the chromosome in the first 100 growth cycles. As fragile sites are incorporated in the genome, their locations are initially random, but over evolutionary times their

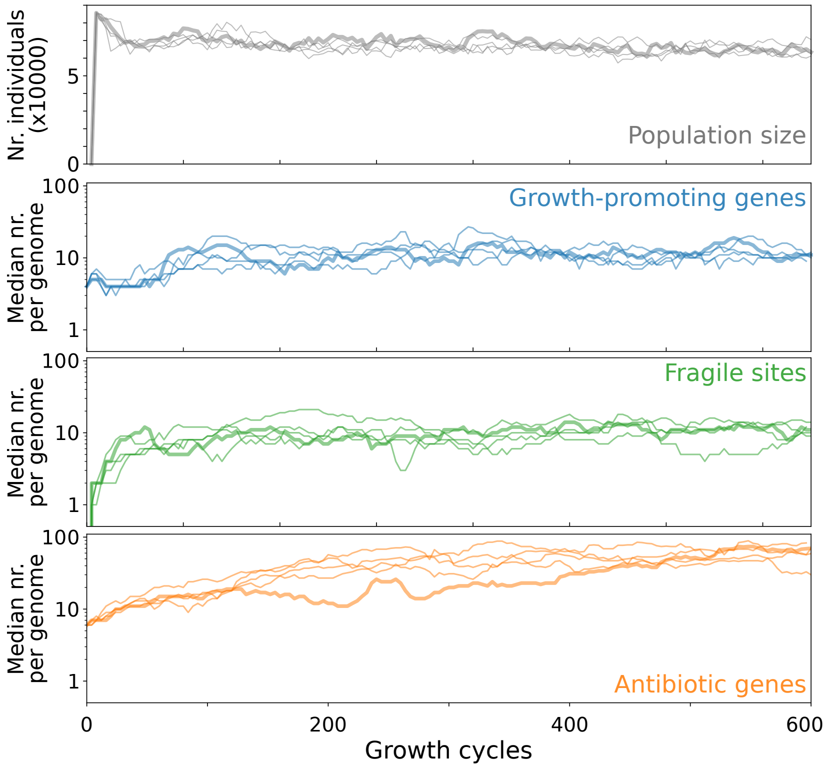

Appendix Figure S1: Evolutionary dynamics of population size and gene content, in five independent evolutionary runs. The run used in the main text is indicated with a thicker line. All the runs are initialized with a population with genome: `5′-FAAFAAFAAF-3′`, where F is a growth-promoting gene and A is an antibiotic gene, with each antibiotic gene encoding a different antibiotic type. Top plot shows the total population size at the end of each growth cycle. The other plots show the median number of each type of genetic element, calculated from all genomes in the population at the end of every growth cycle. Growth cycle duration $\tau_s = 2500$ time steps.

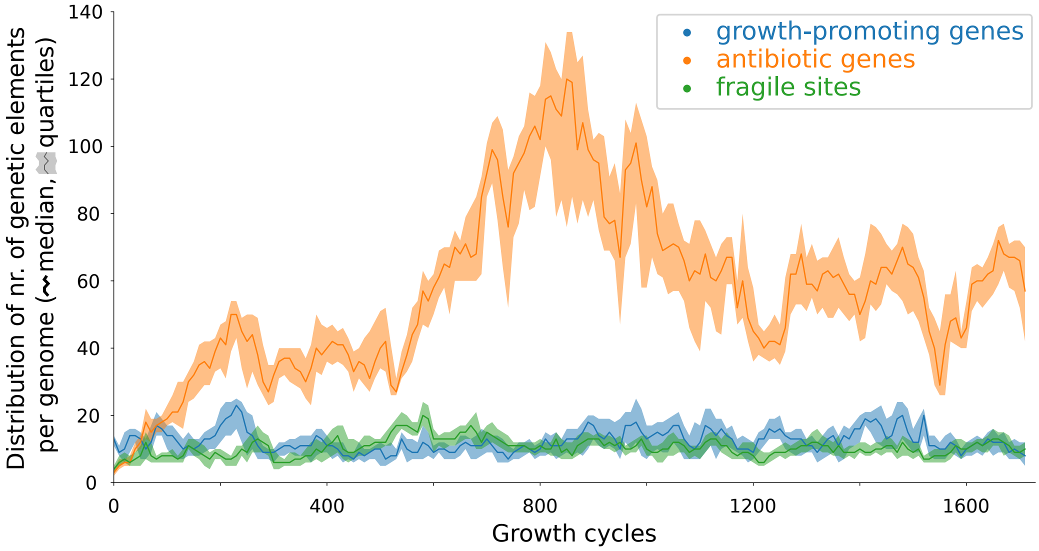

Appendix Figure S2: Very long-term evolutionary dynamics of genome composition. The median and quartile values of the number of each type of genetic element are calculated from all genomes in the population, at the end of every growth cycle. All parameters are the same as in 2a. The system was initialized with a small population of randomly generated genomes of length = 20 consisting, on average, of 65% growth-promoting genes, 15% antibiotic and 20% fragile sites. Antibiotic type was also randomly generated. Growth cycle duration $\tau_s = 2500$ time steps.

genomic position becomes more anti-correlated with that of growth-promoting genes. This enables a progressively more efficient division of labor, because more growth-promoting genes are removed due to fragile sites deletions. The resulting bacteria lacking growth-promoting genes can express their antibiotic genes, which benefits the colony. This suggests that higher antibiotic production is selected for (also see spatial dynamics of bacterial colonies and antibiotics in main text Fig. 2b). The genomic location of antibiotic genes does not change dramatically over evolutionary time (over evolutionary time they become slightly more frequent at the left side of the chromosome). However, their number becomes very large, and by growth cycle 400, the population has optimized the genome structure to locate almost all growth-genes to the right of the chromosome. This results in high antibiotic production by mutants (main text Fig. 2c) which lack growth-promoting genes (main text Fig. 2d).

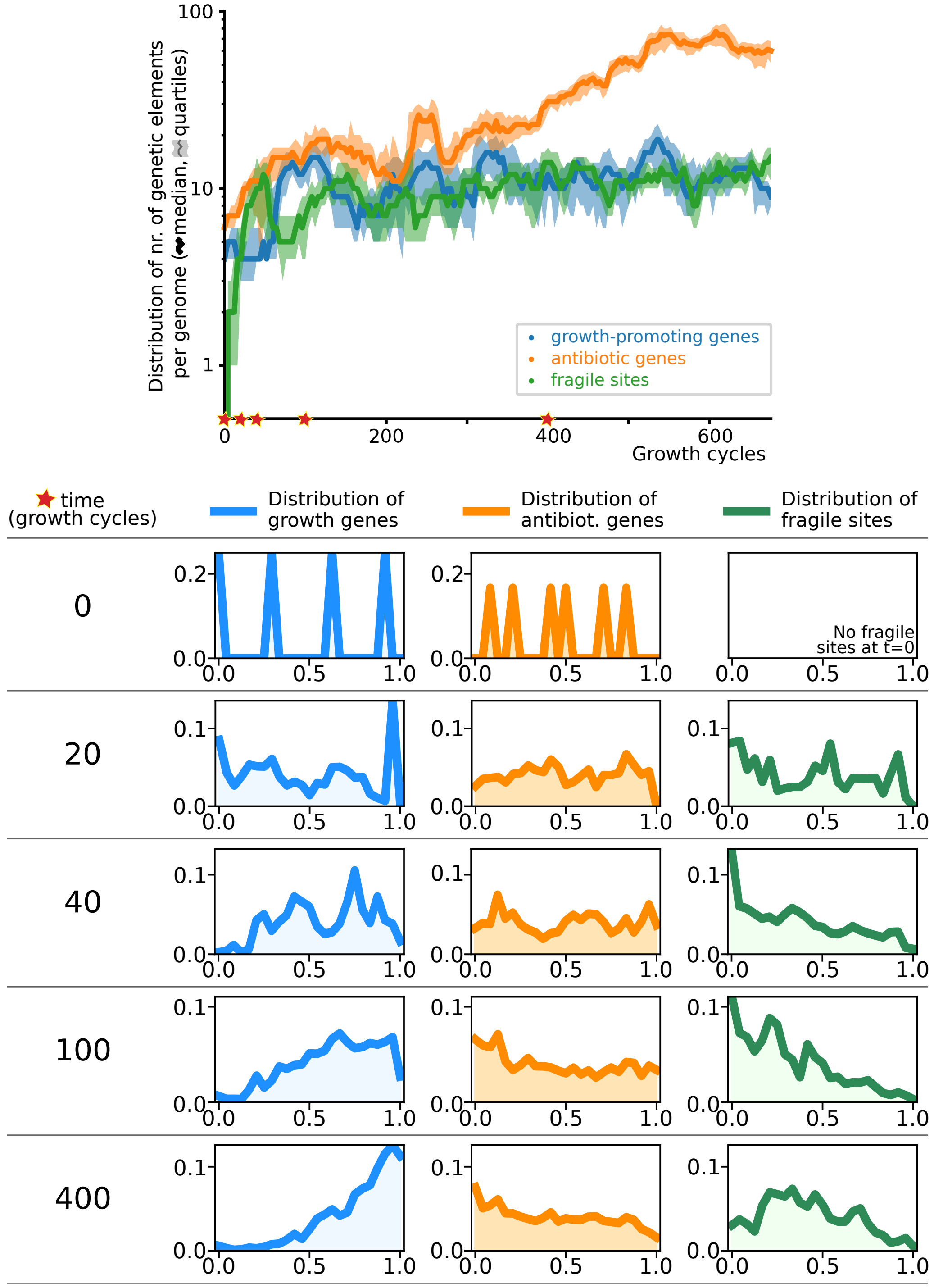

Appendix Figure S3: The evolution of genome architecture. **Top**: evolutionary dynamics of gene content (this figure is the same as main text Fig. 2a). The time points chosen for genome architecture analysis are indicated on the x-axis with a star. **Bottom**: The evolution of genome architecture in the population. Starting from genomes without fragile sites, and with a homogeneous distribution of growth-promoting and antibiotic genes, the system evolves to genomes that compartmentalize growth-promoting genes to the left of fragile sites.

2 Snapshot of the eco-evolutionary dynamics within one growth cycle

Appendix Figure S4 shows successive snapshots of the lattice, over the course of a growth cycle that lasts 2500 time steps. Starting from spores, colonies expand and produce antibiotics. Antibiotic-producing mutants form a dotted pattern over the colony. For each time point, the top pane shows colonies and antibiotics, the bottom pane shows antibiotic potential in non-producing cells (higher with darker blue) and antibiotic-producing cells (red).

Note that some colonies are not dividing labor, as they do not contain individuals that produce antibiotics. These colonies have a smaller number of antibiotic genes (indicated by a lighter shade of blue), and they seem to grow slower (their final colony size is smaller than the others) so they likely have fewer growth genes which do not completely inhibit antibiotic production. These alternative strategies can arise occasionally through mutations and survive for a while because colonies only compete once their antibiotic haloes are in contact, but go extinct within a few growth cycles.

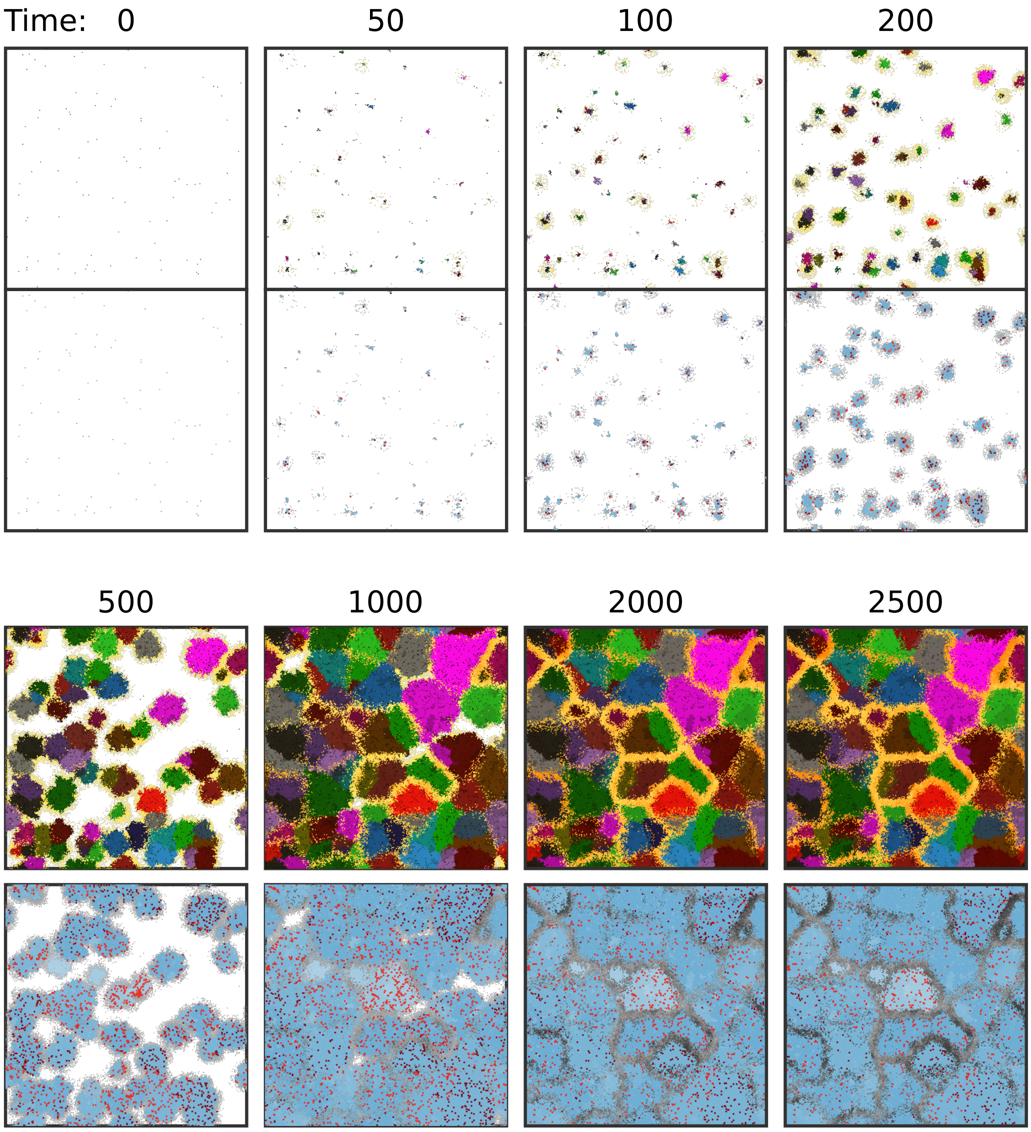

Appendix Figure S4: The eco-evolutionary dynamics during a growth cycle. For each time point, top pane: different colors represent colonies arising from single spores, darker shades of yellow indicate that more antibiotics are present; bottom pane: darker shades of blue indicates larger number of antibiotic genes in non-producing (and weakly producing) cells, red dots indicate antibiotic-producing bacteria (brighter red corresponds to higher production), gray corresponds to antibiotics. In both panes, white is background.

3 The mild fitness costs of dividing labor through mutation

In this section we investigate the fitness cost associated with division of labor, i.e. the fitness loss associated with having one phenotype replicating and another producing antibiotics. To do this, we perform several competition experiments between wildtype bacteria that divide labor through mutations (which we evolved in previous simulations), and "artificial" generalists whose replication rate and antibiotic production rate are drawn from a broad spectrum of values. If an artificial generalist with small replication rate and small antibiotic production rate were to outcompete the wildtype, then the fitness cost associated to dividing labor would be large. Conversely, if an artificial generalist grows as fast as the wildtype when both their replication rate and their antibiotic production rate are similar, then the wildtype is not paying a fitness cost for dividing labor.

We chose three independently evolved bacteria, and repeat each competition experiment four times. We initialize the system with a mix of the two genotypes, and we run the simulation with mutation rates μ_d, μ_n, μ_a set to zero (so that no further evolution happens), and with the probability of fragile-site deletion μ_f set to default values (so that division of labor can occur). After 15 growth cycles, the winner of the competition is determined. Appendix Figure S5 shows the sum of how many times the artificial generalist (+1) or the wildtype (-1) wins, for each combination of the generalist's growth and antibiotic production rate. A draw occurs in some cases (0), when both genotypes are present in similar numbers after 15 cycles. The figure also shows the replication rate and antibiotic production rate of the wildtype species we used (yellow bar). For these, replication rate was calculated from the number of growth-promoting genes in their genome (see Methods), while average and standard deviation of per-capita antibiotic-production rate was obtained from a simulation which was initialized with only this bacterium, and all mutation rates except μ_f were set to zero (to avoid evolution).

Appendix Figure S5 shows that the wild type wins over the artificial generalist when the former produces more antibiotics and/or when it grows faster. Competition results in a near draw when the artificial generalist replicates at the same rate as the wildtype, and produces antibiotics at the same rate as the wild type's mutants. This means that the fitness cost associated with division of labor is small.

The three wildtype genomes are as follow (A: antibiotic genes; F: growth-promoting genes; B: fragile sites):

```
    >Wildtype Genome 1:  nr.  F=14, nr.  A=111, nr.  B=15
AABAAABABAAAAAAAAAAAABAAAAAABAAAAABAAAAAAAABAAAAAABAABABAABA
AAABAABAFAAAAAAAAABFAAAAAAAFAAAAFAAAAFFAAFAFAAAAAFAAAAAAAAAAA
AABAAFAAAFAFAAFAAFAA

    >Wildtype Genome 2:  nr.  F=21, nr.  A=61, nr.  B=10
AAAAAAAAAABAAABAAAAAABAABABABBBAAFABAAAAAAFFAAAAAAAFAAAFAFFA
AAFFAFFFBAFAAFFAFAFFAFAAAAAFAAAF

    >Wildtype Genome 3:  nr.  F=15, nr.  A=71 , nr.  B=14
AAAABAABAAAAAAAAABAAABAAAAAFAAABBAAABAAAABABAABAAAAAFFFBAABA
FABAFAAAAAFFAFAAFAFAAAABAFAAAAFAFAAAFAAA
```

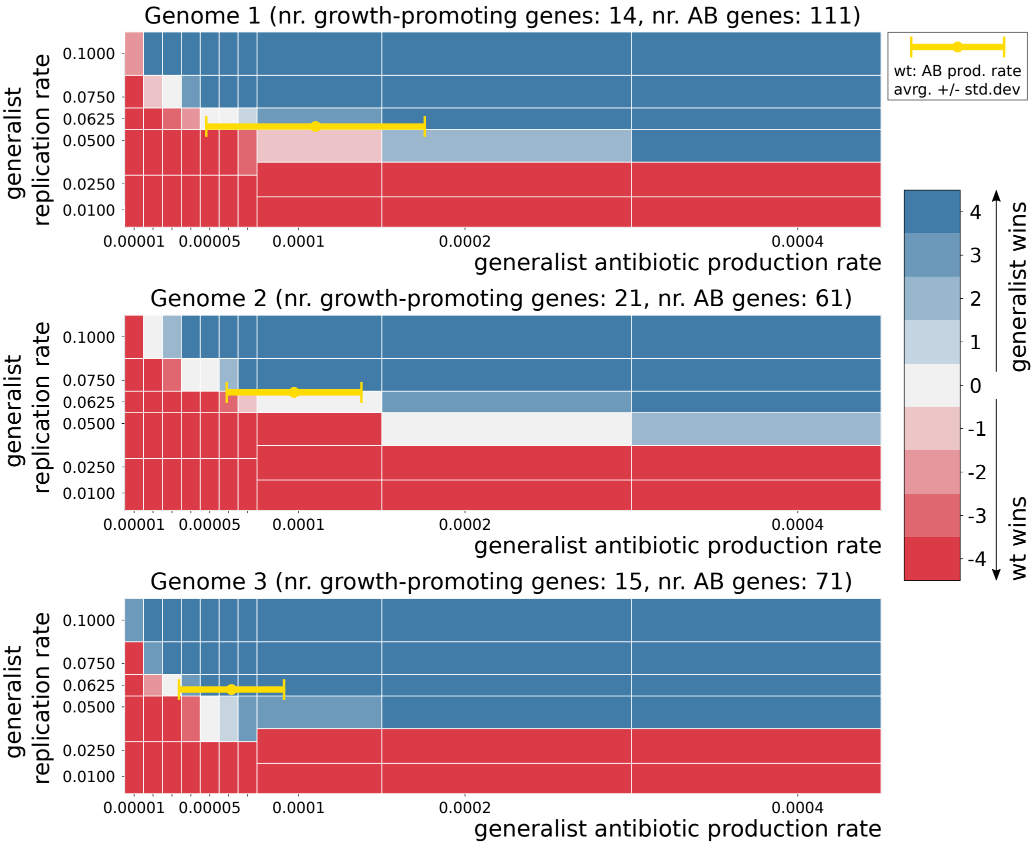

Appendix Figure S5: Competition experiments between artificial generalists and three evolved wild type species. The plot shows the number of times the generalist or wildtype wins, given different values of growth rate and antibiotic production rate of the generalist. Blue: majority generalist wins. Red: majority wildtype wins. Grey: majority draws or equal number of wins.

4 The evolution of division of labor depends on the trade-off between antibiotic production and replication - the effect of β_g and h_g

A model assumption is that the number of growth-promoting genes negatively affect antibiotic production and positively affect replication. These effects are controlled by two parameters, (respectively) β_g and h_g. In the following two paragraphs, we check that results reported in the main text are robust to changes in these parameters. We do this by systematically varying each of these two parameters independently (while in the following Suppl. Section 5 we vary both parameters at the same time).

Larger β_g favors division of labor Antibiotic production rate k_{ab} is inversely related to the number of growth-promoting genes via the equation $k_{\text{ab}} = A\exp(-\beta_g g)$ (see Methods) - effectively imposing a trade-off between replication and antibiotic production. The strength of the trade-off can be tuned by the parameter β_g, which in the main text is set to $\beta_g = 1$ throughout. The closer β_g is to zero, the more antibiotic production becomes independent of the number of growth genes. Very small β_g corresponds to an unrealistic situation where bacteria have arbitrary energy to maximize both replication and antibiotic production. We expect that no division of labor evolves in this case. Indeed, Appendix Figure S6 shows that for $\beta_g < 0.5$, division of labor does not evolve. The figure also shows that for $\beta_g = 0.5$ division of labor occasionally evolves - but it is evolutionarily unstable (not shown). Division of labor evolves for larger values of β_g (i.e. for $\beta_g = 0.75$ and 1), i.e. once there is a perceivable trade-off between antibiotic production and growth.

Interestingly, the plots also show that changing β_g does not affect the steady state composition of the genome. As shown below and in the main text, parameters controlling mutations and growth have a larger influence on genome composition.

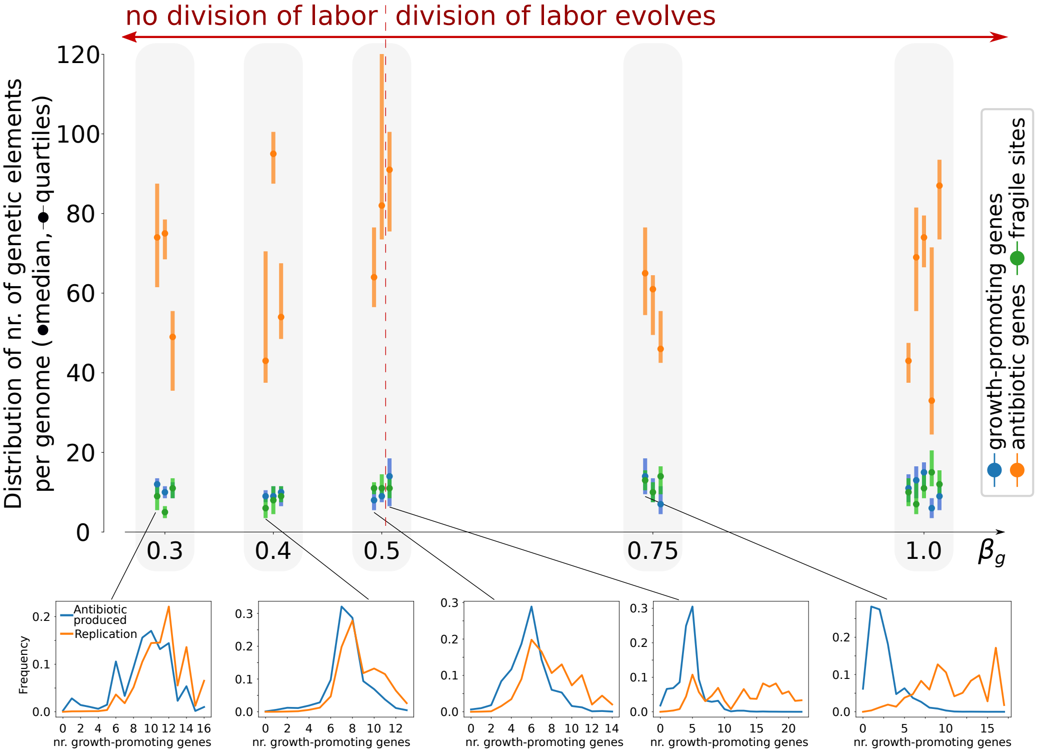

Appendix Figure S6: Division of labor evolves when inhibition of antibiotic production is sufficiently strong with a large nr. of growth-promoting genes (thus making the trade-off between replication and antibiotic production stronger). Data is collected from the entire population, for one growth cycle after long-term evolution (> 1000 growth cycles) from a series of simulations with different values of β_g (three replicas for each β_g; all other parameters are identical to those in the caption of 1). The top pane shows the distribution of each genetic element in the genomes. Bottom panes show the frequency of antibiotic producers (blue) and replicating individuals (orange) as a function of the nr. of growth-promoting genes, for the replica indicated in the figure. A larger difference between the two curves indicates division of labor, because the two tasks are carried by genetic distinct individuals in the same colony.

Larger h_g favors division of labor Replication rate depends on the number of growth genes in the model, via the equation $G(g) = \alpha_g \frac{g}{g+h_g}$. The parameter h_g sets the number of growth-promoting genes g that result in half-maximum growth rate, i.e. increasing h_g results in a lower growth rate for a fixed value of g. Trade-off strength can be increased with larger h_g, because larger g will be required for growth, which results in a decrease in antibiotic production rate. Indeed, Appendix Figure S7 shows division of labor evolves for $h_g \geq 6$. For $h_g = 4$ division of labor evolves, but we observe that it is evolutionarily unstable.

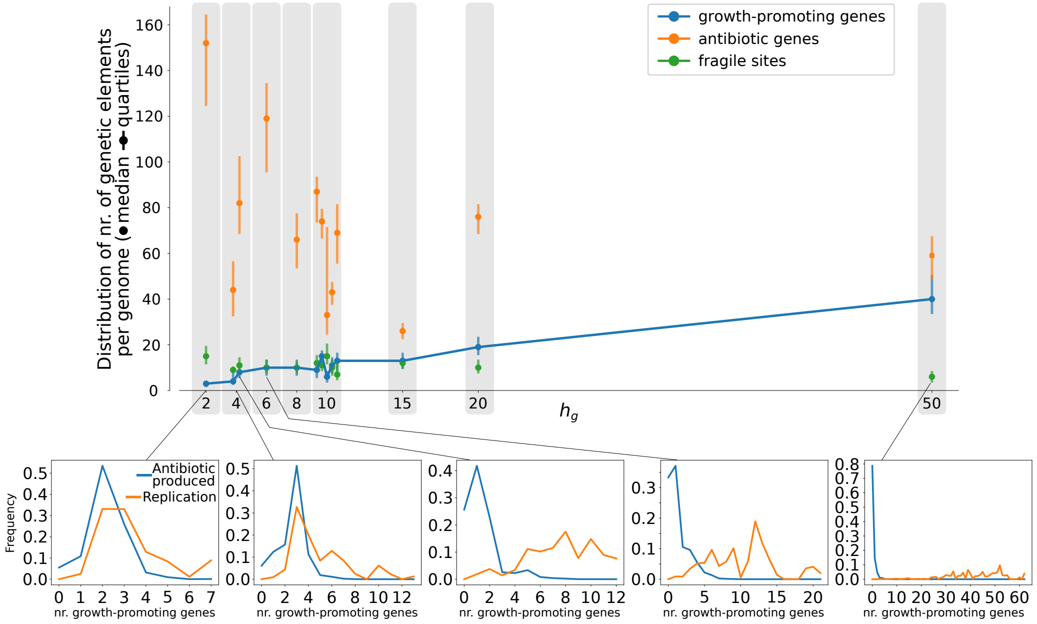

Appendix Figure S7: Division of labor evolves when the nr. of growth genes required for half-maximum growth is sufficiently large, making the trade-off between replication and antibiotic production stronger. Data is collected from the entire population, for one growth cycle after long-term evolution ($>$ 1000 growth cycles) from a series of simulations with different values of h_g (all other parameters are identical to those in the caption of 1). The top pane shows the distribution of each genetic element in the genomes. Bottom panes show the frequency of antibiotic producers (blue) and replicating individuals (orange) as a function of the nr. of growth promoting genes, for the replica indicated in the figure. A larger difference between the two curves indicates division of labor, because the two tasks are carried by genetic distinct individuals in the same colony.

5 A scaling relationship between growth and antibiotic production controls trade-off strength

In the section, we better characterize the model robustness to parameter changes by studying which combinations of parameters affect its behavior (as opposed to individual parameters). Specifically, we focus on the trade-off between replication and antibiotic production. In the previous section (Suppl. Section 4) we discussed that changing either β_g or h_g changed the strength of the trade-off between growth and antibiotic production. Here, we keep the trade-off strength constant by simultaneously changing β_g and h_g so that the ratio between growth and antibiotic production remains the same.

To do this, we write the growth function as: $G(g') = \alpha_g \frac{g'}{g'+1}$, where $g' = g/h_g$, showing that growth rate as a function of g is measured in units of $(1/h_g)$. Similarly, antibiotic production (as a function of g) can be written as $I(g'') = \exp(-g'')$, where $g'' = \beta_g g$, showing that antibiotic production rate as a function of growth-promoting genes is measured in units of β_g. Therefore, $g = g' h_g = g''/\beta_g$, and thus the ratio between growth and antibiotic production remains the same if we set β_g and h_g so that their product $K = h_g \beta_g$ is constant.

Since changing either parameter changes trade-off strength (see Suppl. Section 4), but setting $\beta_g = K/h_g$ does not, we conclude that the product $K = \beta_g h_g$ scales trade-off strength. Indeed, Appendix Figure S8 shows that division of labor evolves when we change the values of β_g and h_g maintaining their product to $K = 10$ (as in main text results).

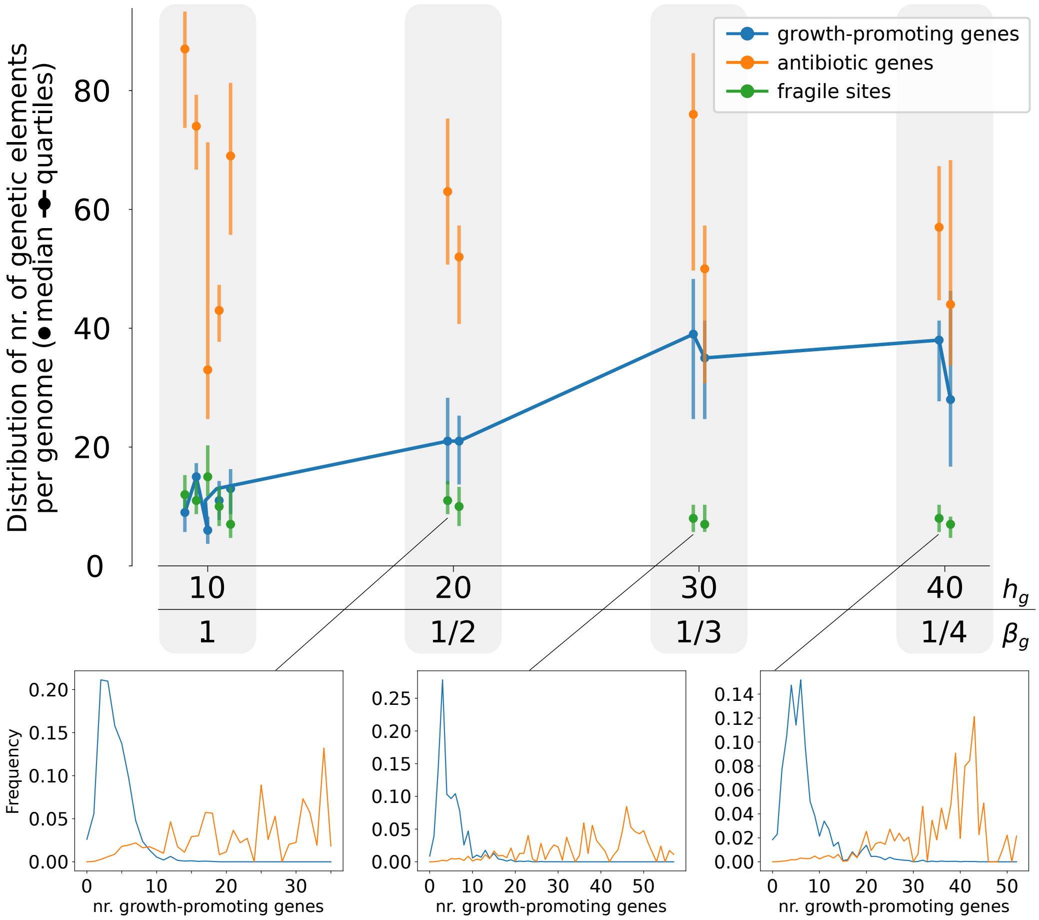

Appendix Figure S8: Trade-off strength is kept constant - and division of labor can evolve - by scaling the nr. of growth genes required for half-maximum growth h_g with the nr. of growth genes required for inhibition of antibiotic production β_g. Data is collected from the entire population, for one growth cycle after long-term evolution (> 1000 growth cycles) from a series of simulations with different values of h_g and β_g such that $h_g \beta_g = 10$ (all other parameters are identical to those in the caption of 1). The top pane shows the distribution of each genetic element in the genomes. Bottom panes show the frequency of antibiotic producers (blue) and replicating individuals (orange) as a function of the nr. of growth promoting genes, for the replica indicated in the figure. A larger difference between the two curves indicates division of labor, because the two tasks are carried by genetic distinct individuals in the same colony.

6 Weaker trade-off and lower overall antibiotic production enable division of labor

In this section we further explore the robustness of our results to parameter changes. Specifically, we suggest that the condition for the evolution of division of labor is that fast replicating individuals cannot satisfy the antibiotics demand of the colony. This also means that trade-off strength $K = \beta_g h_g$ (see Suppl. Section 4) is not the only determinant of the evolution of division of labor.

The overall antibiotic production rate is controlled by the parameter α_a. When α_a is smaller, fewer antibiotics are produced by bacteria, even when they have few growth-promoting genes. This might be expected to result in a lower benefit of generating specialized mutants with few growth genes, hindering division of labor, as a generalist is proportionally less affected by lower α_a.

Surprisingly, we show in Appendix Figure S9 that smaller values of α_a enable division of labor when the trade-off is shallower (i.e. when $\beta_g h_g$ is small). We set $h_g = 10$, as in main text, and $\beta_g = 0.5$, resulting in $\beta_g h_g = 5$, which makes the trade-off too shallow for division of labor to robustly evolve (under default values of α_a). We further set $\alpha_a = 0.075$, which is more than an order of magnitude smaller than default value (the specific value of α_a is chosen so that the antibiotic production rate for an intermediate nr. of growth promoting genes ($g = 5$) is the same as that obtained with default values. In formulas, $I(\alpha_a = 0.075, \beta_g = -0.5, g = 5) \approx I(\alpha_a = 1, \beta_g = -1, g = 5)$).

This shows that a broader range of trade-off strengths allow for the evolution of division of labor when overall antibiotic production is smaller. These results suggest that division of labor can evolve under mild conditions, as it suffices that replicating individuals cannot make enough antibiotics for the colony, and is thus a likely outcome of the evolutionary dynamics of antibiotic-producing microbes.

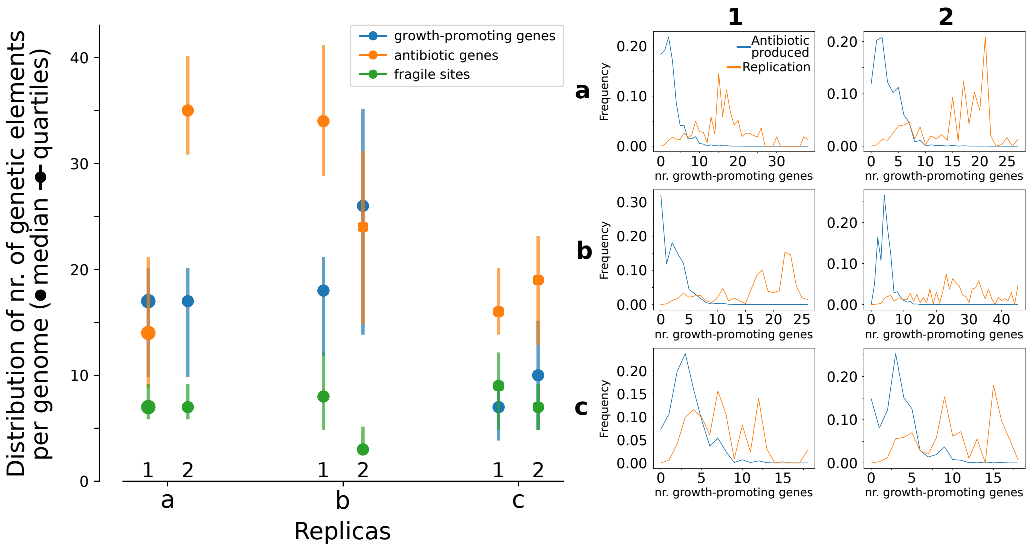

Appendix Figure S9: Weak trade-off and low antibiotic production allow for evolution of division of labor. Three independent simulations (a,b,c) are run with parameters $h_g = 10$ and $\beta_g = 0.5$ and $\alpha_a = 0.075$ (all other parameters are identical to those in the caption of 1). Data is collected from the entire population, for one growth cycle after long-term evolution from two data points (at growth cycle nr. 1600 and 2000). Measuring two data points (1 and 2) separated by a large number of growth cycles ensures that within each simulation division of labor is maintained over evolutionary time. The left pane shows the distribution of each genetic element in the genomes after long-term evolution. Right panes show the frequency of antibiotic producers (blue) and replicating individuals (orange) as a function of the nr. of growth promoting genes, for the replicas indicated in the figure. A larger difference between the two curves indicates division of labor, because the two tasks are carried by genetic distinct individuals in the same colony.

7 The fraction of mutants during colony development

As shown in the main text, colonies begin their growth cycle from single spores, and diversify through mutations. Mutants that overproduce antibiotics show massive deletions in their telomeres and lack growth promoting genes. We select mutants that deleted at least $3/4$ of their growth genes and have at least 1 antibiotic gene as a proxy for mutants that hyperproduce antibiotics (see main text Fig. 2d). Appendix Figure S10 shows how the fractions of these mutants changes during colony development. For colonies at an early time stage (i.e. at 40 growth cycles), mutation-driven division of labor has not evolved yet, and therefore mutations are largely deleterious and over a growth cycle mutants are outcompeted by the wild-type. At later stages, after division of labor has evolved, the fraction of mutants is much larger than for earlier cases reaching up to 7% of the population at the beginning of the growth cycle, and stabilizing above 2% at the end. The reason for this decrease in mutant population is that mutant offspring of the same wild type can have a different number of antibiotic genes (see main text Fig. 3). This makes mutants potentially susceptible to some of the antibiotics produced by other mutants in the same colony. Moreover, later in the growth cycle there are more antibiotics present in the grid. Once a mutant is killed, a wildtype bacterium can replicate to fill the lattice site left empty, and likely a wildtype offspring will take that site rather than another mutant. Thus there is a decline in the overall mutant population.

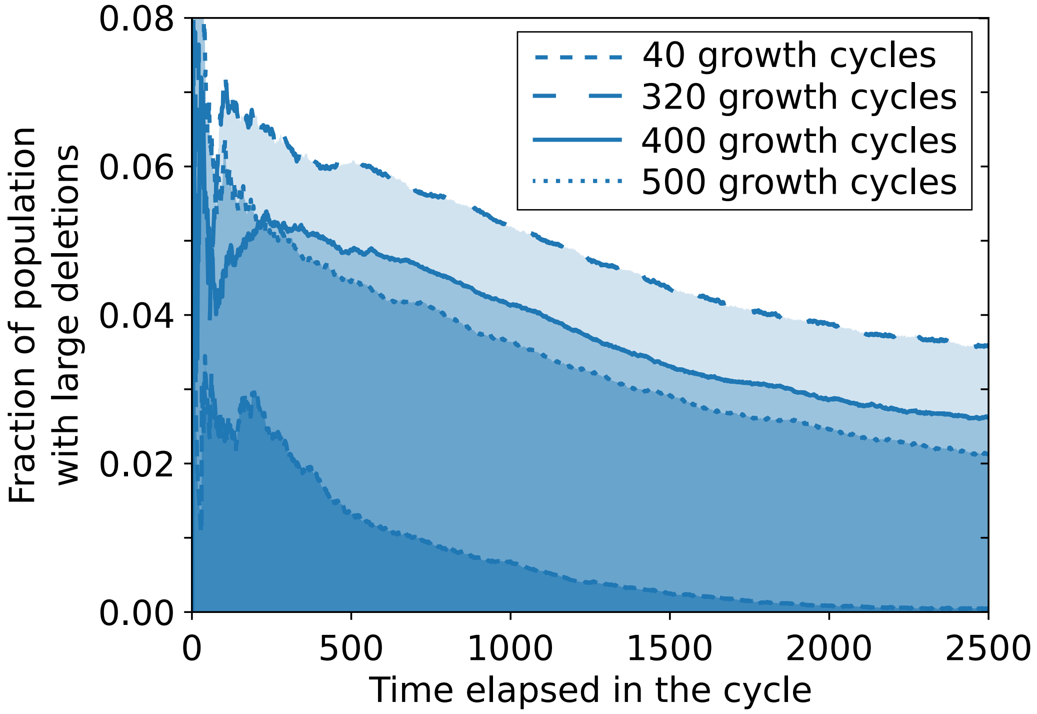

Appendix Figure S10: The fraction of mutants during colony development (i.e. over one growth cycle of 2500 time steps), for different time points. At growth cycle 40, division of labor has not evolved yet. At growth cycle 320, 400 and 500 division of labor has evolved and mutants are stably maintained throughout the growth cycle.

8 Genome architecture: growth-promoting genes

Appendix Figure S11 shows that the evolved genome architecture compartmentalizes growth-promoting genes to the telomeric region of the bacterial chromosome. While in the main text we presented one example colony, here we show that the evolved genome architecture is similar in the entire population. We extract the genome of all the individuals at the end of one growth cycle, after long-term evolution (more than 1000 growth cycles), from the simulation shown in Appendix Figure S2. Because genome size is highly variable in the population, we normalize the position of each gene by the genome length, so that position 0 corresponds to the most centromeric region, and 1 corresponds to the most telomeric region. We then generate a 2D histogram that correlates the position of each growth-promoting gene with the size of the genome. The 2D histgram shows that the evolved architecture is consistent across the population, despite large variation in genome size. To avoid correlation between genome size and number of growth-promoting genes, we normalize the contribution of each gene by the size of the genome it comes from.

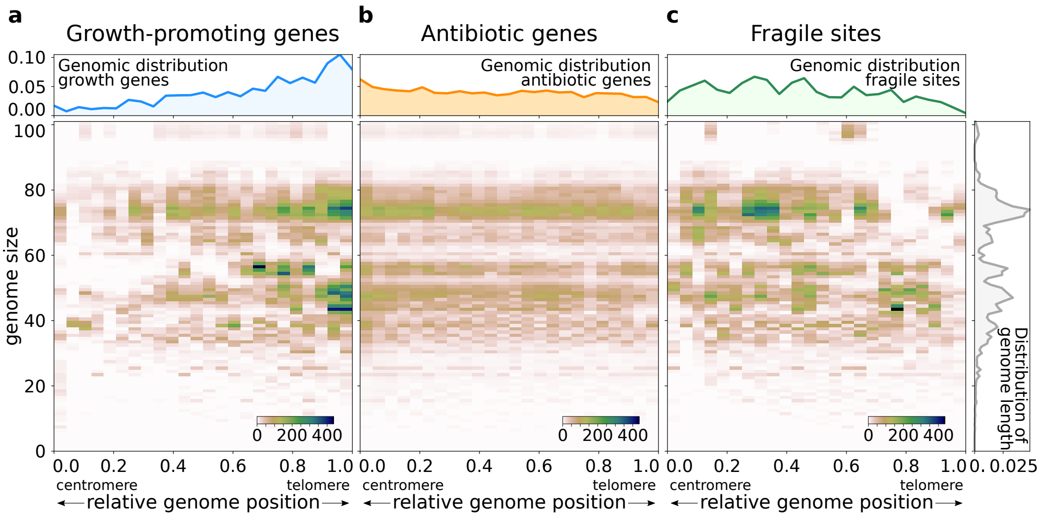

Appendix Figure S11: The evolved genome architecture: growth promoting genes are compartmentalized to the telomeric side of fragile sites, so that fragile-site deletions delete growth genes in block. Data from the last time step of the simulation shown in Appendix Figure S2.

In Appendix Figure S12, we also show the genome architecture extracted from the populations at the last time point of the five simulations shown in Appendix Figure S1.

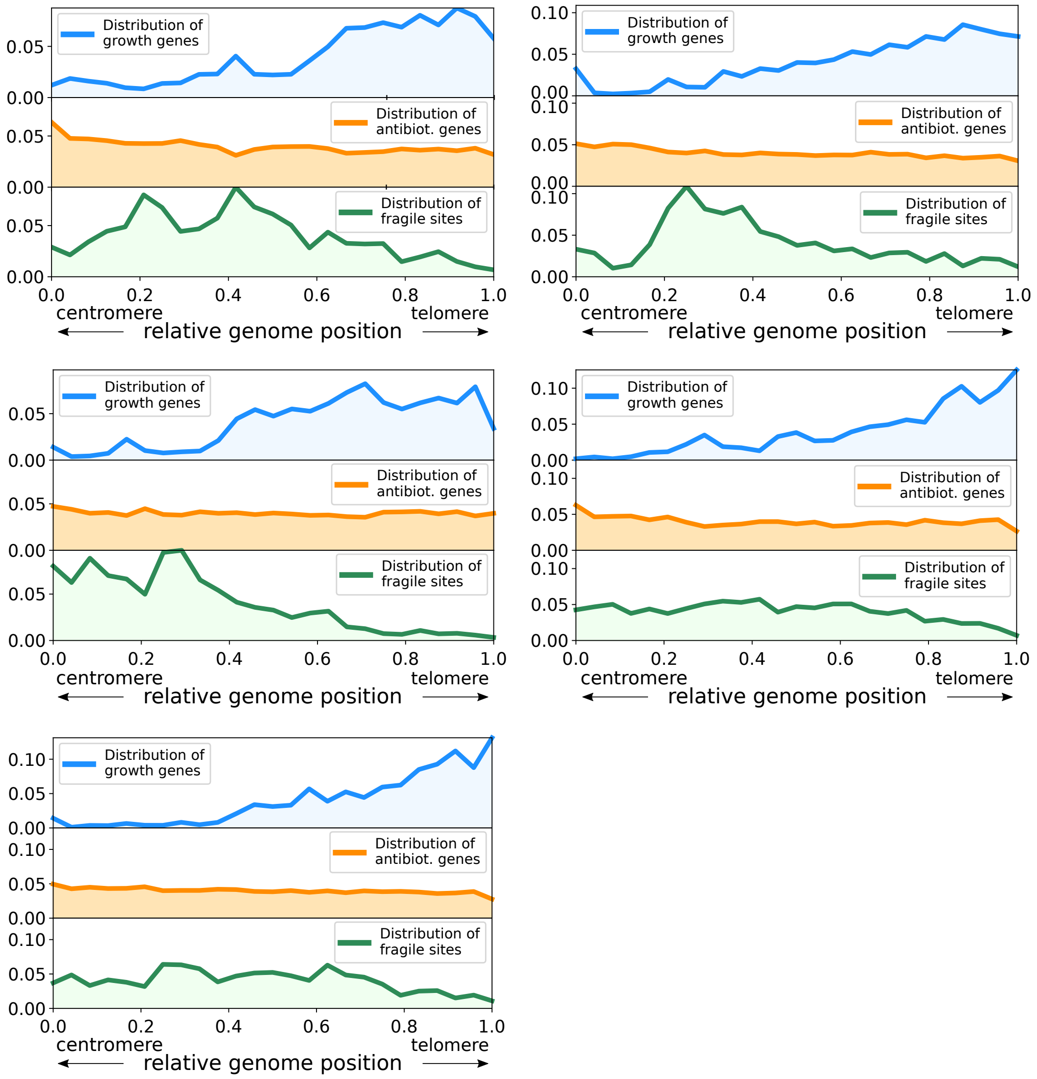

Appendix Figure S12: The evolved genome architecture is a very robust outcome of the evolutionary dynamics. Each pane corresponds to (and gathers data from the last time step of) one of the five simulations shown in Appendix Figure S1.

9 Competition between wildtype and genomes evolved with shuffling

Bacteria that evolved division of labor under default settings, always win in the competition with genomes evolved with genome shuffling (main text Fig. 4). We choose three wildtype genomes (see Supplementary Section 3 for genome sequence) and two genomes that evolved with sequence shuffling, and perform all pairwise comparisons (10 times) between the two types following the same protocol as in Supplementary Section 3. In all cases, the wildtype genome wins over the one evolved with shuffling.

The sequences of the genomes evolved with shuffling are:

```
>Shuffling Genome 1:  nr.  F=5, nr.  A=23, nr.  B=1
ABAAAAAAAAAFFAAAAAAAAAAAFFFAA

  >Shuffling Genome 2:  nr.  F=7, nr.  A=28, nr.  B=1
AFAAAAFAAAAABAAAAAFAAFFAAAAAAAAFAAFA
```

10 Evolution of genome architecture with an additional gene type

Appendix Figure S13 shows that an additional gene type, assumed to be essential for survival in at least $n_h = 10$ copies, evolves to be correctly partitioned towards the centromere of the chromosome. With this evolved genome organization, these genes do not compromise division of labor because they are placed to the 5' of fragile sites, thus ensuring survival of both a wildtype bacterium with a complete genome and of an antibiotic-producing mutant arising through fragile site deletion.

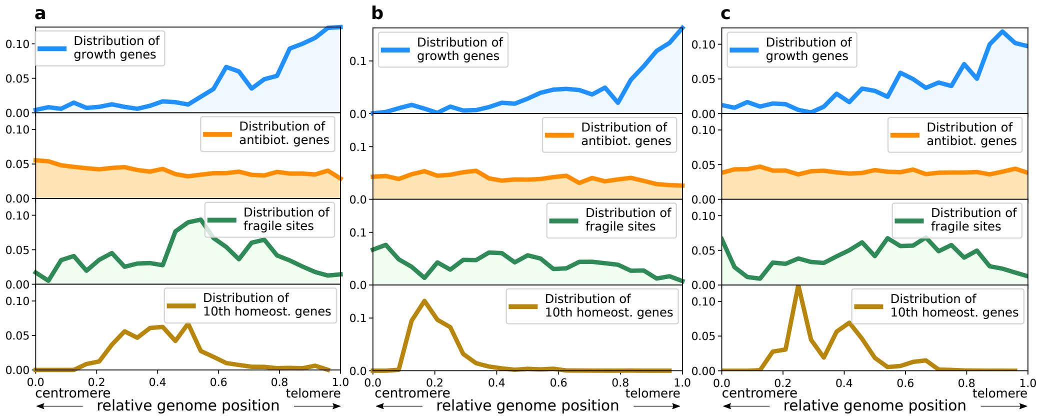

Appendix Figure S13: Spatial localization of genes in the genome, for three runs (**a**, **b**, **c**) in which an additional gene type is present. The first three rows in all pane show that normalized genomic location of growth-promoting genes, antibiotic genes and fragile sites. The last row shows the distribution of the genomic location of the 10th homeostatic gene - since at least $n_h = 10$ copies must be present for survival.

11 Division of labor evolves if colonies have sufficient time to develop and compete

At the beginning of each growth cycle, bacteria replicate locally and expand into the available space. During this initial phase, bacteria with more growth-promoting genes are at an advantage. When we reduce the duration of the growth cycle we observe that selection favors growth over antibiotic production (Appendix Figure S14, cycle duration < 1000 time steps). Genomes accumulate growth-promoting genes but no antibiotic genes - and consequently do not divide labor. When growth cycles are further reduced (≤ 500 time steps), population size does not recover between sporulation events (which sample a fraction of bacteria) and the system goes extinct. With longer growth cycles (≥ 1000), colony development progresses to the point that interference competition between colonies becomes common, because colonies come in contact with one another. This selects for antibiotic production (Appendix Figure S14), which, in turn, selects for division of labor and a genome architecture that makes this possible. In summary, inter-colony competition drives the evolution of antibiotic diversity and genome instability, and determines the condition for the emergence of division of labor.

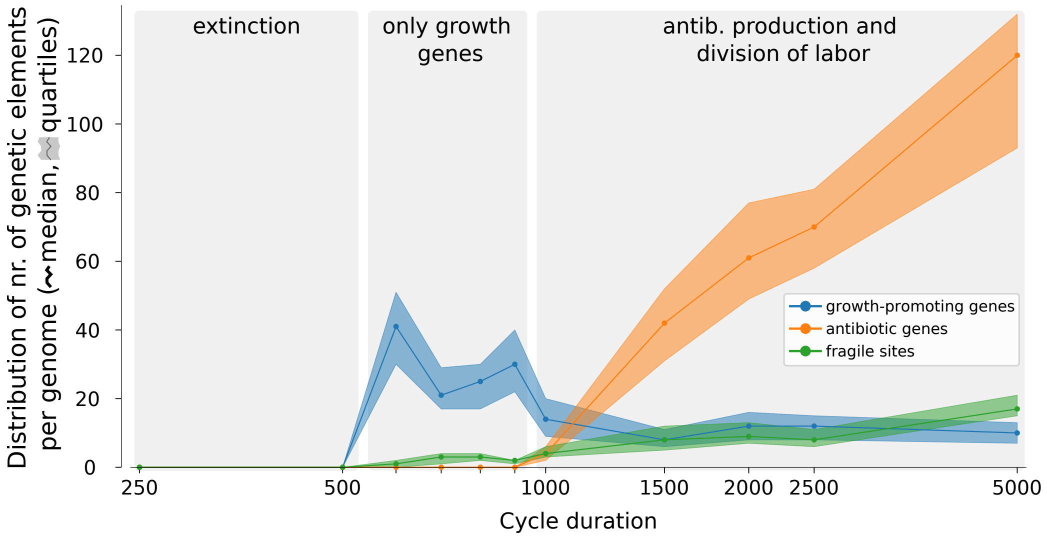

Appendix Figure S14: The genome composition depends on the duration of the growth cycle. The plot shows the distribution of growth-promoting genes (dot: median, shaded area: quartiles), antibiotic genes and fragile sites in the genomes of all individuals in a population, after long-term evolution, for different durations of the growth cycle (for cycle duration ≤ 500 the system goes to extinction).

12 The effect of destroying spatial structure

Starting from an evolved colony, we ran five simulations in which the location of bacteria was randomized at every time step. This disrupts colony formation, with two consequences: all bacteria are likely exposed to all antibiotics (in a colony this is not the case, because bacteria at its center do not come in contact with antibiotics other than those they themselves produce), and the local benefit of antibiotic production is also lost. We find that division of labor is maintained and the number of antibiotic genes increases, presumably because non-local competition always selects against loss of antibiotic resistance and favors an increase in antibiotic diversity. Appendix Figure S15 shows that this results in a steady increase in the number of antibiotic genes.

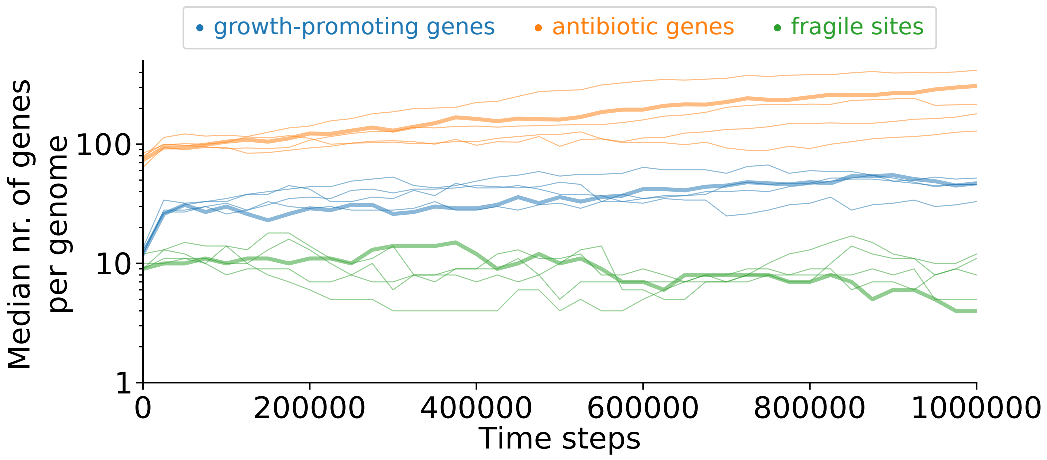

Appendix Figure S15: Starting from genomes that divide labor, evolutionary dynamics of the genomes in the population when the system is mixed every time step. The system is initialized from evolved bacteria that divide labor. Each line represents the median number of each gene type in the genome, in a simulation. The bold line emphasizes the genome dynamics in one simulation (the simulation is chosen randomly out of the five replicas, but it is the same across panes). The lattice location of each bacterium is randomized every time step. All the runs are initialized from the same evolved colony.

We also tested the effect of disrupting spatial structure on the evolution of bacteria that do not divide labor, i.e. starting from randomly generated genomes. Appendix Figure S16 shows that genomes evolve a large number of growth-promoting genes, and no antibiotic genes or fragile sites, indicating that they are solely being selected on the basis of their growth rate.

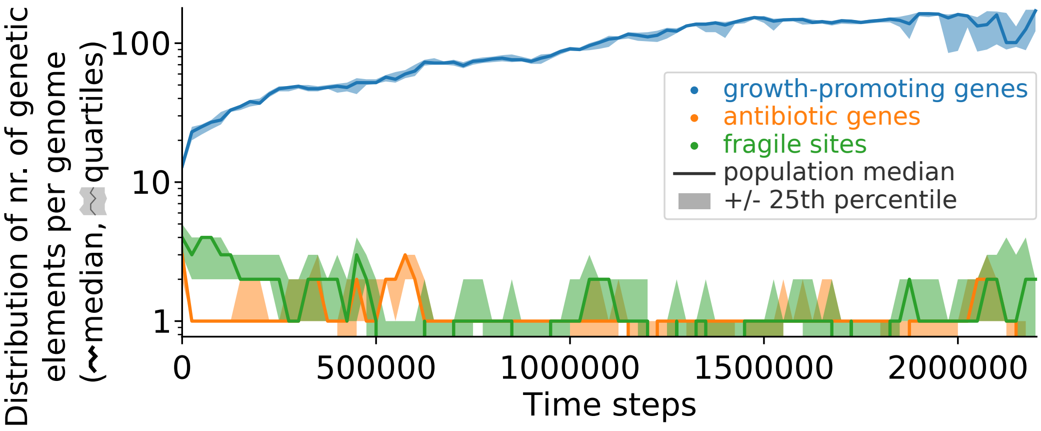

Appendix Figure S16: Starting from random genomes, evolutionary dynamics of the genomes in the population when the system is mixed every time step. The simulation is initialized from a population of random genomes of length 20, with average proportion of growth-promoting genes, antibiotic genes and fragile sites 12:4:4. The plot shows the median and quartile values of the number of each type of genetic element, calculated from all genomes in the population, at the end of every growth cycle.

13 The number of antibiotic genes is partly due to selection for diversity

We expect that selection for a diverse antibiotic repertoire in combination with genetic drift shape the number and types of antibiotic genes in the model. In this section we show that the large and diverse antibiotic repertoire is not a consequence of the model assumption that more antibiotic genes increase production rate. To do this we modified the antibiotic production rate so that it is independent of the number of antibiotic genes. In the modified system (cf. Methods), antibiotic production per unit time $k'_{\text{ab production}}$ depends solely on the number of growth promoting genes - if at least one antibiotic gene is present, and is zero otherwise:

$$k'_{\text{ab production}} = A'(a)I(g)$$

with

$$A'(a) = \begin{cases} 0 & a = 0 \\ \alpha'_a & a \geq 1 \end{cases}$$

and $I(g) = exp(-\beta_g g)$ as in the Methods. Appendix Figure S17 shows that results are robust to this change, and a large number of antibiotic genes is incorporated in the evolved genomes, indicating that selection is on antibiotic diversity rather than antibiotic number (cf. Appendix Figure S2). The large fluctuations in the number of antibiotic genes in Appendix Figure S2) suggest that drift also contributes to antibiotic diversity. We hypothesize that this might be especially the case for the antibiotic genes residing downstream of the fragile sites, which have low probability of expression in the wild-type and are deleted in the mutants. Duplications, deletions and type-changes of these genes are effectively neutral, and are therefore likely subject to drift. When some of these genes are duplicated to upstream of the fragile site cluster, they can provide a large selective advantage to the bacterium if they have diversified enough. Thus, a combination of selection and drift likely generates the observed number and diversity of antibiotic genes in the model.

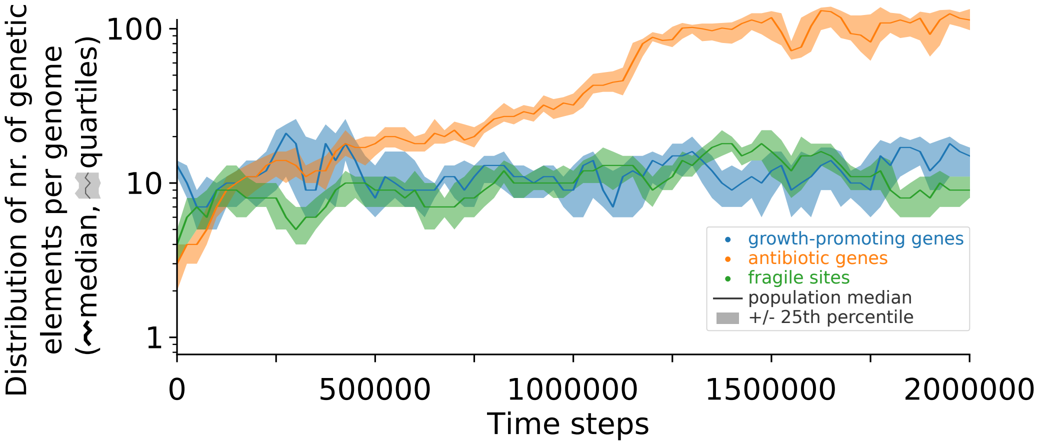

Appendix Figure S17: A large number of antibiotic genes is incorporated in the genome despite the antibiotic production rate being independent of the number of antibiotic genes. The plot shows the median and quartile values of the number of each type of genetic element, calculated from all genomes in the population, at the end of every growth cycle. All other parameters are identical to those of the simulation shown in Appendix Figure S2.

14 High and diverse antibiotic production

The large number of antibiotic genes and their variability are partly due to selection for antibiotic diversity (multi-toxicity), see Appendix Figure S2, and is not a mere consequence of selection for higher antibiotic replication rates (see 13). Moreover, the spatial dynamics shown in 2 indicate that invasion dynamics depend on antibiotic production. Here, we show that bacteria are susceptible to most (of the many) antibiotics secreted in the lattice (Appendix Figure S18). Altogether, this shows that antibiotic diversity is beneficial to the colonies.

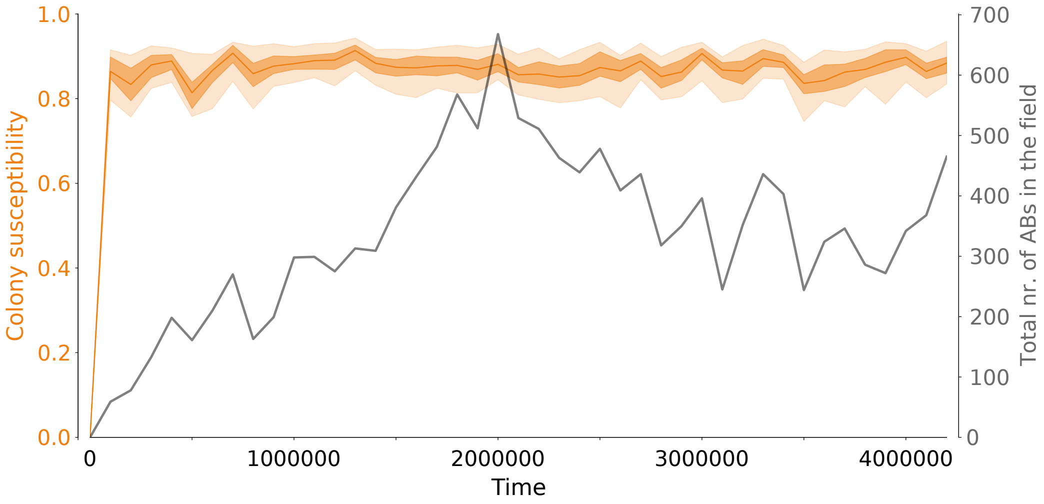

Appendix Figure S18: Evolutionary dynamics of antibiotic production and susceptibility. We extracted the total number of antibiotics produced per time step from the same run shown in Appendix Figure S2, and measured the susceptibility of each bacterium in the lattice as the fraction of antibiotics that causes a 75% fitness decrease or more. Orange line: median susceptibility, shaded areas are, top to bottom, 5th, 25th, 75th and 95th percentile. The gray line shows the total number of different antibiotics in the system.

15 The total number of possible antibiotics determines the evolution of colony susceptibility

The evolutionary potential for antibiotic diversification depends on the total number of possible antibiotics, which is determined by the length of the bitstring that defines the antibiotic (Appendix Figure S19; with a binary strings of length ν, the volume of the antibiotic space is 2^{ν}). With long antibiotic strings ($\nu \geq 8$), bacteria occupy a small part of the total antibiotic space, and bacteria are susceptible to most antibiotics produced by other colonies (Appendix Figure S19 red line, see also Suppl. Section 14). This indicates that large antibiotic diversity promotes competition - an eco-evolutionary outcome previously named "multi-toxicity" in the context of colicin evolution models [54]). When the evolutionary potential for antibiotic diversification is small ($\nu \leq 6$), the system reaches a different eco-evolutionary steady state - characterized by low susceptibility because each genome contains many copies of each possible antibiotic gene. This state has been previously called "hyper-immunity" and persists because the loss of resistance to any antibiotic leads to extinction.

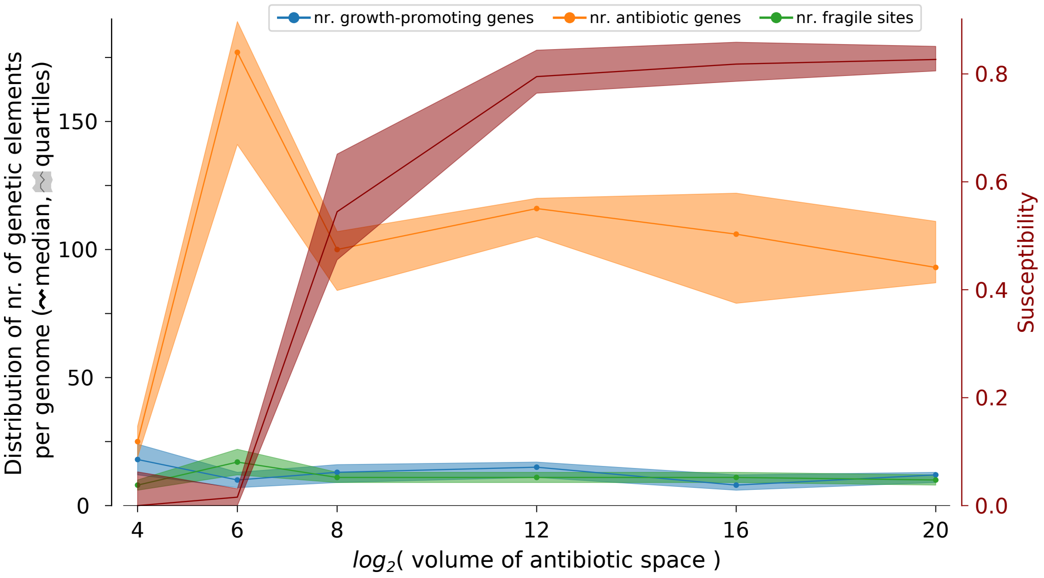

Appendix Figure S19: The total number of possible antibiotics, i.e. the volume of the antibiotic space, determines steady-state genome size and colony immunity. We ran one simulation for each antibiotic bitstring length $\nu \in 4, 6, 8, 12, 16, 20$. The volume of the antibiotic space is 2^{ν}. The plot shows the distribution of growth-promoting genes (dot: median, shaded area: quartiles), antibiotic genes, fragile sites in the genomes of all individuals in a population, as well as the distribution of colony susceptibility (defined as the fraction of all antibiotics in the lattice to which the colony is susceptible), after long-term evolution.

16 The architecture of genomes evolved when antibiotic volume space is small

We tested how the model behaves when we decrease the size of the antibiotic bitstring ν. This has the effect of reducing the number of possible antibiotics available to bacteria.

We ran a simulation where the size of the antibiotic bitstring was $\nu = 6$. After long-term evolution we measured the genome architecture in the population in the same way as shown in Suppl. Section 8. Appendix Figure S20 shows that the volume of the antibiotic space does not affect the evolution of the architecture that supports division of labor.

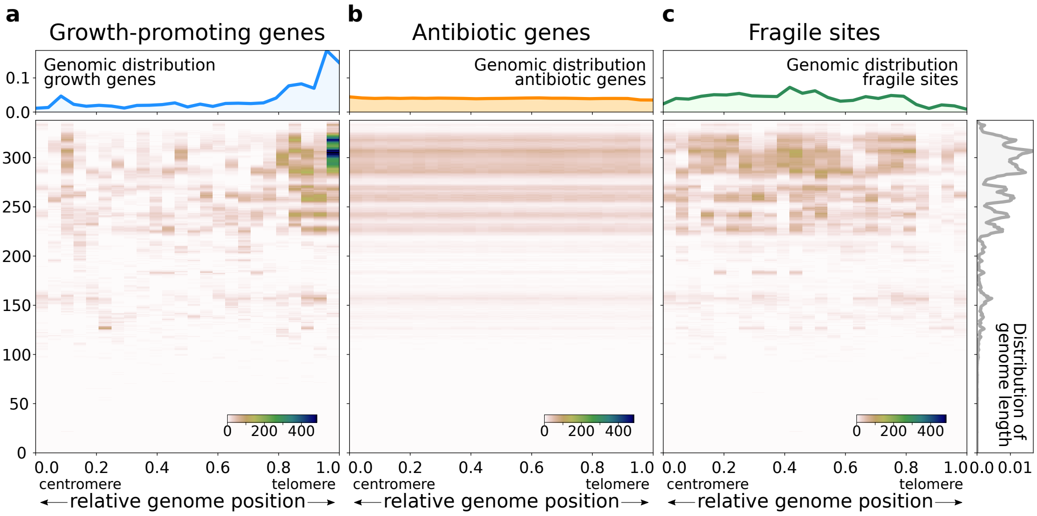

Appendix Figure S20: Small antibiotic space ($2^6 = 64$ different antibiotics) volume does not affect division of labor. We ran a simulation identical to that shown in Appendix Figure S2, except for an antibiotic bitstring length of $\nu = 6$.

We further tested whether division of labor would evolve when the total number of possible antibiotics was very small, by running a simulation with antibiotic bitstrings of size $\nu = 2$. With this bitstring size, the number of possible antibiotics is 4. No division of labor evolved in the simulation (Appendix Figure S21). This is likely due to bacteria requiring few genes to obtain full resistance to antibiotics.

Moreover, each antibiotic gene gives some protection against the other antibiotics (for instance, the antibiotic bitstring "10" gives some resistance to "00" and "11", because their distance is 1), and at most two mutations are sufficient to convert any antibiotic type into any other. This results in fast evolution of resistance and diminished evolutionary pressure from secreting antibiotics (for both defense and offense). Because of this, colonies have less incentive to divide labor, and instead cells increase their growth rate, minimizing the number of fragile sites in the process.

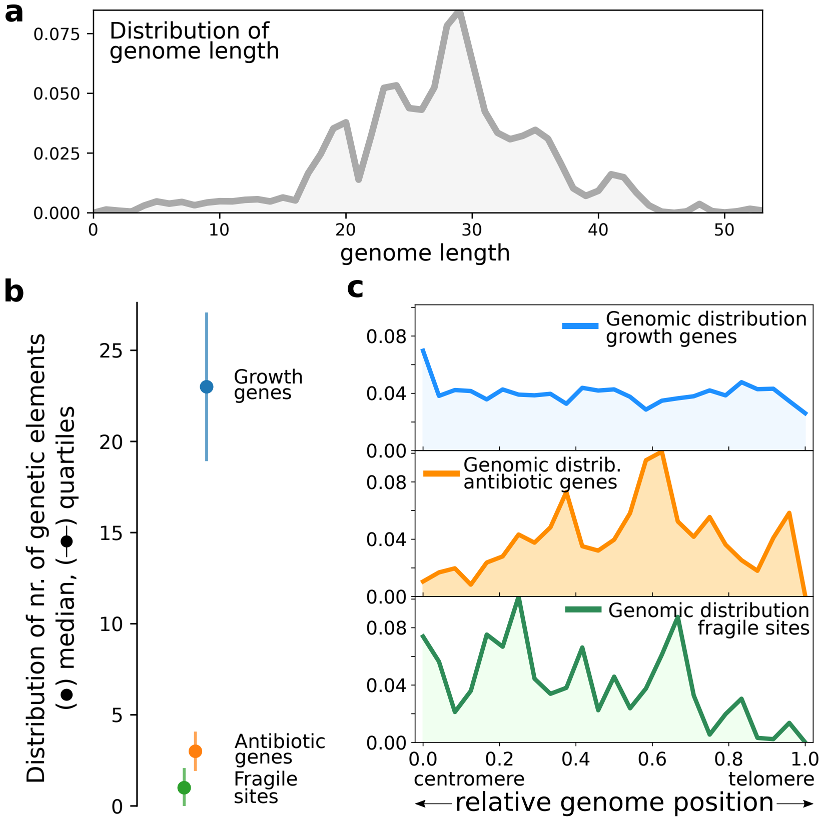

Appendix Figure S21: No division of labor evolves when the antibiotic space is very small ($2^2 = 4$ different antibiotics). We ran a simulation identical to that shown in Appendix Figure S2, except for an antibiotic bitstring length of $\nu = 2$. **a** Distribution of genome size for the whole population at one time point, after long-term evolution. **b** Distribution of number of genetic elements. **c** Genomic localization of genetic elements.

17 Division of labor evolves when the deposition zone of antibiotics is smaller and when the resistance to antibiotics is broader

Smaller deposition zone of antibiotics We ran a series of simulations in which we varied the radius r_a of the circle in which antibiotics are deposited by bacteria. Bacteria benefit less from antibiotics when the r_a is smaller, because an antibiotic producing bacterium protects fewer colony members. Therefore, we expect that for small r_a division of labor does not evolve, as it does not confer enough advantage. We ran two simulations for each of the following values of the radius: $r_a \in \{1, 2, 3, 5, 8\}$, and we included the results of the five simulations shown in Suppl. Suppl. Section 1 which have default radius $r_a = 10$. These values for the radius correspond to a protected area $A(r_a) \in \{5, 13, 29, 81, 197, 317\}$ lattice sites. Appendix Figure S22 shows that antibiotic genes, fragile sites and growth genes are accumulated in genomes that divide labor when $r_a \geq 3$ (although for $r_a = 3$ this happens only in one of the two simulations). This shows that division of labor is very robust to varying r_a. Bacteria only maximize growth rate for smaller values of r_a antibiotics, and neither fragile sites nor antibiotic genes are accumulated in the genome.

Broader resistance to antibiotics Both antibiotics and antibiotic genes are characterized by a bitstring, which determines their type. Antibiotic resistance in the model is determined by matching these two strings. A larger difference between the two strings results in a lower resistance of the bacterium, according to the function $R = e^{-\beta_r S^2}$ (see Methods). A higher value of β_r results in a lower resistance even for small mismatches, whereas a smaller β_r makes resistance more broad. We ran a series of simulations in which we varied β_r (Appendix Figure S23). We note that the value of R is dominated by the quadratic sum (S^2) when S becomes large. Therefore, we vary β_r over a very large parameter range to compensate for large S. Division of labor evolves when $\beta_r \geq 0.0003$, indicating that the system is robust to decreasing benefits of antibiotic production (for comparison, the value used for main text results is $\beta_r = 0.3$). For very small β_r, any antibiotic gene

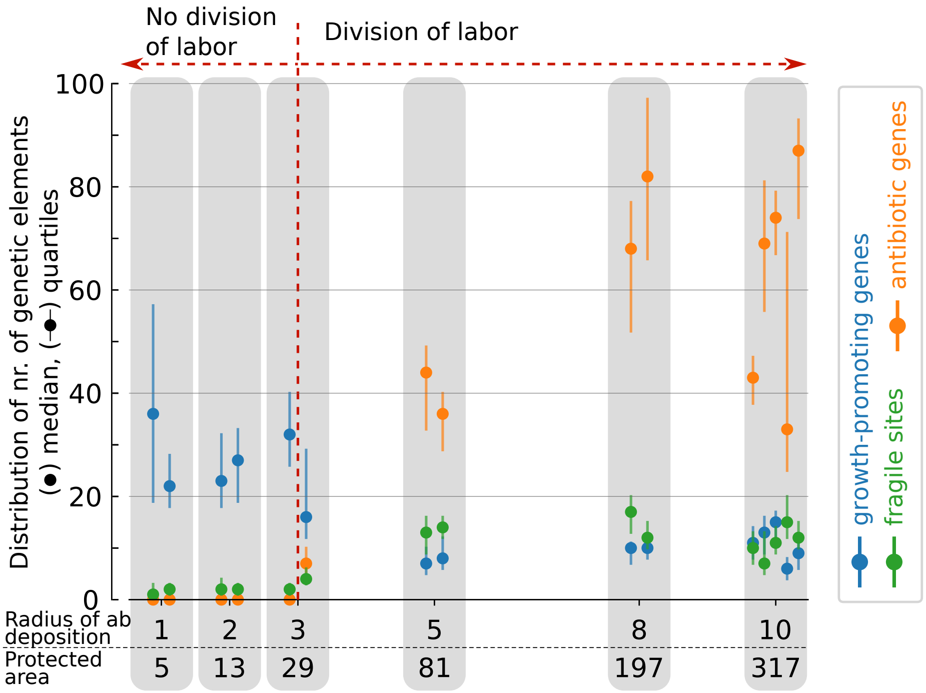

Appendix Figure S22: Evolutionary outcome of changing the radius of antibiotic deposition. Two simulations per radius length are run and the distribution of the nr. of genetic elements is reported, except for radius = 10, for which the five runs from Suppl. Section 1 are used. The area in nr. of lattice sites corresponding to each deposition radius is also shown. Data collected after long term evolution, at the end of a growth cycle. All other parameters and initialization are the same as in Appendix Figure S1.

provides resistance to any antibiotic, and thus there is no benefit from secreting antibiotics. The system does not evolve division of labor in this case, and instead maximizes growth.

We note that the extreme advantage provided by antibiotics in the model may partly be due to the specific way they are modeled, e.g. that they are either present or absent, and there is no intermediate concentration. A mildly deleterious antibiotic in low concentration might not have any effect on a susceptible bacterium. Thus, a continuous diffusion model could quantitatively change these results, by shifting the values of β_r at which division of labor evolves. However, we expect that results will remain qualitatively similar, because antibiotic production is strongly selected for – as it allows colonies to further invade the lattice. This more precise model of antibiotic production, diffusion and killing was not included in the model to limit the computational load (see Discussion).

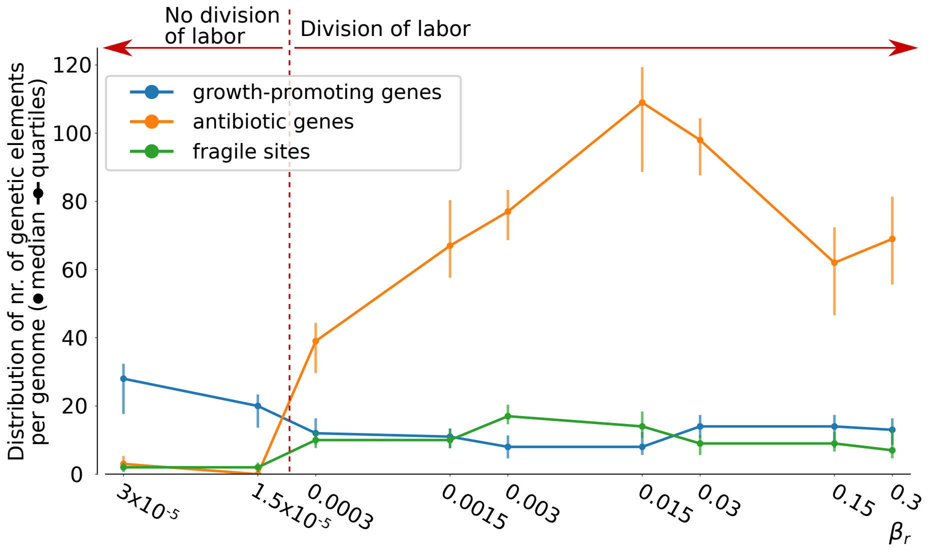

Appendix Figure S23: Evolutionary outcome of changing the antibiotic resistance factor β_r. One simulation for each value is run, and the distribution of the nr. of genetic elements is reported. $\beta_r = 0.3$ is the value used for the simulations presented in main text. Division of labor can be inferred from the increase in number of antibiotic genes and fragile sites for $\beta_r \geq 0.0003$. Data collected after long term evolution, at the end of a growth cycle. All other parameters and initialization are the same as in Appendix Figure S1.

18 Mutation-driven division of labor evolves over a wide range of (per-fragile sites) mutation rates.

In main text Fig. 5 we show that division of labor evolves over a wide range of fragile sites mutation rates. The main text figure indicates division of labor by the distance between the median nr. of growth-promoting genes in the genome of antibiotic producing bacteria and replicating ones. In Appendix Figure S24, we show the data used to obtain main text Fig. 5, i.e. the distribution of antibiotic producing and replicating bacteria.

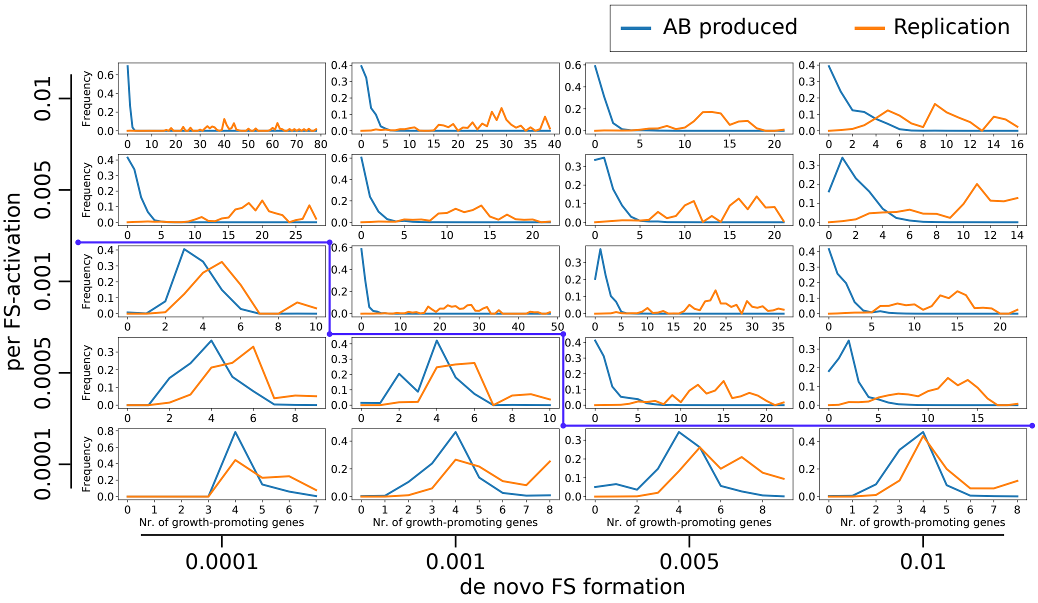

Appendix Figure S24: Division of labor evolves over a wide range of fragile site mutation rates (i.e. de novo fragile site (FS) formation μ_n and per FS-activation μ_r). Each plot shows the frequency of antibiotic producers (blue) and replicating bacteria (orange) as a function of the nr. of growth-promoting genes in their genome, for a different combination of de novo fragile site (FS) formation μ_n and per FS-activation μ_r. A larger difference between the two curves indicates division of labor, because the two tasks are carried by genetic distinct individuals in the same colony. Division of labor evolves for the parameter combinations above the blue line. One simulation is run with the parameter combination indicated in the figure (all other parameters are identical to those in the caption of 1). Data is collected from the entire population, for one growth cycle after long-term evolution (in all cases after at least 600 growth cycles).

19 The genome composition of populations that evolve division of labor over a wide range of (per-fragile site) mutation rates.

Appendix Figure S25 shows the genome composition in the evolved population for different values of fragile site mutation rates μ_f and μ_n. A larger number of growth-promoting genes corresponds to division of labor because these genotypes cannot produce a lot of antibiotics, which are instead overproduced by other members of the colony (which arise through mutations). When division of labor evolves, we observe a larger number of fragile sites at evolutionary steady when the per-fragile site deletion rate μ_f is smaller, suggesting that there is selection for a target mutation rate. The same data is used to generate both this figure and Appendix Figure S24.

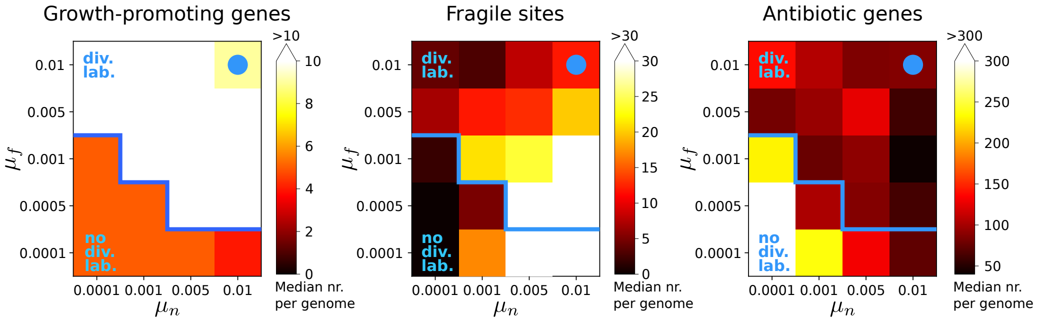

Appendix Figure S25: Quantitative changes in genome composition when fragile site mutation rates are changed (de novo fragile site (FS) formation μ_n, and per FS-activation μ_r). Notably, when division of labor evolves, a larger number of fragile sites compensate for smaller per-fragile site deletion rate μ_f. Each plot shows the median number of genetic elements - growth-promoting genes, fragile sites and antibiotic genes - per genome in a population evolved with the indicated fragile site mutation rates (the same data is used to generate Appendix Figure S24). The blue dot (top right corner of each heatmap) indicates default values used in the rest of the manuscript. The blue line indicates the approximate location of the boundary for the evolutionary phase transition between division of labor (above the line), and generalist (below).

20 Division of labor persists in evolved genomes when de-novo fragile site formation is set to zero

Division of labor persists in evolved genomes, when de-novo fragile site formation μ_n is set to zero. We initialized six simulations, each with one of three evolved genomes (two simulations with each initial genome). Appendix Figure S26 shows genome composition after 500×10^3 to 680×10^3 time steps (200 to 272 growth cycles). The number of fragile sites is small but maintained above zero, and the number of both growth-promoting genes and antibiotic genes is large, indicating that division of labor occurs to enable both cell division and antibiotic production. Moreover, the evolved genome architecture also persists.

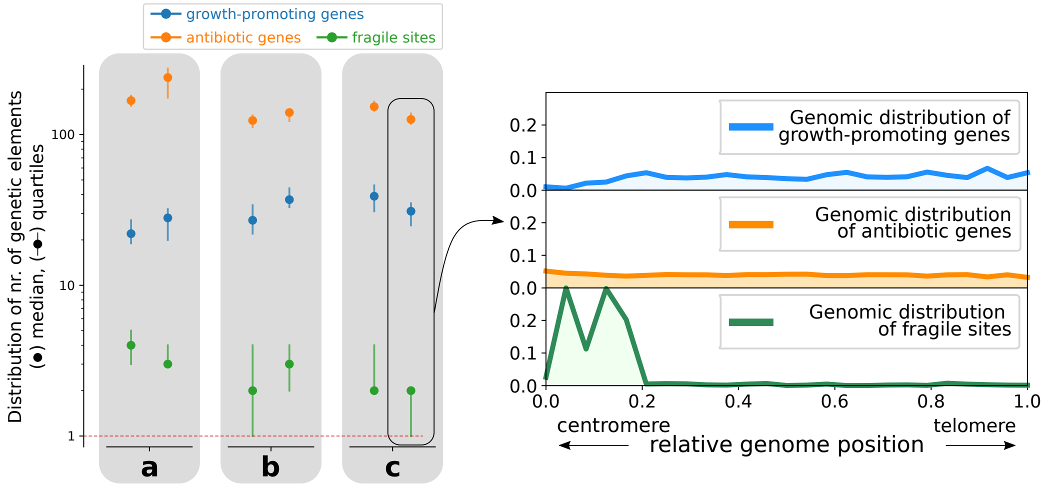

Appendix Figure S26: Mutation-driven division of labor persists in evolved genomes, when de-novo fragile site formation μ_n is set to zero. Left: steady-state genome composition after long-term evolution for six simulations started from three independently evolved genomes (**a**,**b**,**c**). Right: genome architecture extracted from the whole population at the last time point of one simulation. The plot shows that the evolved genomes are organized so that fragile sites are at the 5' of growth genes, and thus can generate antibiotic-producing mutants.
