# Supplementary material for: Evolution of genome fragility enables microbial division of labor
Source: Mol Syst Biol. 2023 Feb 2;19(3):e11353. doi: 10.15252/msb.202211353 (PMC9996244; doi:10.15252/msb.202211353)
Supplement: Supplementary file 2 — Movie EV1 [file MSB-19-e11353-s002.zip › Movie EV1 legend.docx]

Movie EV1: Movie of the eco-evolutionary dynamics during a growth cycle. Different colors represent colonies arising from single spores, darker shades of yellow indicate that more antibiotics are present. Black is background (empty lattice sites).
