# Supplementary material for: Evolution of genome fragility enables microbial division of labor
Source: Mol Syst Biol. 2023 Feb 2;19(3):e11353. doi: 10.15252/msb.202211353 (PMC9996244; doi:10.15252/msb.202211353)
Supplement: Supplementary file 3 — Movie EV2 [file MSB-19-e11353-s003.zip › Movie EV2 legend.docx]

Movie EV2: Movie showing genome composition in bacterial colonies over one growth cycle. Darker shades of blue indicates larger number of antibiotic genes in non-producing (and weakly producing) cells, red dots indicate antibiotic-producing bacteria (brighter red corresponds to higher production), gray corresponds to antibiotics. Black is background (empty lattice sites).
