# Supplementary material for: Evolution of genome fragility enables microbial division of labor
Source: Mol Syst Biol. 2023 Feb 2;19(3):e11353. doi: 10.15252/msb.202211353 (PMC9996244; doi:10.15252/msb.202211353)
Supplement: Supplementary file 4 [file MSB-19-e11353-s004.pdf]

Evolution of genome fragility enables microbial division of labor

Enrico Sandro Colizzi, Bram van Dijk, Roeland Merks, Daniel Rozen, and Renske Vroomans
DOI: 10.15252/msb.202211353

Corresponding author(s): Enrico Sandro Colizzi (enricosandro.colizzi@slcu.cam.ac.uk)

Review Timeline:

Transfer from Review Commons:	14th Sep 22
Editorial Decision:	15th Sep 22
Revision Received:	3rd Nov 22
Editorial Decision:	7th Dec 22
Revision Received:	17th Jan 23
Accepted:	19th Jan 23

Editor: Jingyi Hou

Transaction Report: This manuscript was transferred to Molecular Systems Biology following peer review at Review Commons.

Review #1

1. Evidence, reproducibility and clarity:

Evidence, reproducibility and clarity (Required)

This study proposes (and uses) an elegant model of bacteria evolution to study how division of labor can emerge through the interaction between non-random mutations (occurring at some specific ``fragile' genomic sites) and genome architecture. The study is very interesting and the results are convincing. My main concerns are about the presentation of the model and results. Although I am confident about the results, some elements should be clarified for a better understanding and for a correct interpretation of the results. Two points in particular (detailed below as major comments) require clarification.

Major comments:

- the notion of telomere/centromere is used all throughout the paper but I think it is used in a misleading way. First, it seems that here there is only one telomere (but this is actually a detail of the model). More importantly, as long as I know, it is well known that in S. coelicolor the sequence degenerates more rapidly when getting closer to the telomeres (but telomeres are defined independently from this property). But here, the notion of telomere is precisely directly determined by its mutational instability (respectively, the centromere is defined by its stability). Although this is reasonable given the objective of the model, it forbid the use of sentences like "we observed that the genome of the evolved colony founded had two distinct regions: a telomeric [...] and a centromeric [...]" (line 234) or "When bacteria divide, mutations induced at fragile sites lead to the deletion of the part of the genome distal to them, causing large telometic deletions" (line 239 - this is not a result but a hidden description of the model) as this distinction between the two regions is not an outcome of the simulation but rather given a priori as a coded property of the fragile sites that all lead to deletions on the same -- called telomeric -- side (of course, formally if the genome contains no fragile site, there is no distinction but still). Please clarify this in the main text and in the methods.
- In most part of the paper (methods, results, figures, sup mat...) antibiotics are considered to have a concentration (or a high/low production) but at least twice in the text (lines 165 and 488) it is said that only the presence/absence of antibiotics is modelled. I was not able to understand how the continuous values are transformed into presence/absence (is there a threshold?) but more importantly, I strongly suspect that this choice has a strong influence on the outcome. For instance, with a diffusion

radius equals to 10, it means that an antibiotics producing cell is able to protect 2*\pi*10=~60 replicating cells. Hence, one could conjecture that the fraction of antibiotic-producing mutants should a little more than 2%... which is what is observed by the authors. So (1) please clarify this point (2) discuss (or experiments) the consequences of this choice on the conclusion.

Minor comments:

- line 262: "We conclude that genome architecture is a key prerequisit for the maintenance of mutation-driven division of labor". Given the model hypotheses you cannot be so affirmative (it is a key prerequisit... in this model!)
- line 286: "cannot" is probably too strong. It has not been observed...
- line 288 and following: you seem to consider that there is "selection for diversity". Given the large number of possible antibiotics and given that cells are "automatically" resistant to the antibiotics they produce, could it be simply drift? There is a clear selection pressure to limit the number of growth-promoting genes but no such pressure exist for antibiotics. Hence their number could simply drift (note that figs 2 and SF1 both use a log scale; random variations due to drift could be hidden by the log. Fig. SF2 does use a log scale and shows a dynamics that---to my eyes---claims for drift rather than for selection of diversity).
- line 340: "ends" should be "end" when discussing the model
- line 345: "a telomeric region" should be "telomeric regions" when discussing the bacteria
- line 359: "S. ambofaciens" should be italic
- line 365: same for "Streptomyces"
- line 245 states that colonies begin clonally but methods (lines 434-438) don't support this. Colonies don't begin clonally but they begin without antibiotic-producing spores (see also line 618)
- line 442: "their" should be "its"
- line 446: "hotspot for recombination" no, for "deletion"
- line 449: please remove brackets around the reference.
- line 458: if I understood it correctly, there is no explicit competition in the model. Competition simply comes from the asynchronous replication. Am I true? Could you clarify that point?
- line 490: "the antibiotic deposited is chosen randomly and uniformly among them". This is not fully clear. I suppose the bacteria is still resistant to all the antibiotics it \it{can} produce?
- figure SF1: please use the same scales as in figure 2 such that the two plots can be easily compared
- section S3 and figure SF4: What is to be understood from the figure is not clear to me. Seems that WTs win only if generalists produce less AB or replicate slower (?) Is

it true?
- I found it very difficult to draw conclusion from section S4, S5 and S6. These experiments should be analyzed with the help of mathematical analyses of the equations. Moreover, the understanding of these results are rendered difficult due to the lack of clarity regarding the discrete (or not) nature of the antibiotic production/action/diffusion
- S7 and fig SF9. It is unclear to me why the fraction of mutants decrease along time elapsed in the cycle. Please explain.
- Figure SF14: what are the tin lines? if they correspond to the five repeats, how can it be that the bold line be the median?
- S13 and figure SF15: given that AB concentration is ON/OFF, is this result really surprising? This also questions about the accumulation of AB genes in the original model. Although the authors regularly claim that this is due to selection for diversity, drift could also be at play (see above)
- S17: for radius 1, 2 and 3, the aliasing is likely to be strong. Hence, the results cannot be interpreted with this sole information. Please give e.g. how many cells are "protected" for each radius (e.g. for r_{alpha}=1, this value can vary between 1 and 9!)
- L742: "matching the antibiotic bitstring with the bitstring of the antibiotic". True and actually elegant but simpler formulation could ease the reading...
- lines 746-751 and figure SF21: There again, could it be a consequence of the AB ON/OFF diffusion model?
- S18-S19-S20: what should the reader understand from these results? Please better comment the figures.

Referees cross-commenting

Sorry about the confusion about the computation of the number of cells protected by a single AB-producing cell. Of course it is of the order 10*\pi^2 !!! The global argument still holds but the number of cells protected is of course larger than 60 (note that, due to aliasing at the periphery the exact number of cells in the protected area is difficult to determine).

2. Significance:

Significance (Required)

First, an very importantly, I must say that I am no familiar with the biological model (Streptomyces coelicolor). So I am not fully able to judge the biological significance of this research (i.e. whether the way division of labor is achieved here enlights---or not---the biology of this bacteria). However, on the computational side, the model and

the results (as they are summarized in the conclusion) are very interesting on their own and deserve publication.

Remark: a lots of supplementary results are added to the paper that are not not fully explained or analysed. Please, better discuss all these results and their significance.

3. How much time do you estimate the authors will need to complete the suggested revisions:

Estimated time to Complete Revisions (Required)

(Decision Recommendation)

Less than 1 month

4. Review Commons values the work of reviewers and encourages them to get credit for their work. Select 'Yes' below to register your reviewing activity at Publons; note that the content of your review will not be visible on Publons.

Reviewer Publons

Yes

Review #2

1. Evidence, reproducibility and clarity:

Evidence, reproducibility and clarity (Required)

The manuscript "Evolution of genome fragility enables microbial division of labor" presents a model of genetically-based division of labour in bacterial colonies. It is postulated that two essential processes, growth and the important for elimination of competitors production of antibiotics, are poorly compatible in a single cell. The beneficial for a colony cell specialization is assumed to be determined only

by genetic differences that appear via deletions of growth- promoting loci. These deletions and production of various antibiotics are mediated by a rather elaborate genetic architecture, which includes position-sensitive "fragile" sites, mutable antibiotic and growth-promoting genes. The model produces rather predictable results that under sufficiently strong incompatibility between growth and antibiotic production, the long-term evolution results in formation of mosaic of colonies, each specialized in production of its specific set of antibiotics. Such production is facilitated by evolving rapidly mutable genomes that constantly generate non-reproducing antibiotic-pumping cells.

The model appears very thoroughly developed and analyzed, and all major conclusion are intuitively appealing. Overall, the manuscript reads as a well-written quantitative proof of the principle of genetically-based division of labour between bacterial cells. The only part of the model that I'm a bit sceptical about is the unwarranted complexity of the genetic architecture. Unless the introduction of "fragile" sites and the directional ordering of genes is strongly justified by empirical data, a simpler and more clear assumption about mutational incapacitation of growth genes would suffice to reproduce the predicted phenomenology. So adding such empirical evidence would boost the relevance of the genetical part of the model. In the present form, all observed adaptations are inevitable simply because the expected division of labour will not evolve without each of them due to the design of the model.

A couple of minor comments...

217 This is achieved when fewer growth-promoting genes are required to inhibit antibiotic
218 production (i.e. lower βg).
Shouldn't it be "larger \beta_g"?

Whether in the main text or Supplementary materials, it woud help to add a complete population dynamics equation with all gain and loss terms.

Strikingly, we find the opposite: division of labor evolves when
224 bacteria produce fewer overall antibiotics (lower αa), under shallow trade-off conditions
225 ($hg\beta g = 5$; see Suppl. Section S6).

I don't see why it is"striking". It seems perfectly explicable that a smaller \alpha requires more dedication to antibiotic production, thus favouring specialization.

2. Significance:

Significance (Required)

Due to my relative lack of familiarity with the literature on evolution of genetically-based division of labour, I would rather not comment on the degree of innovation of the manuscript.

The text is well written and is accessible to a wide readership, so it could be recommended to a general biological or evolutionary journal.

3. How much time do you estimate the authors will need to complete the suggested revisions:

Estimated time to Complete Revisions (Required)

(Decision Recommendation)

Less than 1 month

4. Review Commons values the work of reviewers and encourages them to get credit for their work. Select 'Yes' below to register your reviewing activity at Publons; note that the content of your review will not be visible on Publons.

Reviewer Publons

Yes

Review #3

1. Evidence, reproducibility and clarity:

Evidence, reproducibility and clarity (Required)

Summary:

In this manuscript the authors explore the co-evolution of genomic architecture and division of labour in antibiotic production, in a model inspired by the bacterium Streptomyces coelicolor. In the model a genetic trade-off is implemented where the having a large number of growths promoting genes (and thus fast growth) leads to a low production of antibiotics. On the other hand, having fewer growth promoting genes allows for a higher production of antibiotics. This trade-off selects for a division of labour, where one sub population specializes in antibiotic production and another sub population specializes in reproduction. This division of labour is achieved by evolving the genome structure, so that growth promoting genes are clustered together, separated from the rest of the genome by several fragile sites (sites that allow for large deletions). This allows a single mutational event to delete a large number of growth-promoting genes, which creates a cell, lacking growth genes and that thus has a high antibiotic production (cell specializing in antibiotic production). In other words, the genome structure evolves to shape evolvability, so as to allow cells with a high growth rate to rapidly and repeatably evolve/mutate into cells with a high antibiotic production. This creates a division of labour where a part of the population specializes in growth/reproduction and another part specializes in antibiotics production. This model provides a tangible mechanism to explain a similar division of labour observed in S. coelicolor. This mechanism also fits well with the large deletions observed in antibiotic-hyperproducing S. coelicolor cells, which are also repeatably generated during colony growth.

Major comments:

- Line 69, It would be good to give a bit more information here on the (number of) different types of antibiotics produced by S. coelicolor, to help the reader understand some of the modelling choices later on, such as allowing for the evolution of a large number (16 or higher if I understand correctly) of different antibiotics and a cell automatically being resistant to all antibiotics it produces (instead of having separate resistance genes).
- Lines 127-129 It is mentioned here fragile sites in the genome might represent transposable elements or long inverted repeats. Would both of these types of fragile sites behave the same? Has it been shown that both transposable elements and long inverted repeats can lead to large deletions from a linear chromosome? It would be nice to have a bit more background on how fragile sites might work or what they might look like in an empirical context. I am a bit unsure on this, but depending on their exact empirical nature, should fragile sites not also lead to increased rates of gene duplication near themselves?
- Line 160 As alluded to before, given the introduction provided, two assumptions

come about here (lines 160-166) that lack a bit of justification/background/context. First, why does one allow the evolution of such a relatively large number of antibiotics? A bit more empirical in the introduction background would go a long way to making this assumption seem more justified. As far as I can see the genomic architecture leading to division of labour is only demonstrated for values of v that are 6 (i.e. 64 antibiotics) or above. Perhaps it is because I lack empirical background here, but this still seems to be a relatively large antibiotic space. Does the model also work with v=2? Perhaps it would be good to show a simulation with v=2 in supplementary material S16 as well.
- Line 166 The assumption is made that if a bacterium produces a certain antibiotic, it is automatically resistant to this antibiotic. Now it could be that this assumption is empirically rooted, in which case it would be good to allude to this empirical justification. I wonder how would the results be impacted if the resistance genes were separated from the antibiotic production genes? (I do not think additional simulations are in any way necessary on this point, but some more context/thoughts on this matter would be helpful, perhaps near lines 306-309)
- Figure 1 In the subscript it becomes evident that the probability of large deletions due to fragile sites is much higher (10 fold) than single gene duplications, it seems to me this should be the other way around, single gene duplications and deletions could be much more probable than fragile site induced large deletions. Would the model still produce the same results if the values for mu-d and mu-f were switched around? (Again, I do not think additional simulations are per se required, some justification for this assumption would already be plenty).

Minor comments:

- Line 36, perhaps replace "must" with " can" as there are other ways to achieve a division of labour that do not hinge on genomic architecture such as those listed in the next sentence. This sentence seems at odds with the next one, which lists ways to achieve cell differentiation that do not per se completely rely on genomic architecture such as gene regulation. Maybe consider moving this sentence to be on line 40 (after "...organized at the genome level remains unclear")
- Line 48, perhaps remove "disposable" as there is no particular reason the somatic tissue is disposable, furthermore it invokes the disposable soma theory of aging which is not relevant here
- Line 147-148 Why these particular relationships, as a reader I do not understand how these functions were constructed and how they might influence the results, a bit more justification might be helpful. Perhaps later on (results/discussion) also address what might happen if you were to use different functions?

- I am clearly biased on this matter, since I work on evolvability. So, the authors should feel free to ignore this comment. Regardless, I think the authors have shown a wonderful example of the evolution of evolvability. Perhaps it would be nice to add a little bit of an evolvability angle in the discussion. In particularl thinking about how fragile sites shape evolvability.
- Lines 404-411 It is great to see that the authors consider the wider applicability of their findings. It would be nice to add something here about the broader applicability in bacteria. As a large number of bacteria have circular chromosomes, how would these findings be impacted if circular chromosomes were at play? (I suspect they would largely still work in the same way, but keen to hear what the authors think). Referring to the work of Yona et al. 2012 on transient chromosomal duplications in yeast due to heat stress might also be good here, to show the more general applicability of the authors findings, this is another example where genomic architecture shapes evolvability.
Yona AH, Manor YS, Herbst RH, Romano GH, Mitchell A, Kupiec M, Pilpel Y, Dahan O. Chromosomal duplication is a transient evolutionary solution to stress. Proc Natl Acad Sci U S A. 2012 Dec 18;109(51):21010-5. doi: 10.1073/pnas.1211150109. Epub 2012 Nov 29. PMID: 23197825; PMCID: PMC3529009.
- Lines 412 -419 I agree with the authors that in practice the cells specializing in antibiotic production look somewhat like soma, however I would consider not using this term here as strictly speaking the antibiotics producing cells can still reproduce (be it at an extremely low rate, which leads to their loss).
- Lines 434-438 If I understand correctly authors did not explicitly model the sporulation process (instead selecting random cells from the end of a cycle). I think this is a very good modelling choice that should not be changed; however, I do wonder how the results would be affected if sporulation was more explicitly modelled (for example by adding genes for sporulation, creating a 3 way trade-off between growth, sporulation and antibiotic production). Perhaps something that could be mentioned in the discussion.

I hope this review is of some use and helps the improvement of this manuscript.

Yours sincerely,

Timo van Eldijk

2. Significance:

Significance (Required)

This study provides a clear conceptual advance by showing and studying how genome structure can evolve to create a division of labor. Thereby mechanistically explaining the division of labor in antibiotic production observed in S. coelicolor. It seems evident to me that whilst this study mainly focuses on S. coelicolor, the mechanism likely plays an important role in microbial evolution in general. Though others have previously theoretically explored such mechanisms, this study provides the first exploration modelled closely after an empirical system and hence provides a significant advance. In a more general sense, the evolution of genome architecture likely governs evolvability not just in microbes but in all life on earth. Therefore, I believe that this paper would be interesting for a general audience interested evolution. It would be of particular interest to those studying microbial evolution. My expertise lies in evolutionary biology, theoretical biology, microbial evolution and palaeontology.

3. How much time do you estimate the authors will need to complete the suggested revisions:

Estimated time to Complete Revisions (Required)

(Decision Recommendation)

Less than 1 month

4. Review Commons values the work of reviewers and encourages them to get credit for their work. Select 'Yes' below to register your reviewing activity at Publons; note that the content of your review will not be visible on Publons.

Reviewer Publons

Yes

Revision Plan

Manuscript number: RC-2022-01576
Corresponding author(s): Enrico Sandro, Colizzi

[The "revision plan" should delineate the revisions that authors intend to carry out in response to the points raised by the referees. It also provides the authors with the opportunity to explain their view of the paper and of the referee reports.

The document is important for the editors of affiliate journals when they make a first decision on the transferred manuscript. It will also be useful to readers of the reprint and help them to obtain a balanced view of the paper.

If you wish to submit a full revision, please use our "Full Revision" template. ***It is important to use the appropriate template to clearly inform the editors of your intentions.****]*

1. General Statements [optional]

We are pleased to transfer our manuscript, titled "Evolution of genome fragility enables microbial division of labor", from Review Commons to Molecular Systems Biology, following the encouraging initial comments from the reviewers. The study focuses on developing and studying a computational model of microbial evolution, inspired by recent experimental results on the antibiotic-producing bacterium *Streptomyces Coelicolor*.

In general, the revisions required to address the reviewers' comments involve mostly text revision. We will answer each point raised by the reviewers and sharpen the text where the reviewers found it unclear. Reviewer 3 asks for additional simulations, which we are happy to carry out.

2. Description of the planned revisions

Reviewer #1 (Evidence, reproducibility and clarity (Required)):

This study proposes (and uses) an elegant model of bacteria evolution to study how division of labor can emerge through the interaction between non-random mutations (occurring at some specific ``fragile' genomic sites) and genome architecture. The study is very interesting and the results are convincing. My main concerns are about the presentation of the model and results. Although I am confident about the results, some elements should be clarified for a better understanding and for a correct interpretation of the results. Two points in particular (detailed below as major comments) require clarification.

Revision Plan

Major comments:

- the notion of telomere/centromere is used all throughout the paper but I think it is used in a misleading way. First, it seems that here there is only one telomere (but this is actually a detail of the model). More importantly, as long as I know, it is well known that in S. coelicolor the sequence degenerates more rapidly when getting closer to the telomeres (but telomeres are defined independently from this property). But here, the notion of telomere is precisely directly determined by its mutational instability (respectively, the centromere is defined by its stability). Although this is reasonable given the objective of the model, it forbid the use of sentences like "we observed that the genome of the evolved colony founded had two distinct regions: a telomeric [...] and a centromeric [...]" (line 234) or "When bacteria divide, mutations induced at fragile sites lead to the deletion of the part of the genome distal to them, causing large telometic deletions" (line 239 - this is not a result but a hidden description of the model) as this distinction between the two regions is not an outcome of the simulation but rather given a priori as a coded property of the fragile sites that all lead to deletions on the same -- called telomeric -- side (of course, formally if the genome contains no fragile site, there is no distinction but still). Please clarify this in the main text and in the methods.

Authors response (AR, in the following): we agree with the reviewer that the directionality of the deletions determines centromere and telomere in our model (and the reviewer is correct that we only consider one arm of the chromosome). We will explicitly state both in the main text and in the methods that the model does not include any explicit centromeric and telomeric structure, and that the polarity of the genetic information (and thus centromere and telomere) depends on the choice of directionality of the deletions.

- In most part of the paper (methods, results, figures, sup mat...) antibiotics are considered to have a concentration (or a high/low production) but at least twice in the text (lines 165 and 488) it is said that only the presence/absence of antibiotics is modelled. I was not able to understand how the continuous values are transformed into presence/absence (is there a threshold?) but more importantly, I strongly suspect that this choice has a strong influence on the outcome. For instance, with a diffusion radius equals to 10, it means that an antibiotics producing cell is able to protect 2\pi*10=~60 replicating cells. Hence, one could conjecture that the fraction of antibiotic-producing mutants should a little more than 2%... which is what is observed by the authors. So (1) please clarify this point (2) discuss (or experiments) the consequences of this choice on the conclusion.*

AR: the reviewer is correct that antibiotics are modelled as presence/absence – this was done for computational efficiency. However, the probability that a bacterium deposits an antibiotic at a site within the deposition radius is a continuous number, as it depends on the number of antibiotic genes and growth genes. We will make this clear in the main text and in the methods. Secondly, we show the effect of varying the deposition radius for the evolutionary dynamics in Supplementary Section S17. We will make this clear in the main text. For the area covered by different radius of antibiotic deposition, please see below.

Minor comments:
- line 262: "We conclude that genome architecture is a key prerequisit for the maintenance of mutation-driven division of labor". Given the model hypotheses you cannot be so affirmative (it is a key prerequisit... in this model!)

AR: we will modify the statement as suggested.

- line 286: "cannot" is probably too strong. It has not been observed...

AR: we will modify the statement as suggested.

- line 288 and following: you seem to consider that there is "selection for diversity". Given the large number of possible antibiotics and given that cells are "automatically" resistant to the antibiotics they produce, could it be simply drift? There is a clear selection pressure to limit the number of growth-promoting genes but no such pressure exist for antibiotics. Hence their number could simply drift (note that figs 2 and SF1 both use a log scale; random variations due to drift could be hidden by the log. Fig. SF2 does use a log scale and shows a dynamics that---to my eyes---claims for drift rather than for selection of diversity).

AR: we agree with the reviewer that drift might contribute to the overall antibiotic diversity. This might be especially true for the antibiotic genes residing downstream of the fragile sites, which have low probability of expression in the wild-type (because of the many growth genes) and are deleted in the mutants. Duplications, deletions and modifications of these genes are effectively neutral, and are therefore likley subject to drift. We will include this discussion in the main text. However, bacteria are highly susceptible to the diverse antibiotics produced by other colonies (i.e. those produced – largely – by the mutants). These antibiotics and their diversity drives colony invasion and is thus selective. The overall number and diversity of antibiotics is therefore, at least in part, under selection.

- line 340: "ends" should be "end" when discussing the model
- line 345: "a telomeric region" should be "telomeric regions" when discussing the bacteria
- line 359: "S. ambofaciens" should be italic
- line 365: same for "Streptomyces"

AR: we will modify the statement as suggested (and thank the reviewer for carefully reading the text).

- line 245 states that colonies begin clonally but methods (lines 434-438) don't support this. Colonies don't begin clonally but they begin without antibiotic-producing spores (see also line 618)

AR: we agree with the reviewer that colonies are not specifically initialised as clonal. We will modify the sentence as: By this process colonies eventually evolve to become functionally differentiated throughout the growth cycle.

- line 442: "their" should be "its"
- line 446: "hotspot for recombination" no, for "deletion"
- line 449: please remove brackets around the reference.

AR: we will modify the statement as suggested.

- line 458: if I understood it correctly, there is no explicit competition in the model. Competition simply comes from the asynchronous replication. Am I true? Could you clarify that point?

AR: The reviewer is correct that through asynchronous updating only one focal lattice site is update at a time. However, if a site is empty, the bacteria surrounding it are competing based on their replication rate $k_{replication}$. Dividing by the neighbourhood size (eta) simply ensures that a bacterium surrounded by a completely empty neighborhood replicates on average alpha_g times (alpha_g being the max growth rate). We will mention this in the methods.

- line 490: "the antibiotic deposited is chosen randomly and uniformly among them". This is not fully clear. I suppose the bacteria is still resistant to all the antibiotics it \it{can} produce?

AR: Yes. This is mentioned in the methods section “Replication”.

- figure SF1: please use the same scales as in figure 2 such that the two plots can be easily compared

AR: we will modify the x-axis to include the number of growth cycles.

- section S3 and figure SF4: What is to be understood from the figure is not clear to me. Seems that WTs win only if generalists produce less AB or replicate slower (?) Is it true?

AR: The reviewer is correct. In other words: when the artificial generalist has the same replication rate and the same antibiotic production rate as the WT, then the competition experiment ends with a near draw (the generalist still wins, but slowly). This means that the fitness cost associated to division of labor, i.e. to having two cell types doing the same work as one generalist – is small.
We will include this description in the section.
The figure is unfortunately complicated by the fact that we do not know a-priori how high the effective antibiotic production rate is (because antibiotics are spatially distributed by the stochastically generated mutants) – and so we had to make a large parameter screen to figure out the parameter values for which the competition experiment made most sense.

- I found it very difficult to draw conclusion from section S4, S5 and S6. These experiments should be analyzed with the help of mathematical analyses of the equations. Moreover, the understanding of these results are rendered difficult due to the lack of clarity regarding the discrete (or not) nature of the antibiotic production/action/diffusion

AR: We hope that we have clarified the distinction between antibiotic production rate and antibiotic presence/absence in the lattice.
The model is not amenable to analytical tractability, which makes it difficult to make exact statements based on the equations that govern it. However, we can check that the model is robust, and identify regions of parameter space where the model behaves in a qualitatively similar way to main text results.
Sections S4, S5 and S6 are essentially parameter screens to verify that the model reproduces the results reported in the main text for a broad range of parameters. The primary conclusion that can be drawn is that the model is robust to parameter changes.
Section S4 explores the model robustness to changes in two key parameters of the model: the antibiotic inhibition due to growth genes beta_g and the parameter h_g, which is the number of growth genes that produces half-maximum growth rate. Section S5 further analyses the relation between these parameters, and how they together determine the strength of the trade-off. Section S6, finally, shows that a strong trade-off is not a necessary requirement for evolution of division of labor as the division also depends (in a counterintuitive way) on the parameter alpha_g, the maximum antibiotic production rate.

We will include and expand these summarizing statements in each section, to make clear what each section achieves.

- S7 and fig SF9. It is unclear to me why the fraction of mutants decrease along time elapsed in the cycle. Please explain.

AR: The reason is that not all mutants are born with the same number of antibiotic genes (Fig. 3A). A mutant with fewer antibiotic genes might be susceptible to some of the antibiotics produced by another mutant, and could be killed by these antibiotics. Once a mutant is killed in the inner colony, a wt will replicate to fill the spot, and likely a wt offspring will take that site rather than another mutant. Thus there is a decline in overall mutant population.
We will include this discussion in Section S7.

- Figure SF14: what are the tin lines? if they correspond to the five repeats, how can it be that the bold line be the median?

AR: we realise that the caption should be clearer. Each of the five lines (both bold and thin) in each pane represents the median number of genetic elements over time. The bold line just highlights one randomly chosen simulation (the same for each genetic element), to better guide the eye.
We will clarify the caption of the figure.

- S13 and figure SF15: given that AB concentration is ON/OFF, is this result really surprising? This also questions about the accumulation of AB genes in the original model. Although the authors regularly claim that this is due to selection for diversity, drift could also be at play (see above)

AR: As mentioned above, we agree with the reviewer and we will mention that drift may co-determine antibiotic gene accumulation.

- S17: for radius 1, 2 and 3, the aliasing is likely to be strong. Hence, the results cannot be interpreted with this sole information. Please give e.g. how many cells are "protected" for each radius (e.g. for r_{alpha}=1, this value can vary between 1 and 9!)

AR: for radius=1, 2, 3, 5 ,8, 10 the area covered by antibiotic production is respectively 5 ,13, 29, 81, 197, 317. We will inclue this information in the figure.

- L742: "matching the antibiotic bitstring with the bitstring of the antibiotic". True and actually elegant but simpler formulation could ease the reading...

AR: We will change the sentence as follows: “Both antibiotics and antibiotic genes are characterised by a bitstring, which determines their type. Antibiotic resistance in the model is determined by matching these two strings.”

- lines 746-751 and figure SF21: There again, could it be a consequence of the AB ON/OFF diffusion model?

AR: we agree with the reviewer that a continuous diffusion model could affect resistance to antibiotics. We expect that the main effect will come from some antibiotics antibiotics having different concentrations. For instance, we could have a situation in which many deleterious antibiotics are produced in small amount, but have a compounding effect on the susceptible bacterium. This finer model of antibiotic production, diffusion and killing was not included in the model to limit the computational load.

- S18-S19-S20: what should the reader understand from these results? Please better comment the figures.

AR: we agree that figures in Section S18,19 and 20 could have more descriptive captions. Sections S18, 19 and 20 are parameter screen to check that the model is robust to changes in the mutation rates affecting fragile sites activation and de-novo formation. The primary result of Section S18 is that that division of labor evolves over a broad range of fragile site activation rates and de-novo fragile site formation rates (and even when these parameters are decreased by one order of magnitude).

Section S19 shows how these combination of parameters result in quantitative changes in genome composition.
Section S20 shows that the de-novo fragile site formation rate can be zero: as long as the system is initialised genomes that can divide labor, the fragile sites will persist even though no new ones are generated.

CROSS-CONSULTATION COMMENTS
Sorry about the confusion about the computation of the number of cells protected by a single AB-producing cell. Of course it is of the order 10\pi^2 !!! The global argument still holds but the number of cells protected is of course larger than 60 (note that, due to aliasing at the periphery the exact number of cells in the protected area is difficult to determine).*

Author response: We hope the clarifications mentioned above answer the reviewer's comment.

Reviewer #1 (Significance (Required)):

First, an very importantly, I must say that I am no familiar with the biological model (Streptomyces coelicolor). So I am not fully able to judge the biological significance of this research (i.e. whether the way division of labor is achieved here enlights---or not---the biology of this bacteria). However, on the computational side, the model and the results (as they are summarized in the conclusion) are very interesting on their own and deserve publication.

Remark: a lots of supplementary results are added to the paper that are not not fully explained or analysed. Please, better discuss all these results and their significance.

AR: we will extensively check and add detail to the supplementary material, ensuring that results are fully explained (see also response to reviewer 1).

Reviewer #2 (Evidence, reproducibility and clarity (Required)):

The manuscript "Evolution of genome fragility enables microbial division of labor" presents a model of genetically-based division of labour in bacterial colonies. It is postulated that two essential processes, growth and the important for elimination of competitors production of antibiotics, are poorly compatible in a single cell. The beneficial for a colony cell specialization is assumed to be determined only by genetic differences that appear via deletions of growth- promoting loci. These deletions and production of various antibiotics are mediated by a rather elaborate genetic architecture, which includes position-sensitive "fragile" sites, mutable antibiotic and growth-promoting genes. The model produces rather predictable results that under sufficiently strong incompatibility between growth and antibiotic production, the long-term evolution results in formation of mosaic of colonies, each specialized in production of its specific set of antibiotics. Such production is facilitated by evolving rapidly mutable genomes that constantly generate non-reproducing antibiotic-pumping cells.

The model appears very thoroughly developed and analyzed, and all major conclusion are intuitively appealing. Overall, the manuscript reads as a well-written quantitative proof of the principle of genetically-based division of labour between bacterial cells. The only part of the model that I'm a bit sceptical about is the unwarranted complexity of the genetic architecture. Unless the introduction of "fragile" sites and the directional ordering of genes is strongly justified by empirical data, a simpler and more clear assumption about mutational incapacitation of growth genes would suffice to reproduce the predicted phenomenology. So adding such empirical evidence would boost the relevance of the genetical part of the model. In the present form, all observed adaptations are inevitable simply because the expected division of labour will not evolve without each of them due to the design of the model.

AR: We agree with the reviewer that a simpler model with a predetermined effect of mutations, such as to incapacitate the growth genes, would suffice to reproduce the phenomenology of the mutation-driven division of labor observed in Streptomyces. Adding the complexity of a genome architecture introduces one more hypothesis: that genome fragility can evolve to organize the division of labor. This hypothesis, supported by the results presented here, can be tested experimentally.

However, there is already some empirical support for our modelling choices: 1) mutation rates along the genome of Streptomyces are highly heterogeneous, 2) the genetic content is partitioned along the chromosome so that some genes are preferentially located in the mutationally quiet centromere, and others are in the mutationally active (sub)telomeric regions, 3) some cis genetic elements in Steptomyces' genomes readily recombine to produce large-scale duplications and deletions (which we heavily simplified in the model as deletion-inducing fragile sites).

We will extend the introduction to include the references for the empirical support to our model.

A couple of minor comments...

217 This is achieved when fewer growth-promoting genes are required to inhibit antibiotic
218 production (i.e. lower βg).
Shouldn't it be "larger \beta_g"?

AR: yes. Thanks for catching this!

Whether in the main text or Supplementary materials, it woud help to add a complete population dynamics equation with all gain and loss terms.

AR: we agree with the reviewer that it would be interesting to obtain a comprehensive population dynamics equation that captures the spatial dynamics of replication, mutation, and antibiotic production, causing colony formation and between-colony competition. However, deriving such equation would be a very big effort in itself, and we suspect that it would not be

analytically tractable. Because of this, we prefer the “procedural” model description we gave – which also mirrors the model implementation (see github repository at github.com/escolizzi/strepto2).

Strikingly, we find the opposite: division of labor evolves when
224 bacteria produce fewer overall antibiotics (lower αa), under shallow trade-off conditions
225 (hgβg = 5; see Suppl. Section S6).

I don't see why it is"striking". It seems perfectly explicable that a smaller \alpha requires more dedication to antibiotic production, thus favouring specialization.

AR: we agree that we have not conveyed why we found this result surprising. We have set the trade-off shallow enough (h_g beta_g =5) that the generalist wins when alpha_g =1. In addition, lowering alpha_a makes the benefit of creating a mutant smaller, because a highly specialised mutant with zero growth genes makes fewer antibiotics. A generalist is proportionally less affected. Intuitively, we have compunded two benefits for the generalist.

But division of labor evolves, outcompeting the generalist – which surprised us.

We will modify the paragraph to better explain what we expected, and we will tone down the wording, removing the word “strikingly”.

Reviewer #2 (Significance (Required)):

Due to my relative lack of familiarity with the literature on evolution of genetically-based division of labour, I would rather not comment on the degree of innovation of the manuscript.

The text is well written and is accessible to a wide readership, so it could be recommended to a general biological or evolutionary journal.

Reviewer #3 (Evidence, reproducibility and clarity (Required)):

Summary:
In this manuscript the authors explore the co-evolution of genomic architecture and division of labour in antibiotic production, in a model inspired by the bacterium Streptomyces coelicolor. In the model a genetic trade-off is implemented where the having a large number of growths promoting genes (and thus fast growth) leads to a low production of antibiotics. On the other hand, having fewer growth promoting genes allows for a higher production of antibiotics. This trade-off selects for a division of labour, where one sub population specializes in antibiotic production and another sub population specializes in reproduction. This division of labour is achieved by evolving the genome structure, so that growth promoting genes are clustered

together, separated from the rest of the genome by several fragile sites (sites that allow for large deletions). This allows a single mutational event to delete a large number of growth-promoting genes, which creates a cell, lacking growth genes and that thus has a high antibiotic production (cell specializing in antibiotic production). In other words, the genome structure evolves to shape evolvability, so as to allow cells with a high growth rate to rapidly and repeatably evolve/mutate into cells with a high antibiotic production. This creates a division of labour where a part of the population specializes in growth/reproduction and another part specializes in antibiotics production. This model provides a tangible mechanism to explain a similar division of labour observed in S. coelicolor. This mechanism also fits well with the large deletions observed in antibiotic-hyperproducing S. coelicolor cells, which are also repeatably generated during colony growth.

Major comments:
-Line 69, It would be good to give a bit more information here on the (number of) different types of antibiotics produced by S. coelicolor, to help the reader understand some of the modelling choices later on, such as allowing for the evolution of a large number (16 or higher if I understand correctly) of different antibiotics and a cell automatically being resistant to all antibiotics it produces (instead of having separate resistance genes).

AR: we agree with the reviewer that adding this information would put the model more in focus. The total number of antibiotics that can be produced by the genus Streptomyces has been estimated to be of the order of 100000 (ten to the fifth, [Watve et al., 2001]). Although we use S. Coelicolor as reference model organism for our computational model, we simulate long-term evolutionary dynamics that diversify the antibiotic repertoire. Each antibiotic is represented by a 16 bits string, meaning that there are 2^16 (= 65536) possible antibiotics in the system – consistent with the number of possible antibiotics in the genus.
This being said, our model genomes evolve to have many more antibiotic genes than typical Streptomyces. Each species in the genus has up to 30 biosynthetic gene clusters [Genilloud, O. (2014)], a fraction of which make antibiotics. We discuss this discrepancy and propose solutions for this in the Discussion (also see below).

Regarding the possibility of separating antibiotic resistance from antibiotic synthesis: we (and most literature on the eco-evolutionary dynamics of antibiotic-producing bacteria) simplified antibiotic production as depending on individual “antibiotic biosynthetic genes”. In reality several genes in a cluster must be expressed to synthesize an antibiotic. A typical biosynthetic gene cluster also encodes resistance genes for the cognate antibiotic, to prevent cell suicide [Mak et al., 2014] – hence antibiotic genes providing resistance in the model. This being said, Streptomyces genomes also host resistance genes to antibiotics for which they have no biosynthetic pathway themselves, including efflux pumps that give some nonspecific resistance [Nag et al 2021].
Modelling antibiotic synthesis in more detail would allow to make a better model of antibiotic evolution, as well as to enrich the social dynamics of the model – because “cheaters” could

evolve that are resistant but do not contribute to the antibiotics in the colony. These questions are certainly interesing, but would further complexify the model. They are exciting venues for future model expansions.

We will include the literature mentioned above in the introduction, and use these references to better motivate the model.

-Lines 127-129 It is mentioned here fragile sites in the genome might represent transposable elements or long inverted repeats. Would both of these types of fragile sites behave the same? Has it been shown that both transposable elements and long inverted repeats can lead to large deletions from a linear chromosome? It would be nice to have a bit more background on how fragile sites might work or what they might look like in an empirical context. I am a bit unsure on this, but depending on their exact empirical nature, should fragile sites not also lead to increased rates of gene duplication near themselves?

AR: we see that we have not made a clear connection between the introduction, where we introduce the mutational dynamics of Streptomyces, and the methods, where we introduce fragile sites.

Briefly, both duplications and deletions occur in Streptomyces, as well as circularization of the linear chromosome, conjugation, etc. [Hoff et al 2018,Tidjani et al 2019]. However, the outcome of all these mutations is biased towards deletion [hoff et al 2018, Zhang et al, 2020, Zhang et al, 2022]. There are many mechanisms involved in producing these mutations, forming the mutational hotspots, handling DNA breaks, and in the horizontal transfer of genetic material [Tidjani et al 2019; Lorentzi et al, 2021]. As the reviewer suggests – they do not behave all in the same way. To construct the model, we simplified all these mutational mechanisms into one genetic element, the “fragile site”, and assumed that they are solely responsible for the chromosomal-scale mutations that produce deletions.

We will add this information to the introduction (see also response to reviewer 2), and refer to it in the methods.

-Line 160 As alluded to before, given the introduction provided, two assumptions come about here (lines 160-166) that lack a bit of justification/background/context. First, why does one allow the evolution of such a relatively large number of antibiotics? A bit more empirical in the introduction background would go a long way to making this assumption seem more justified. As far as I can see the genomic architecture leading to division of labour is only demonstrated for values of v that are 6 (i.e. 64 antibiotics) or above. Perhaps it is because I lack empirical background here, but this still seems to be a relatively large antibiotic space. Does the model also work with v=2? Perhaps it would be good to show a simulation with v=2 in supplementary material S16 as well.

AR: Hopefully the previous comment on the number of possible antibiotics also clarifies this point.
We will carry out a simulation with v=2.

-Line 166 The assumption is made that if a bacterium produces a certain antibiotic, it is automatically resistant to this antibiotic. Now it could be that this assumption is empirically rooted, in which case it would be good to allude to this empirical justification. I wonder how would the results be impacted if the resistance genes were separated from the antibiotic production genes? (I do not think additional simulations are in any way necessary on this point, but some more context/thoughts on this matter would be helpful, perhaps near lines 306-309)

AR: Please see response to major comment on the possibility of separating antibiotic resistance from antibiotic synthesis. We will add the discussion there in the Discussion session.

-Figure 1 In the subscript it becomes evident that the probability of large deletions due to fragile sites is much higher (10 fold) than single gene duplications, it seems to me this should be the other way around, single gene duplications and deletions could be much more probable than fragile site induced large deletions. Would the model still produce the same results if the values for mu-d and mu-f were switched around? (Again, I do not think additional simulations are per se required, some justification for this assumption would already be plenty).

AR: We chose these parameter values because, empirically, large scale chromosomal rearrangements (deletions) occur more frequently than single gene duplication/deletion in Streptomyces – as they are the primary mechanism for Streptomyces development and division of labor. We now mention this in the caption of Fig. 1.

Still, would we expect results to be affected if mu_d > mu_f? We do not think so, for the following reason: mu_d and mu_f are per-gene probabilities, so the genomic probability of duplication/deletion and of fragile site activation will depend on the evolved number of genes. in Fig. 5 we show that mu_f can be decreased by more than one order of magnitude and results do not change qualitatively. To compensate for a smaller per-gene deletion rate (mu_f), the evolved number of fragile sites per genome becomes larger (Suppl. Section S19, Fig. SF23). A similar compensatory increase of fragile sites could happen if duplications and deletions rate per gene were larger.

Minor comments:
-Line 36, perhaps replace "must" with " can" as there are other ways to achieve a division of labour that do not hinge on genomic architecture such as those listed in the next sentence. This sentence seems at odds with the next one, which lists ways to achieve cell differentiation that do not per se completely rely on genomic architecture such as gene regulation. Maybe consider moving this sentence to be on line 40 (after "...organized at the genome level remains unclear")

AR: we will modify the text as suggested by the reviewer

-Line 48, perhaps remove "disposable" as there is no particular reason the somatic tissue is disposable, furthermore it invokes the disposable soma theory of aging which is not relevant here

AR: we will remove “disposable”.

-Line 147-148 Why these particular relationships, as a reader I do not understand how these functions were constructed and how they might influence the results, a bit more justification might be helpful. Perhaps later on (results/discussion) also address what might happen if you were to use different functions?

AR: we agree that these functions could use a little more explanation. The probability of replication is a function that increases with the number of growth genes. We assume that the function saturates, as growth cannot be arbitrarily large even if the genome hosts many growth genes. So we need at least two parameters: one for the maximum growth rate (alpha_g), and another that controls the curvature of the function (h_g). A simple choice is a Hill function, but other saturating functions would likely work just as well (e.g. an exponential function with a form alpha_g*(1-exp(-g/h_g)). Similarly, antibiotic synthesis inhibition from growth genes should tend to zero for larger numbers of growth genes, hence the exponential (but we expect that a hyperbolic form e.g 1/(1+g/beta_g) would work just the same).

As this discussion is rather technical, we will include it in the methods section.

-I am clearly biased on this matter, since I work on evolvability. So, the authors should feel free to ignore this comment. Regardless, I think the authors have shown a wonderful example of the evolution of evolvability. Perhaps it would be nice to add a little bit of an evolvability angle in the discussion. In particular thinking about how fragile sites shape evolvability.

AR: we agree with the reviewer that the work is a clear form of evolution of evolvability. We now explicitly mention this in the discussion.

-Lines 404-411 It is great to see that the authors consider the wider applicability of their findings. It would be nice to add something here about the broader applicability in bacteria. As a large number of bacteria have circular chromosomes, how would these findings be impacted if circular chromosomes were at play? (I suspect they would largely still work in the same way, but keen to hear what the authors think). Referring to the work of Yona et al. 2012 on transient chromosomal duplications in yeast due to heat stress might also be good here, to show the more general applicability of the authors findings, this is another example where genomic architecture shapes evolvability.

Yona AH, Manor YS, Herbst RH, Romano GH, Mitchell A, Kupiec M, Pilpel Y, Dahan O. Chromosomal duplication is a transient evolutionary solution to stress. Proc Natl Acad Sci U S A. 2012 Dec 18;109(51):21010-5. doi: 10.1073/pnas.1211150109. Epub 2012 Nov 29. PMID: 23197825; PMCID: PMC3529009.

AR: Bacteria show many forms of targeted mutational dynamics (we do already mention CRISPR and HGT). It recently came to our attention that many bacterial and archea genomes host so-called Diversity-Generating Retroelements (DGR) [Macadangdang et al, 2022]. DGRs accelerate microbial evolution at specific sites and generate functional diversity. We will include this reference in the discussion.
We thank the reviewer for pointing us to the work on chromosomal duplication in yeast – we will also incorporate this “dramatic” form of duplication in the discussion.

-Lines 412 -419 I agree with the authors that in practice the cells specializing in antibiotic production look somewhat like soma, however I would consider not using this term here as strictly speaking the antibiotics producing cells can still reproduce (be it at an extremely low rate, which leads to their loss).

AR: We tone down both mentions of soma, as follows: “This gives rise to a division of labor driven by mutation, reminiscent of the division between germ and soma in multicellular eukaryotes.”
And, in the last sentence, we write: “...mutant cells *effectively* function as soma by enhancing...”

- Lines 434-438 If I understand correctly authors did not explicitly model the sporulation process (instead selecting random cells from the end of a cycle). I think this is a very good modelling choice that should not be changed; however, I do wonder how the results would be affected if sporulation was more explicitly modelled (for example by adding genes for sporulation, creating a 3 way trade-off between growth, sporulation and antibiotic production). Perhaps something that could be mentioned in the discussion.

AR: we agree with the reviewer that more complex evolutionary problem could be implemented in the system, e.g. through a gene type required for sporulation. They would likely have interesting outcomes. For instance, some bacteria may decide never to sporulate, while others could enhance their antibiotic resistance by turning into spores. Moreover, including additional functions together with an evolvable gene regulation could better capture the developmental dynamics observed through the life cycle of Streptomyces.

I hope this review is of some use and helps the improvement of this manuscript.

Yours sincerely,

Timo van Eldijk

Reviewer #3 (Significance (Required)):

Significance:
This study provides a clear conceptual advance by showing and studying how genome structure can evolve to create a division of labor. Thereby mechanistically explaining the division of labor in antibiotic production observed in S. coelicolor. It seems evident to me that whilst this study mainly focuses on S. coelicolor, the mechanism likely plays an important role in microbial evolution in general. Though others have previously theoretically explored such mechanisms, this study provides the first exploration modelled closely after an empirical system and hence provides a significant advance. In a more general sense, the evolution of genome architecture likely governs evolvability not just in microbes but in all life on earth. Therefore, I believe that this paper would be interesting for a general audience interested evolution. It would be of particular interest to those studying microbial evolution. My expertise lies in evolutionary biology, theoretical biology, microbial evolution and palaeontology.

3. Description of the revisions that have already been incorporated in the transferred manuscript

Please insert a point-by-point reply describing the revisions that were already carried out and included in the transferred manuscript. If no revisions have been carried out yet, please leave this section empty.

4. Description of analyses that authors prefer not to carry out

Please include a point-by-point response explaining why some of the requested data or additional analyses might not be necessary or cannot be provided within the scope of a revision. This can be due to time or resource limitations or in case of disagreement about the necessity of such additional data given the scope of the study. Please leave empty if not applicable.

Minor comment of reviewer 2:
Whether in the main text or Supplementary materials, it would help to add a complete population dynamics equation with all gain and loss terms.

Author response: we agree with the reviewer that it would be interesting to obtain a comprehensive population dynamics equation that captures the spatial dynamics of replication, mutation, and antibiotic production, causing colony formation and between-colony competition. However, deriving such equation would be a very big effort in itself, and we suspect that it would not be analytically tractable. Because of this, we prefer the “procedural” model description we gave – which also mirrors the model implementation (see github repository at github.com/escolizzi/strepto2).

1st Editorial Decision **15th Sep 2022**

Thank you for the submission of your manuscript to Molecular Systems Biology. I have now had a chance to carefully read your manuscript and the revision plan. We think the study is of potential interest, and we would like to invite a major revision of your manuscript.

All issues raised by the reviewers need to be satisfactorily addressed. In particular, Reviewers #2 and #3 requested additional empirical data and evidence for the model assumption (especially about the concept of 'fragile sites'), which must be carefully addressed.

As you may already know, our editorial policy allows in principle a single round of major revision, and it is therefore essential to respond to the reviewers' comments that are as complete as possible.

On a more editorial level, we would ask you to address the following issues:

Full Revision

Manuscript number: RC-2022-01576
Corresponding author(s): Enrico Sandro, Colizzi

[Please use this template only if the submitted manuscript should be considered by the affiliate journal as a full revision in response to the points raised by the reviewers.

If you wish to submit a preliminary revision with a revision plan, please use our "Revision Plan" template. ***It is important to use the appropriate template to clearly inform the editors of your intentions.****]*

1. General Statements [optional]

We are pleased to submit a full revision of our manuscript, titled "Evolution of genome fragility enables microbial division of labor", to Molecular Systems Biology. The study focuses on developing and studying a computational model of microbial evolution, inspired by recent experimental results on the antibiotic-producing bacterium *Streptomyces Coelicolor*.

We have carried out all the revisions previously planned (see Revision Plan). These revisions involved mostly changes to the text. We have answered each point raised by the reviewers and sharpened the text where the reviewers found it unclear. In particular, we have included empirical evidence regarding fragile sites and antibiotic biosynthesis, and used this evidence to better motivate the model. We also carried out the additional simulations asked by Reviewer 3, which we now mention in main text and detail in the Appendix.

2. Point-by-point description of the revisions

Reviewer #1 (Evidence, reproducibility and clarity (Required)):

This study proposes (and uses) an elegant model of bacteria evolution to study how division of labor can emerge through the interaction between non-random mutations (occurring at some specific ``fragile' genomic sites) and genome architecture. The study is very interesting and the results are convincing. My main concerns are about the presentation of the model and results. Although I am confident about the results, some elements should be clarified for a better understanding and for a correct interpretation of the results. Two points in particular (detailed below as major comments) require clarification.

Major comments:

- the notion of telomere/centromere is used all throughout the paper but I think it is used in a misleading way. First, it seems that here there is only one telomere (but this is actually a detail

of the model). More importantly, as long as I know, it is well known that in S. coelicolor the sequence degenerates more rapidly when getting closer to the telomeres (but telomeres are defined independently from this property). But here, the notion of telomere is precisely directly determined by its mutational instability (respectively, the centromere is defined by its stability). Although this is reasonable given the objective of the model, it forbid the use of sentences like "we observed that the genome of the evolved colony founded had two distinct regions: a telomeric [...] and a centromeric [...]" (line 234) or "When bacteria divide, mutations induced at fragile sites lead to the deletion of the part of the genome distal to them, causing large telometic deletions" (line 239 - this is not a result but a hidden description of the model) as this distinction between the two regions is not an outcome of the simulation but rather given a priori as a coded property of the fragile sites that all lead to deletions on the same -- called telomeric -- side (of course, formally if the genome contains no fragile site, there is no distinction but still). Please clarify this in the main text and in the methods.

Authors response (AR, in the following): we agree with the reviewer that the directionality of the deletions determines centromere and telomere in our model (and the reviewer is correct that we only consider one arm of the chromosome). We now explicitly state both in the main text and in the methods that the model does not include any explicit centromeric and telomeric structure, and that the polarity of the genetic information (and thus centromere and telomere) depends on the choice of directionality of the deletions, at Line 211-216 (main text), L. 531-534 (Methods). We also updated the text, at L 211,223, 324-328, 332, and caption of Fig. 3a.

- In most part of the paper (methods, results, figures, sup mat...) antibiotics are considered to have a concentration (or a high/low production) but at least twice in the text (lines 165 and 488) it is said that only the presence/absence of antibiotics is modelled. I was not able to understand how the continuous values are transformed into presence/absence (is there a threshold?) but more importantly, I strongly suspect that this choice has a strong influence on the outcome. For instance, with a diffusion radius equals to 10, it means that an antibiotics producing cell is able to protect 2\pi*10=~60 replicating cells. Hence, one could conjecture that the fraction of antibiotic-producing mutants should a little more than 2%... which is what is observed by the authors. So (1) please clarify this point (2) discuss (or experiments) the consequences of this choice on the conclusion.*

AR: the reviewer is correct that antibiotics are modelled as presence/absence – this was done for computational efficiency. However, the probability that a bacterium deposits an antibiotic at a site within the deposition radius is a continuous number, as it depends on the number of antibiotic genes and growth genes. We now make this clearer in the main text (L 198-199) and in the Methods, at L 697-707.
We mention that results are robust to changing the deposition radius at L 428, and in Appendix Section 17 (in the previous version of the manuscript this was “Suppl. Section S17”), which we have now reworded to better explain the results, including the number of lattice sites

corresponding to the radius of antibiotic deposition (in response to another comment from the reviewer, see below).

Minor comments:
- line 262: "We conclude that genome architecture is a key prerequisit for the maintenance of mutation-driven division of labor". Given the model hypotheses you cannot be so affirmative (it is a key prerequisit... in this model!)

AR: we modified the statement as suggested. Line 371

- line 286: "cannot" is probably too strong. It has not been observed...

AR: we modified the statement as suggested. *Line 411*

- line 288 and following: you seem to consider that there is "selection for diversity". Given the large number of possible antibiotics and given that cells are "automatically" resistant to the antibiotics they produce, could it be simply drift? There is a clear selection pressure to limit the number of growth-promoting genes but no such pressure exist for antibiotics. Hence their number could simply drift (note that figs 2 and SF1 both use a log scale; random variations due to drift could be hidden by the log. Fig. SF2 does use a log scale and shows a dynamics that---to my eyes---claims for drift rather than for selection of diversity).

AR: we agree with the reviewer that drift might contribute to the overall antibiotic diversity. This might be especially true for the antibiotic genes residing downstream of the fragile sites, which have low probability of expression in the wild-type (because of the many growth genes) and are deleted in the mutants. Duplication, deletion and type modification of these genes are effectively neutral, and are therefore likely subject to drift. However, bacteria are highly susceptible to the diverse antibiotics produced by other colonies (i.e. those produced – largely – by the mutants). These antibiotics and their diversity drive colony invasion and are thus adaptive. The overall number and diversity of antibiotics is therefore, at least in part, under selection.

We modified the main text to explicitly mention that a combination of drift and selection likely shapes the antibiotic repertoire in the model at L 414-415 and discuss it in more detail in Appendix 13

- line 340: "ends" should be "end" when discussing the model
- line 345: "a telomeric region" should be "telomeric regions" when discussing the bacteria
- line 359: "S. ambofaciens" should be italic
- line 365: same for "Streptomyces"

AR: we modified the statement as suggested (and thank the reviewer for carefully reading the text).

- line 245 states that colonies begin clonally but methods (lines 434-438) don't support this. Colonies don't begin clonally but they begin without antibiotic-producing spores (see also line 618)

AR: we agree with the reviewer that colonies are not specifically initialised as clonal. We modified the sentence as: By this process colonies eventually evolve to become functionally differentiated throughout the growth cycle. Line 335-337.

- line 442: "their" should be "its"
- line 446: "hotspot for recombination" no, for "deletion"
- line 449: please remove brackets around the reference.

AR: we modified the statement as suggested.

- line 458: if I understood it correctly, there is no explicit competition in the model. Competition simply comes from the asynchronous replication. Am I true? Could you clarify that point?

AR: Through asynchronous updating, only one focal lattice site is updated at a time. However, if a site is empty, the bacteria surrounding it are competing based on their replication rate $k_{replication}$. Dividing by the neighbourhood size (eta) simply ensures that a bacterium surrounded by a completely empty neighborhood replicates on average alpha_g times (alpha_g being the max growth rate). We now mention this in the Methods, at L 644-650.

- line 490: "the antibiotic deposited is chosen randomly and uniformly among them". This is not fully clear. I suppose the bacteria is still resistant to all the antibiotics it \it{can} produce?

AR: Yes. This is mentioned in the methods section “Replication”. *See Line 640-641.*

- figure SF1: please use the same scales as in figure 2 such that the two plots can be easily compared

AR: we modified the x-axis to include the number of growth cycles.
For the sake of consistency, we also modified the x axis of Fig. SF2.

- section S3 and figure SF4: What is to be understood from the figure is not clear to me. Seems that WTs win only if generalists produce less AB or replicate slower (?) Is it true?

AR: The reviewer is correct. In other words: when the artificial generalist has the same replication rate and the same antibiotic production rate as the WT, then the competition experiment ends with a near draw (the generalist still wins, but slowly). This means that the fitness cost associated with division of labor, i.e. having two cell types doing the same work as one generalist, is small.
We now include this description in the section (see Appendix 3, Line 63-68 and Line 84-88).

The figure is slightly complicated by the fact that we do not know a priori how high the effective antibiotic production rate is (because antibiotics are spatially distributed by the stochastically generated mutants) – and so we had to perform a large parameter screen to figure out the parameter values for which the competition experiment made most sense.

- I found it very difficult to draw conclusion from section S4, S5 and S6. These experiments should be analyzed with the help of mathematical analyses of the equations. Moreover, the understanding of these results are rendered difficult due to the lack of clarity regarding the discrete (or not) nature of the antibiotic production/action/diffusion

AR: We hope that we have clarified the distinction between antibiotic production rate and antibiotic presence/absence in the lattice.
The model is not analytically tractable, which makes it difficult to make exact statements based on the equations that govern it. However, we can check that the model is robust, and identify regions of parameter space where the model behaves in a qualitatively similar way to main text results.
Sections S4, S5 and S6 are essentially parameter screens to verify that the model reproduces the results reported in the main text for a broad range of parameters. The primary conclusion that can be drawn is that the model is robust to parameter changes.
Section S4 explores the model robustness to changes in two key parameters of the model: the antibiotic inhibition due to growth genes beta_g and the parameter h_g, which is the number of growth genes that produces half-maximum growth rate. Section S5 further analyses the relation between these parameters, and how they together determine the strength of the trade-off.
Section S6, finally, shows that a strong trade-off is not an essential requirement for evolution of division of labor. In fact, whether division of labor evolves depends on the parameter alpha_g, the maximum antibiotic production rate, in a counter-intuitive way.

We have included and expanded these summarizing statements in each section, to make clear what each section achieves.

- S7 and fig SF9. It is unclear to me why the fraction of mutants decrease along time elapsed in the cycle. Please explain.

AR: The reason is that not all mutants are born with the same number of antibiotic genes (Fig. 3A). A mutant with fewer antibiotic genes might be susceptible to some of the antibiotics produced by another mutant, and could be killed by these antibiotics. Once a mutant is killed in the inner colony, a wildtype will replicate to fill the spot, and likely a wildtype offspring will take that site rather than another mutant. Thus there is a decline in overall mutant population.
We have included this discussion in Section S7. See Lines 197-205

- Figure SF14: what are the tin lines? if they correspond to the five repeats, how can it be that the bold line be the median?

AR: Each of the five lines (both bold and thin) in each pane represents the median number of genetic elements over time. The bold line just highlights one randomly chosen simulation (the same for each genetic element), to better guide the eye.
We clarified the caption of the figure.

- S13 and figure SF15: given that AB concentration is ON/OFF, is this result really surprising? This also questions about the accumulation of AB genes in the original model. Although the authors regularly claim that this is due to selection for diversity, drift could also be at play (see above)

AR: As mentioned above, we agree with the reviewer and we now mention that drift may co-determine antibiotic gene accumulation.

- S17: for radius 1, 2 and 3, the aliasing is likely to be strong. Hence, the results cannot be interpreted with this sole information. Please give e.g. how many cells are "protected" for each radius (e.g. for r_{alpha}=1, this value can vary between 1 and 9!)

AR: for radius=1, 2, 3, 5 ,8, 10 the area covered by antibiotic production is respectively 5, 13, 29, 81, 197, 317 (these numbers are the solution to the so called “Gauss's Circle Problem”). We have included this information in the figure (now SF18).

- L742: "matching the antibiotic bitstring with the bitstring of the antibiotic". True and actually elegant but simpler formulation could ease the reading...

AR: We changed the sentence as follows: “Both antibiotics and antibiotic genes are characterised by a bitstring, which determines their type. Antibiotic resistance in the model is determined by matching these two strings.”
See Appendix Line 357.

- lines 746-751 and figure SF21: There again, could it be a consequence of the AB ON/OFF diffusion model?

AR: we agree with the reviewer that a continuous diffusion model could affect resistance to antibiotics. For instance, we could have a situation in which mildly deleterious antibiotics in small concentrations do not have hinder a susceptible bacterium. However, we also expect that results will remain qualitatively similar, because antibiotic production is strongly selected for – as it allows colonies to further invade the lattice. This more precise model of antibiotic production, diffusion and killing was not included in the model to limit the computational load.

We now include this discussion in the section, Appendix Lines 370-379 (we also briefly come back to it in the Discussion).

- S18-S19-S20: what should the reader understand from these results? Please better comment the figures.

AR: we agree that figures in Section S18,19 and 20 could use more descriptive captions. Sections S18, 19 and 20 are parameter screens to check that the model is robust to changes in the mutation rates affecting fragile site activation and de-novo formation. The primary result of Section S18 is that that division of labor evolves over a broad range of fragile site activation rates and de-novo fragile site formation rates (even when these parameters are decreased by one order of magnitude).
Section S19 shows how these combination of parameters result in quantitative (but not qualitative) changes in genome composition.
Section S20 shows that the de-novo fragile site formation rate can be zero: as long as the system is initialized with genomes that can divide labor, the fragile sites will persist even though no new ones are generated.

We have expanded the caption of the figures, and re-written parts of Appendix section 18, 19, 20 to make this clearer.

CROSS-CONSULTATION COMMENTS
Sorry about the confusion about the computation of the number of cells protected by a single AB-producing cell. Of course it is of the order 10\pi^2 !!! The global argument still holds but the number of cells protected is of course larger than 60 (note that, due to aliasing at the periphery the exact number of cells in the protected area is difficult to determine).*

Author response: We hope that the answers to the reviewer's comments sufficiently clarified the matter.

Reviewer #2 (Significance (Required)):

First, an very importantly, I must say that I am no familiar with the biological model (Streptomyces coelicolor). So I am not fully able to judge the biological significance of this research (i.e. whether the way division of labor is achieved here enlights---or not---the biology of this bacteria). However, on the computational side, the model and the results (as they are summarized in the conclusion) are very interesting on their own and deserve publication.

Remark: a lots of supplementary results are added to the paper that are not not fully explained or analysed. Please, better discuss all these results and their significance.

AR: we have extensively revised the supplementary material, ensuring that they are introduced clearly and results are fully explained (see also response to reviewer 1).

Reviewer #2 (Evidence, reproducibility and clarity (Required)):

The manuscript "Evolution of genome fragility enables microbial division of labor" presents a model of genetically-based division of labour in bacterial colonies. It is postulated that two essential processes, growth and the important for elimination of competitors production of antibiotics, are poorly compatible in a single cell. The beneficial for a colony cell specialization is assumed to be determined only by genetic differences that appear via deletions of growth- promoting loci. These deletions and production of various antibiotics are mediated by a rather elaborate genetic architecture, which includes position-sensitive "fragile" sites, mutable antibiotic and growth-promoting genes. The model produces rather predictable results that under sufficiently strong incompatibility between growth and antibiotic production, the long-term evolution results in formation of mosaic of colonies, each specialized in production of its specific set of antibiotics. Such production is facilitated by evolving rapidly mutable genomes that constantly generate non-reproducing antibiotic-pumping cells.

The model appears very thoroughly developed and analyzed, and all major conclusion are intuitively appealing. Overall, the manuscript reads as a well-written quantitative proof of the principle of genetically-based division of labour between bacterial cells. The only part of the model that I'm a bit sceptical about is the unwarranted complexity of the genetic architecture. Unless the introduction of "fragile" sites and the directional ordering of genes is strongly justified by empirical data, a simpler and more clear assumption about mutational incapacitation of growth genes would suffice to reproduce the predicted phenomenology. So adding such empirical evidence would boost the relevance of the genetical part of the model. In the present form, all observed adaptations are inevitable simply because the expected division of labour will not evolve without each of them due to the design of the model.

AR: We agree with the reviewer that a simpler model with a predetermined effect of mutations, such as to incapacitate the growth genes, would suffice to reproduce the phenomenology of the mutation-driven division of labor observed in *Streptomyces*. However, the complexity of genome architecture introduces one more hypothesis: that genome fragility can evolve to organize the division of labor. This hypothesis, supported by the results presented here, can be tested experimentally, e.g. by engineering the position of these sequences, and then checking whether they correlate with higher mutation rates; or by studying whether mutant frequency correlates with the number of fragile sites in different species.

We now better introduce the existing empirical support for fragile sites and use these references to better motivate the model: 1) Fragile sites are common in genomes across the whole tree of life [Mei et al 2021] 2) mutation rates along the genome of *Streptomyces* are highly heterogeneous – with some locations being hotspots for mutations, 3) the genetic content is partitioned along the chromosome so that some genes are preferentially located in the mutationally quiet centromere, and others are in the mutationally active (sub)telomeric regions, 4) some cis-genetic elements, typically the sequences of transposable elements, in Steptomyces' genomes readily recombine to produce large-scale duplications and, more prevalently, deletions [chen et al 2002] (which we heavily simplified in the model as deletion-inducing fragile sites).

Review COMMONS

See Line 108-112, L 151-157 and L 219-223 (also see reply to reviewer 3).

A couple of minor comments...

217 This is achieved when fewer growth-promoting genes are required to inhibit antibiotic 218 production (i.e. lower βg).
Shouldn't it be "larger \beta_g"?

AR: yes. Thanks for catching this! (L 286)

Whether in the main text or Supplementary materials, it would help to add a complete population dynamics equation with all gain and loss terms.

AR: we agree with the reviewer that it would be interesting to obtain a comprehensive population dynamics equation that captures the spatial dynamics of replication, mutation, and antibiotic production, causing colony formation and between-colony competition. However, deriving such equation would be a very big effort in itself, and we suspect that it would not be analytically tractable. Because of this, we prefer the "procedural" model description we gave – which also mirrors the model implementation (see github repository at github.com/escolizzi/strepto2).

Strikingly, we find the opposite: division of labor evolves when 224 bacteria produce fewer overall antibiotics (lower αa), under shallow trade-off conditions 225 (hgβg = 5; see Suppl. Section S6).

I don't see why it is"striking". It seems perfectly explicable that a smaller \alpha requires more dedication to antibiotic production, thus favouring specialization.

AR: we agree that we have not conveyed why we found this result surprising. We have set the trade-off shallow enough (h_g beta_g =5) that the generalist wins when alpha_g =1. In addition, lowering alpha_a makes the benefit of creating a mutant smaller, because a highly specialised mutant with zero growth genes makes fewer antibiotics. A generalist is proportionally less affected. Intuitively, we have compounded two benefits for the generalist.
But the division of labor strategy outcompetes the generalist – which surprised us.

Indeed, a smaller alpha_a has the effect of expanding the range of trade-off strengths for which division of labor evolves.
We have modified the paragraph (l 289-295) to better explain what we expected (see also Suppl. Section S6), and we toned down the wording, removing the word "strikingly".

Reviewer #2 (Significance (Required)):

Due to my relative lack of familiarity with the literature on evolution of genetically-based division of labour, I would rather not comment on the degree of innovation of the manuscript.

The text is well written and is accessible to a wide readership, so it could be recommended to a general biological or evolutionary journal.

Reviewer #3 (Evidence, reproducibility and clarity (Required)):

Summary:
In this manuscript the authors explore the co-evolution of genomic architecture and division of labour in antibiotic production, in a model inspired by the bacterium Streptomyces coelicolor. In the model a genetic trade-off is implemented where the having a large number of growths promoting genes (and thus fast growth) leads to a low production of antibiotics. On the other hand, having fewer growth promoting genes allows for a higher production of antibiotics. This trade-off selects for a division of labour, where one sub population specializes in antibiotic production and another sub population specializes in reproduction. This division of labour is achieved by evolving the genome structure, so that growth promoting genes are clustered together, separated from the rest of the genome by several fragile sites (sites that allow for large deletions). This allows a single mutational event to delete a large number of growth-promoting genes, which creates a cell, lacking growth genes and that thus has a high antibiotic production (cell specializing in antibiotic production). In other words, the genome structure evolves to shape evolvability, so as to allow cells with a high growth rate to rapidly and repeatably evolve/mutate into cells with a high antibiotic production. This creates a division of labour where a part of the population specializes in growth/reproduction and another part specializes in antibiotics production. This model provides a tangible mechanism to explain a similar division of labour observed in S. coelicolor. This mechanism also fits well with the large deletions observed in antibiotic-hyperproducing S. coelicolor cells, which are also repeatably generated during colony growth.

Major comments:
-Line 69, It would be good to give a bit more information here on the (number of) different types of antibiotics produced by S. coelicolor, to help the reader understand some of the modelling choices later on, such as allowing for the evolution of a large number (16 or higher if I understand correctly) of different antibiotics and a cell automatically being resistant to all antibiotics it produces (instead of having separate resistance genes).

AR: we agree with the reviewer that adding this information would put the model more in focus. The total number of antibiotics that can be produced by the genus Streptomyces has been estimated to be of the order of 100000 (10 to the 5th, [Watve et al., 2001]). Although we use *S. coelicolor* as reference model organism for our computational model, we simulate long-term evolutionary dynamics that diversify the antibiotic repertoire. Each antibiotic is represented by a

16 bits string, meaning that there are 2^16 (= 65536) possible antibiotics in the system – consistent with the number of possible antibiotics in the genus.
This being said, our model genomes evolve to have many more antibiotic genes than typical Streptomyces. Each species in the genus has up to 30 biosynthetic gene clusters [Genilloud, O. (2014)], a fraction of which make antibiotics. We discuss this discrepancy and propose solutions for this in the Discussion (also see below).

Regarding the possibility of separating antibiotic resistance from antibiotic synthesis: we (and most literature on the eco-evolutionary dynamics of antibiotic-producing bacteria) simplified antibiotic production as depending on individual “antibiotic biosynthetic genes”. In reality several genes in a cluster must be expressed to synthesize an antibiotic. A typical biosynthetic gene cluster also encodes resistance genes for the cognate antibiotic, to prevent cell suicide [Mak et al., 2014] – hence antibiotic genes providing resistance in the model. This being said, *Streptomyces* genomes also host resistance genes to antibiotics for which they have no biosynthetic pathway themselves, including efflux pumps that give some nonspecific resistance [Nag et al 2021].
Modelling antibiotic synthesis in more detail would allow to make a better model of antibiotic evolution, as well as to enrich the social dynamics of the model – because “cheaters” could evolve that are resistant but do not contribute to the antibiotics in the colony. These questions are certainly interesting, but would further complicate the model. They are exciting venues for future model expansions.

We have included the literature mentioned above in the introduction (Line 78 – 81), use these references to better motivate the model. Line 191-194, and come back to this in the Discussion, Line 524-530.

-Lines 127-129 It is mentioned here fragile sites in the genome might represent transposable elements or long inverted repeats. Would both of these types of fragile sites behave the same? Has it been shown that both transposable elements and long inverted repeats can lead to large deletions from a linear chromosome? It would be nice to have a bit more background on how fragile sites might work or what they might look like in an empirical context. I am a bit unsure on this, but depending on their exact empirical nature, should fragile sites not also lead to increased rates of gene duplication near themselves?

AR: we see that we have not made a clear connection between the introduction, where we introduce the mutational dynamics of *Streptomyces*, and the methods, where we introduce fragile sites.

Both duplications and deletions occur in *Streptomyces*, as well as circularization of the linear chromosome, conjugation, etc. [Hoff et al 2018,Tidjani et al 2019]. However, the outcome of all these mutations is biased towards deletion [Hoff et al 2018, Zhang et al, 2020, Zhang et al, 2022]. There are many mechanisms involved in producing these mutations, forming the mutational hotspots, handling DNA breaks, and in the horizontal transfer of genetic material

Review COMMONS

[chen et al 2002, Tidjani et al 2019; Lorentzi et al, 2021]. As the reviewer suggests – they do not behave all in the same way. To construct the model, we simplified all these mutational mechanisms into one genetic element, the “fragile site”, and assumed that they are solely responsible for the chromosomal-scale mutations that produce deletions.

We have added this information to the introduction (see also response to reviewer 2), and refer to it in the main text methods summary. See Line 108-112, L 151-157 and L 219-223

-Line 160 As alluded to before, given the introduction provided, two assumptions come about here (lines 160-166) that lack a bit of justification/background/context. First, why does one allow the evolution of such a relatively large number of antibiotics? A bit more empirical in the introduction background would go a long way to making this assumption seem more justified. As far as I can see the genomic architecture leading to division of labour is only demonstrated for values of v that are 6 (i.e. 64 antibiotics) or above. Perhaps it is because I lack empirical background here, but this still seems to be a relatively large antibiotic space. Does the model also work with v=2? Perhaps it would be good to show a simulation with v=2 in supplementary material S16 as well.

AR: Hopefully we have provided sufficient empirical justification for the large number of possible antibiotics in the answers to previous comments.
We carried out a simulation with v=2. We observe that division of labor does not evolve. We explain this as follows. If the number of antibiotics is very small, all bacteria can evolve full resistance with few antibiotic genes. Moreover, each antibiotic gene will give some protection for the other antibiotics (for instance, the antibiotic bitstring “10” gives some resistance to “00” and “11”, because their distance is 1), and at most two mutations are sufficient to convert any antibiotic type into any other, allowing fast evolution of resistance. Antibiotics thus exert smaller evolutionary pressure (for both defense and offense), and as a consequence bacteria increase their growth rate instead of dividing labor, minimizing the number of fragile sites in the process. We added these results to Appendix 16 (previously called “Suppl. Section S16”), see the new Appendix figure S20, and mention them at Line 426-428.
We expect that a very small number of possible antibiotics is not representative of actual *Streptomyces*, and soil microbial communities in general. These communities compete strongly and exchange a large amount of metabolites – many of which are antibiotics. In the model, we only consider *Streptomyces*, but in reality *Streptomyces* compete with many other microbial species. A smaller number of possible antibiotics would likely result in division of labor if we included other species that are susceptible to the antibiotics made by *Streptomyces*.

-Line 166 The assumption is made that if a bacterium produces a certain antibiotic, it is automatically resistant to this antibiotic. Now it could be that this assumption is empirically rooted, in which case it would be good to allude to this empirical justification. I wonder how would the results be impacted if the resistance genes were separated from the antibiotic production genes? (I do not think additional simulations are in any way necessary on this point, but some more context/thoughts on this matter would be helpful, perhaps near lines 306-309)

AR: Please see response to major comment on the possibility of separating antibiotic resistance from antibiotic synthesis. We have added the discussion there in the Discussion session. (L 524-530)

-Figure 1 In the subscript it becomes evident that the probability of large deletions due to fragile sites is much higher (10 fold) than single gene duplications, it seems to me this should be the other way around, single gene duplications and deletions could be much more probable than fragile site induced large deletions. Would the model still produce the same results if the values for mu-d and mu-f were switched around? (Again, I do not think additional simulations are per se required, some justification for this assumption would already be plenty).

AR: We chose these parameter values because, empirically, large scale chromosomal rearrangements (deletions) occur more frequently than single gene duplication/deletion in *Streptomyces* – as they are the primary mechanism for *Streptomyces* division of labor. From a genetic view point, we envision these mutations to arise from different processes. We assume that small scale duplications and deletions reflect the rare random mistakes occurring during DNA duplication, while fragile sites specifically enhance the likelihood of catastrophic failure of DNA replication, which results in large-scale deletion. We now mention this in the main text. L207-210.

Still, would results to be affected if single gene duplication/deletion (mu_d) occurred more frequently than large scale chromosomal deletions (mu_f)? We do not think so: in Fig. 5 we show that mu_f can be decreased by more than one order of magnitude and results do not change qualitatively. To compensate for a smaller mu_f, the evolved number of fragile sites per genome becomes larger (Appendix19, Fig. S24). A similar compensatory increase of fragile sites could happen if mu_d was larger. However, we expect that there is an upper limit to mu_d, which is due to duplicated genes being placed at random location in the genome (so that genome architecture does not arise trivially from it): if duplications and deletions occurred too frequently, we could get a scrambled genome, which would select against division of labor (as it occurs e.g. in Fig. 4). We now mention that there is a limit to how large mu_d can be in the Methods Section. Line 658-661.

Minor comments:
-Line 36, perhaps replace "must" with " can" as there are other ways to achieve a division of labour that do not hinge on genomic architecture such as those listed in the next sentence. This sentence seems at odds with the next one, which lists ways to achieve cell differentiation that do not per se completely rely on genomic architecture such as gene regulation. Maybe consider moving this sentence to be on line 40 (after "...organized at the genome level remains unclear")

AR: we modified the text as suggested by the reviewer. Rather than moving the sentence, we removed it altogether, as it was a bit redundant. Also, following editorial guidelines, we bundled that text with the conclusions, into a paragraph called Synopsis. L 30-49.

Review COMMONS

-Line 48, perhaps remove "disposable" as there is no particular reason the somatic tissue is disposable, furthermore it invokes the disposable soma theory of aging which is not relevant here

AR: we removed “disposable” and slightly modified the wording. L44-47.

-Line 147-148 Why these particular relationships, as a reader I do not understand how these functions were constructed and how they might influence the results, a bit more justification might be helpful. Perhaps later on (results/discussion) also address what might happen if you were to use different functions?

AR: we agree that these functions could use a little more explanation. The probability of replication is a function that increases with the number of growth genes. We assume that the function saturates, as growth cannot be arbitrarily large even if the genome hosts many growth genes. So we need at least two parameters: one for the maximum growth rate (alpha_g), and another that controls the curvature of the function (h_g). A simple choice is a Hill function, but other saturating functions would likely work just as well (e.g. an exponential function with a form alpha_g*(1-exp(-g/h_g)). Similarly, antibiotic synthesis inhibition from growth genes should tend to zero for larger numbers of growth genes, hence the exponential (but we expect that a hyperbolic form e.g 1/(1+g*beta_g) would work just the same).

As this discussion is rather technical, we have included it in the methods section.
See Line 620-630, Line 683-684, 693-695.

-I am clearly biased on this matter, since I work on evolvability. So, the authors should feel free to ignore this comment. Regardless, I think the authors have shown a wonderful example of the evolution of evolvability. Perhaps it would be nice to add a little bit of an evolvability angle in the discussion. In particular thinking about how fragile sites shape evolvability.

AR: we agree with the reviewer that the work is a clear form of evolution of evolvability. We now explicitly mention this in the discussion. Line 556-557 (we do comment on the evolvability of the (evolved) genome structure of *Streptomyces* in relation to fragile sites, at Line 503-505).

-Lines 404-411 It is great to see that the authors consider the wider applicability of their findings. It would be nice to add something here about the broader applicability in bacteria. As a large number of bacteria have circular chromosomes, how would these findings be impacted if circular chromosomes were at play? (I suspect they would largely still work in the same way, but keen to hear what the authors think). Referring to the work of Yona et al. 2012 on transient chromosomal duplications in yeast due to heat stress might also be good here, to show the more general applicability of the authors findings, this is another example where genomic architecture shapes evolvability.
Yona AH, Manor YS, Herbst RH, Romano GH, Mitchell A, Kupiec M, Pilpel Y, Dahan O. Chromosomal duplication is a transient evolutionary solution to stress. Proc Natl Acad Sci U S

A. 2012 Dec 18;109(51):21010-5. doi: 10.1073/pnas.1211150109. Epub 2012 Nov 29. PMID: 23197825; PMCID: PMC3529009.

AR: Bacteria show many forms of targeted mutagenesis in the context of a circular chromosome that produce functional phenotypes. The precise mechanism for these mutations varies wildly, showing that there are multiple possible ways – these or other mechanisms could be used to also organise a mutation-driven division of labor.
For instance, it recently came to our attention that many bacterial and archaea genomes host so-called Diversity-Generating Retroelements (DGR) [Macadangdang et al, 2022]. DGRs accelerate microbial evolution at specific sites and generate functional diversity.
We included the above discussion in the main text – and re-organised the paragraph to make its message clearer, L 565-575.
We thank the reviewer for pointing us to the work on chromosomal duplication in yeast – we have incorporated this "dramatic" form of duplication in the Discussion, L 555.

-Lines 412 -419 I agree with the authors that in practice the cells specializing in antibiotic production look somewhat like soma, however I would consider not using this term here as strictly speaking the antibiotics producing cells can still reproduce (be it at an extremely low rate, which leads to their loss).

AR: We tone down both mentions of soma, as follows: "This gives rise to a division of labor driven by mutation, reminiscent of the division between germ and soma in multicellular eukaryotes.", L 44-47
And, in the last sentence, we write: "...mutant cells *effectively* function as soma by enhancing...", L 48
However, we'd like to point out that most mutant cells in the model are completely sterile.

- Lines 434-438 If I understand correctly authors did not explicitly model the sporulation process (instead selecting random cells from the end of a cycle). I think this is a very good modelling choice that should not be changed; however, I do wonder how the results would be affected if sporulation was more explicitly modelled (for example by adding genes for sporulation, creating a 3 way trade-off between growth, sporulation and antibiotic production). Perhaps something that could be mentioned in the discussion.

AR: we agree with the reviewer that more complex evolutionary problem could be implemented in the system, e.g. through a gene type required for sporulation. They would likely have interesting outcomes. For instance, some bacteria may decide never to sporulate, while others could enhance their antibiotic resistance by turning into spores. Moreover, including additional functions together with an evolvable gene regulation could better capture the developmental dynamics observed through the life cycle of *Streptomyces*. Line 541-545

I hope this review is of some use and helps the improvement of this manuscript.

Full Revision

Yours sincerely,

Timo van Eldijk

Reviewer #3 (Significance (Required)):

Significance:
This study provides a clear conceptual advance by showing and studying how genome structure can evolve to create a division of labor. Thereby mechanistically explaining the division of labor in antibiotic production observed in S. coelicolor. It seems evident to me that whilst this study mainly focuses on S. coelicolor, the mechanism likely plays an important role in microbial evolution in general. Though others have previously theoretically explored such mechanisms, this study provides the first exploration modelled closely after an empirical system and hence provides a significant advance. In a more general sense, the evolution of genome architecture likely governs evolvability not just in microbes but in all life on earth. Therefore, I believe that this paper would be interesting for a general audience interested evolution. It would be of particular interest to those studying microbial evolution. My expertise lies in evolutionary biology, theoretical biology, microbial evolution and palaeontology.

Thank you for sending us your revised manuscript. We have now received the comments from the three referees who agreed to re-review your manuscript. As you will see below, the referees are overall satisfied with the modifications made, while they still raise several minor issues that need to be addressed.

Before we can formally accept your manuscript, we would ask you to address the following issues:

1. The remaining minor concerns of the reviewers. Please provide a letter with a detailed description of the changes made in response to the referees. Please specify clearly the exact places in the text (pages and paragraphs) where each change has been made in response to each specific comment given.

On a more editorial level:

Reviewer #1:

I am very sorry for a delay.

As I mentioned in my first review, this is a very impressive and thorough study and it became even better after the revisions. So I would recommend publishing it, yet I think the presented arguments would become even more convincing if the Authors consider commenting on the following two subjects:

Reading the explanations that were provided to my early comment about setting up the model is such a way that the evolution of genome architecture becomes inevitable, I still think that this should be stated more clearly: With the appropriate parameters, there is a selection pressure towards a division of labour in some form. Yet the only path to develop such a division available by the model setting is via the evolution of genome architecture. It is not stated (and I think it is definitely beyond the scope of this already complicated study) whether various other mechanisms to develop such a division, be it regulatory or other heritable modifications, could be more competitive in implementing the division of labour. Yet it would be good to be able to see such a disclaimer in the Discussion.

Given the multiply mentioned thoroughness of the manuscript, it would be excessive to ask for a full analysis, yet a qualitative estimate of an adaptive dynamics selection gradients towards the modification of the genome architecture, or at least mentioning them, would add to more intuitive interpretation of the findings.

Lines 200-201 "Resistance decreases when the difference between the two strings increases." Could it be shortly explained here or at least mentioned that the details are in Methods

Lines 431-432, "Only for an unrealistically small antibiotic deposition radius does division of labor not evolve." Word order?

Reviewer #2:

The manuscript in its present form is a very pleasant read and it very intuitive and easy to follow, really well done to the authors. My previous comments (see previous review) have been more than adequately addressed. The authors have especially made the link between the model and the empirical system much stronger. It is now really clear how the model assumptions come about. Furthermore, the authors have also expanded on the potential general applicability of their findings. All in all, I am more than happy to endorse the publication of this manuscript in its present form. Below some minor comments (small textual suggestions that need not be implemented at all).

Minor comments:
Line 9 Maybe change "Bacterial behavior - " to "In this model Bacterial behavior - " Just to make clear that this is not a general statement about bacterial behavior
Line 88 -91 now "They" in line 90 refers to "Cells with larger deletions" perhaps this alternative might be slightly clearer, but honestly this is a very minor detail: "Cells with larger deletions produce more antibiotic, but also produce significantly fewer spores. This effectively ensures the elimination of antibiotic-hyperproducing cells during each replicative cycle. These cells are instead repeatedly re-generated independently ... "
Line 99-102 Due to the examples here "some regions of the chromosome show higher genetic variation than others" is a bit far away from "In Streptomyces, these regions" Perhaps this could be changed to "In Streptomyces, these variable chromosomal regions are located ..."
Line 244 perhaps consider changing ", each of Ts =2500 timesteps." To "..., with each growth cycle consisting of Ts = 2500 time steps"
Line 510 consider replacing "used as" with "considered"
Line 510-511 consider replacing "These are also expected to ..." with "The production of these compounds is also expected to ..."

Yours sincerely,

Timo van Eldijk

Reviewer #3:

First, I must say that I am not familiar with the biological model (Streptomyces coelicolor). My area of expertise is modeling and computational biology. Hence, I will not be able to fully judge the significance of the results regarding the biology of Streptomyces.

This paper proposes an elegant model inspired from Streptomyces coelicolor. In this model the evolution of genome architecture enables the emergence of division of labor through large-scale chromosomal deletions occurring at specific fragile site. These deletions generate specific cell types - non-replicating antibiotic producers - while non-mutant cells replicate and benefit from the protection of their mutant siblings.

The paper is a revised version submitted after having been previously reviewed for Review Commons. In this new version the authors positively addressed all the comments raised by the reviewers and apart from minor points and comments, I see no major concern on this version. As already noted by in the previous review, the model described here is very thoroughly developed and analyzed and all major conclusions are very well supported by the results. It shows that, with only few assumptions - most of them being justified by the biology of Streptomyces - bacteria can leverage site-specific genomic instabilities to enable a stable division of labor at the colony level.

General comments:

The model is strongly inspired by Streptomyces coelicolor biology. However, it is unclear to me whether the results of the model enlighten the biology of this bacterium or whether it is "simply" a proof of concept that mutation-driven division of labor can evolve (note that I think the paper is interesting in both cases but I'd like the discussion to be more clear on that point). Specifically, couldn't the genome architecture that emerges in the model be compared to the position of specific genes in the real genome of S. coelicolor? Why didn't the authors perform a simple bioinformatics analysis of Streptomyces coelicolor genome? Indeed, on line 485, the authors explain that in Streptomyces, telomeric regions contain much of the accessory genes. Now, in the model, the telomeric region mainly contains growth-promoting genes. Are growth-promoting genes accessory? It is not clear to me and I'd like the authors to comment on that point as well as on the fact that here inter-species variability is more located close to the centromere (containing more antibiotic-genes) than in the telomeric region (containing more growth-promoting genes).

The authors thoroughly analyze the model sensitivity to numerical parameters (see appendices). However, there is at least an important "parameter" that they did not test. Here the model only accounts for a single telomeric arm. It seems that testing the evolutionary outcome with two arms would not be much more computationally expensive but it is not fully clear that the same model would give similar results with two telomeric arms (or that the two arms would be evolutionary conserved).

The model is very well described in the methods. However, in the main text (lines 176 and following) the way replication rate and antibiotic production is presented can be misleading as it could suggest that R and A are constants (this makes the reading to the following results sometimes difficult - e.g. caption of fig 1). Moreover, lines 176 and 177 hidden the \alpha_a parameter while this parameter is used later in the results (line 293). Please consider reorganizing this part to facilitate the understanding of the results.

Minor comments:
- Line 205 suggests that only genes (but not fragile sites) can be duplicated/deleted while methods (line 653 suggest that genes and fragile sites can be duplicated/deleted). Please clarify. Similarly, on line 654, "the gene copy" should be "the copy" for sake of clarity.
- Line 236: "a_g = 0.1" isn't it "\alpha_g = 0.1"?
- Line 269: "antibiotic-producing bacteria do not replicate autonomously" I don't understand "autonomously". They do not replicate at all.
- Line 293: "\alpha_a" is not defined here making the understanding difficult (although it is defined in the methods)
- Line 427: it is not fully clear to me what "this case" is referring to.
- Line 445: "that replicate and produce antibiotics". Shouldn't it be "that replicate and those that produce antibiotics" (at least for sake of clarity)?
- Line 446: please remove the closing bracket.
- Line 470: "mathematical model". I would rather say "computational model".
- Line 470: "multicellular development". I would rather say "colony development".
- Line 495: given the strong hypotheses of the model, I would replace "predicts" by "suggests".

- Lines 534 and 535: please correct the references to remove the first name ("Jean-Nicolas" in the first reference, "J." in the second).
- Line 554: missing coma after the reference (or missing "or").
- Please homogenize/correct references (missing first names in several references; some journal titles are abbreviated while most are not; ref (Hogeweg, 2012) is incomplete; ref (Macadangdang et al. 2022) is incomplete; line 938, "Jeam" should be "Jean"; line 942, "Jean-nicolas" should be "Jean-Nicolas" and "Jean-marc" should be "Jean-Marc" ...)

Minor comments on appendices:
- In figure S3, there are few (small) colonies that seem to be maintained while they don't contain any antibiotics-producing individuals. How could it be possible?
- Line 127: given the values of \beta_g tested and the results, I don't this you can say that the results are really "robust to changes in this parameter". This is not a big issue but please consider rephrasing.
- Figure S5. The green-blue colors are difficult to distinguish.
- Figure S5, S6 and S7 captions: "(> 1000 generations)" shouldn't it be "(> 1000 growth cycles)"?
- Lines 146 to 154 are rather obscure. What is K? What is \tilde{g}. Please rephrase/clarify.
- Figure S8. Why did you specifically measure two data-points here? More generally, in the sensitivity analyses, why did you change the experimental conditions for the different parameters?
- Line 186: please remove "clonally"
- Figure S10. The bottom part of the figure is difficult to read and not really informative. You could simplify the figure (as you did for fig S11 and S12 for instance).
- Figure S10 caption: "so that fragile site deletions growth genes in block". Shouldn't it be "so that fragile site deletions delete growth genes in block"?
- Section 13 (lines 281 and following). The measure is too indirect to be fully convincing on the selective nature of diversity of the antibiotic repertoire. Why don't you measure the number of mutations in antibiotic genes? Comparing mutation fixation rate with spontaneous mutation rate could better demonstrate that diversity is positively selected.
- Figure S16. Why did you change the format compared to figures S14 and S15? Keeping the same format would help comparing/understanding the figures.
- Figure S18. Question of curiosity. When division of labor evolves, the number of antibiotic genes seems rather constant whatever the volume of the antibiotic space. Why? Do you have any idea?
- Appendix 17. A remark. The computation of R involves a product of the parameter \beta_r and a quadratic sum. Hence, it is logical that R be highly robust to variations of \beta_r as the product is likely to be dominated by the quadratic sum.
- Caption of figure S23: I don't understand the last sentence of the caption.
- Line 392: "value" should be "values"
- Line 402: the sentence is misleading. Please consider rephrasing (e.g. as in fig S25 caption).

Point by point response to reviewers for the manuscript MSB-2022-11353RR, Evolution of genome fragility enables microbial division of labor, by Colizzi et al.

Reviewer #1:

I am very sorry for a delay.

As I mentioned in my first review, this is a very impressive and thorough study and it became even better after the revisions. So I would recommend publishing it, yet I think the presented arguments would become even more convincing if the Authors consider commenting on the following two subjects:

Reading the explanations that were provided to my early comment about setting up the model is such a way that the evolution of genome architecture becomes inevitable, I still think that this should be stated more clearly: With the appropriate parameters, there is a selection pressure towards a division of labour in some form. Yet the only path to develop such a division available by the model setting is via the evolution of genome architecture. It is not stated (and I think it is definitely beyond the scope of this already complicated study) whether various other mechanisms to develop such a division, be it regulatory or other heritable modifications, could be more competitive in implementing the division of labour. Yet it would be good to be able to see such a disclaimer in the Discussion.

We now make this more explicit in the Discussion - Line 435-440

Given the multiply mentioned thoroughness of the manuscript, it would be excessive to ask for a full analysis, yet a qualitative estimate of an adaptive dynamics selection gradients towards the modification of the genome architecture, or at least mentioning them, would add to more intuitive interpretation of the findings.

We now expand Appendix Section 1, to outline the evolutionary trajectory of genome structure in the population, from initial conditions (no fragile sites and homogeneously distributed growth and antibiotic genes) to the evolutionary steady state (see also Fig 3), mentioning the selection pressures that shape the genome towards enabling division of labor. Line 229-233 and Appendix 1, Fig S3.

Lines 200-201 "Resistance decreases when the difference between the two strings increases." Could it be shortly explained here or at least mentioned that the details are in Methods

We added that details are in the methods section, Line 196

Lines 431-432, "Only for an unrealistically small antibiotic deposition radius does division of labor not evolve." Word order?

We modified this to: “Division of labor does not evolve when the antibiotic deposition radius is unrealistically small”, Line 364-365

Reviewer #2:

The manuscript in its present form is a very pleasant read and it very intuitive and easy to follow, really well done to the authors. My previous comments (see previous review) have been more than adequately addressed. The authors have especially made the link between the model and the empirical system much stronger. It is now really clear how the model assumptions come about. Furthermore, the authors have also expanded on the potential general applicability of their findings. All in all, I am more than happy to endorse the publication of this manuscript in its present form. Below some minor comments (small textual suggestions that need not be implemented at all).

Minor comments:
Line 9 Maybe change "Bacterial behavior - " to "In this model Bacterial behavior - " Just to make clear that this is not a general statement about bacterial behavior

We modified the abstract, Line 26

Line 88 -91 now "They" in line 90 refers to "Cells with larger deletions" perhaps this alternative might be slightly clearer, but honestly this is a very minor detail: "Cells with larger deletions produce more antibiotic, but also produce significantly fewer spores. This effectively ensures the elimination of antibiotic-hyperproducing cells during each replicative cycle. These cells are instead repeatedly re-generated independently ... "

We modified the text as: "These antibiotic-hyperproducing cells are instead repeatedly re-generated independently in each colony following spore germination."
Line 90-91

Line 99-102 Due to the examples here "some regions of the chromosome show higher genetic variation than others" is a bit far away from "In Streptomyces, these regions" Perhaps this could be changed to "In Streptomyces, these variable chromosomal regions are located ..."

Changed, Line 101

Line 244 perhaps consider changing ", each of Ts =2500 timesteps." To "..., with each growth cycle consisting of Ts = 2500 time steps"

Changed, Line 226

Line 510 consider replacing "used as" with "considered"

Changed, Line 433

Line 510-511 consider replacing "These are also expected to ..." with "The production of these compounds is also expected to ..."

changed , Line 433-434

Yours sincerely,

Timo van Eldijk

Reviewer #3:

First, I must say that I am not familiar with the biological model (Streptomyces coelicolor). My area of expertise is modeling and computational biology. Hence, I will not be able to fully judge the significance of the results regarding the biology of Streptomyces.

This paper proposes an elegant model inspired from Streptomyces coelicolor. In this model the evolution of genome architecture enables the emergence of division of labor through large-scale chromosomal deletions occurring at specific fragile site. These deletions generate specific cell types - non-replicating antibiotic producers - while non-mutant cells replicate and benefit from the protection of their mutant siblings.

The paper is a revised version submitted after having been previously reviewed for Review Commons. In this new version the authors positively addressed all the comments raised by the reviewers and apart from minor points and comments, I see no major concern on this version. As already noted by in the previous review, the model described here is very thoroughly developed and analyzed and all major conclusions are very well supported by the results. It shows that, with only few assumptions - most of them being justified by the biology of Streptomyces - bacteria can leverage site-specific genomic instabilities to enable a stable division of labor at the colony level.

General comments:

The model is strongly inspired by Streptomyces coelicolor biology. However, it is unclear to me whether the results of the model enlighten the biology of this bacterium or whether it is "simply" a proof of concept that mutation-driven division of labor can evolve (note that I think the paper is interesting in both cases but I'd like the discussion to be more clear on that point). Specifically, couldn't the genome architecture that emerges in the model be compared to the position of specific genes in the real genome of S. coelicolor? Why didn't the authors perform a simple bioinformatics analysis of Streptomyces coelicolor genome? Indeed, on line 485, the authors explain that in Streptomyces, telomeric regions contain much of the accessory genes. Now, in the model, the telomeric region mainly contains growth-promoting genes. Are growth-promoting genes accessory? It is not clear to me and I'd like the authors to comment on that point as well as on the fact that here inter-species variability is more located close to the centromere (containing more antibiotic-genes) than in the telomeric region (containing more growth-promoting genes).

Regarding the significance of the results: the paper explains a particular aspect of the biology of Streptomyces, by providing a hypothesis for the evolution of the observed mutation-driven division of labor. In addition, other biological systems are also known to develop by DNA elimination. The model serves as a proof of concept of this kind of mutation-based development (as opposed to gene expression-based development).

We now mention this in the Discussion, see Line 405-406 and 507-508.

Regarding the proposed bioinformatic analysis: assessing gene locations in Streptomyces genomes would require that genes can be neatly classified as core or accessory. The kind of growth-promoting genes as used in the model are not necessarily "core" genes, and may fall in a broad category of genes like transcription factors and stress-response (anti-)factors, or other gene types that bias cellular processes towards rapid growth and division.
However, we did test whether genome structure would be maintained if "core" genes - such as homeostatic genes - would evolve to localize in the mutationally quiet part of the chromosome (Line 321-329, Appendix 10). Encouragingly, these genes evolved to be located to the left of the chromosome - i.e. they have little variability. This suggests that a finer genomic model with multiple gene types might also evolve a genomic architecture similar to that presented here.

The authors thoroughly analyze the model sensitivity to numerical parameters (see appendices). However, there is at least an important "parameter" that they did not test. Here the model only accounts for a single telomeric arm. It seems that testing the evolutionary outcome with two arms would not be much more computationally expensive but it is not fully clear that the same model would give similar results with two telomeric arms (or that the two arms would be evolutionary conserved).

We agree with the reviewer that including both chromosomal arms would be an interesting addition to the model, which would increase its realism and its complexity. The two arms of the Streptomyces chromosome often recombine with each other (Tidjani et al. 2020), and this recombination is an important driver of chromosome variability. For instance, telomere recombination can result in their deletion and chromosome circularisation.
We do not know whether both arms would remain stable if we introduced them and their interactions in the model, and what their evolutionary dynamics might be. This is certainly an exciting future venue for extending the model, but is outside the current scope of the manuscript due to the need to introduce more and different types of mutations. We now mention this in the Discussion Line 465-467.

The model is very well described in the methods. However, in the main text (lines 176 and following) the way replication rate and antibiotic production is presented can be misleading as it could suggest that R and A are constants (this makes the reading to the following results sometimes difficult - e.g. caption of fig 1). Moreover, lines 176 and 177 hidden the \alpha_a parameter while this parameter is used later in the results (line 293). Please consider reorganizing this part to facilitate the understanding of the results.

We now state that R and A are functions, and explicitly include \alpha_a in the description. L172-177

Minor comments:
- Line 205 suggests that only genes (but not fragile sites) can be duplicated/deleted while methods (line 653 suggest that genes and fragile sites can be duplicated/deleted). Please clarify.

Duplications and deletions affect both genes and fragile sites, we now clarify this. Line 199-200.

Similarly, on line 654, "the gene copy" should be "the copy" for sake of clarity.

Corrected, L. 561

- Line 236: "a_g = 0.1" isn't it "\alpha_g = 0.1"?
Yes, thanks for catching this. Corrected. See Fig. 1, caption.

- Line 269: "antibiotic-producing bacteria do not replicate autonomously" I don't understand "autonomously". They do not replicate at all.
We agree, we removed the word autonomously. L254

- Line 293: "\alpha_a" is not defined here making the understanding difficult (although it is defined in the methods)

We now introduce the parameter in the main text model explanation. Line 172-177

- Line 427: it is not fully clear to me what "this case" is referring to.

We removed "in this case", we feel the sentence is clearer without it. L.359

- Line 445: "that replicate and produce antibiotics". Shouldn't it be "that replicate and those that produce antibiotics" (at least for sake of clarity)?
Yes, corrected. L 377-379

- Line 446: please remove the closing bracket.
Removed. L 379

- Line 470: "mathematical model". I would rather say "computational model".
Changed (L394 and throughout the text)

- Line 470: "multicellular development". I would rather say "colony development".
Changed. L 394

- Line 495: given the strong hypotheses of the model, I would replace "predicts" by "suggests". L 415

- Lines 534 and 535: please correct the references to remove the first name ("Jean-Nicolas" in the first reference, "J." in the second).
Hopefully fixed. The issue appears when we convert the original document from Latex to docx. We will stick to Latex.

- Line 554: missing coma after the reference (or missing "or").
Corrected

- Please homogenize/correct references (missing first names in several references; some journal titles are abbreviated while most are not; ref (Hogeweg, 2012) is incomplete; ref (Macadangdang et al. 2022) is incomplete; line 938, "Jeam" should be "Jean"; line 942, "Jean-nicolas" should be "Jean-Nicolas" and "Jean-marc" should be "Jean-Marc" ...)
Fixed

Minor comments on appendices:
- In figure S3, there are few (small) colonies that seem to be maintained while they don't contain any antibiotics-producing individuals. How could it be possible?

It is likely that those colonies are not dividing labor: they have a smaller number of antibiotic genes (indicated by a lighter shade of blue), and they seem to grow slower (their final colony size is smaller than the others) so they likely have fewer growth genes which do not completely inhibit antibiotic production. These alternative strategies can arise occasionally through mutations and survive for a while because colonies only compete once their antibiotic haloes are in contact, but go extinct within a few growth cycles. We now comment on this in Appendix 2.

- Line 127: given the values of \beta_g tested and the results, I don't this you can say that the results are really "robust to changes in this parameter". This is not a big issue but please consider rephrasing.

We rephrased this sentence to "i.e. once there is a perceivable trade-off between antibiotic production and growth". L 191-193

- Figure S5. The green-blue colors are difficult to distinguish.

We increased the color contrast and the size of the bars, hopefully it is clearer now.

- Figure S5, S6 and S7 captions: "(> 1000 generations)" shouldn't it be "(> 1000 growth cycles)"?
Yes, fixed.

- Lines 146 to 154 are rather obscure. What is K? What is \tilde{g}. Please rephrase/clarify.
In the section, we aim to better characterize the model by studying which combinations of parameters affect its behavior (as opposed to individual parameters). Specifically, we focus on the trade-off between replication and antibiotic production. We find that the product of h_g and \beta_g sets the trade-off strength, so we set K = h_g \beta_g.

K is thus a measure of trade-off strength: for K= 10, division of labor evolves regardless of the individual values of h_g and \beta_g. We now explain this better in the text (Appendix 5).

\tilde{g} = g / h_g, but we see that it is confusing because we also defined \tilde{g} = g \beta_g. These are rescaled values of g. We now use two different symbols for them.

We now include this discussion in the section.

- Figure S8. Why did you specifically measure two data-points here? More generally, in the sensitivity analyses, why did you change the experimental conditions for the different parameters?
We measured two data points (1 and 2) separated by a large number of growth cycles for each of the three simulations to show that within each simulation division of labor is maintained over evolutionary time.
We now mention this in the caption of Figure S9.

The experimental conditions - i.e. all the other parameters and the initial conditions - are the same across different sections (except for the parameters under analysis in each section). In some cases we ran a smaller number of simulations, or ran simulations for a shorter time, because simulations may take very long to reach evolutionary steady state (in some cases more than one month).

- Line 186: please remove "clonally"
We changed it to: "colonies begin their growth cycle from single spores". L 256-257

- Figure S10. The bottom part of the figure is difficult to read and not really informative. You could simplify the figure (as you did for fig S11 and S12 for instance).

While we agree that the heatmap is somewhat difficult to read, we think it is important to show that the evolved architecture is consistent across the population, despite large variation in genome size.
We now mention this explicitly in the text. L 287-289

- Figure S10 caption: "so that fragile site deletions growth genes in block". Shouldn't it be "so that fragile site deletions delete growth genes in block"?
Yes, thanks. It is now Fig S11.

- Section 13 (lines 281 and following). The measure is too indirect to be fully convincing on the selective nature of diversity of the antibiotic repertoire. Why don't you measure the number of mutations in antibiotic genes? Comparing mutation fixation rate with spontaneous mutation rate could better demonstrate that diversity is positively selected.

We agree that the simulation in Section S13 does not conclusively show that selection for diversity shapes the antibiotic repertoire. It only confirms that the large and diverse antibiotic repertoire is not a consequence of the model assumption that more antibiotic genes increase production rate.
We now write this explicitly in the Section, see first paragraph of Appendix 13.

However, Fig. S18 shows that bacteria are highly susceptible to the many antibiotics produced by other colonies, and the growth cycle snapshots and videos show that invasion dynamics are driven by antibiotic competition. This, altogether, shows that having multiple antibiotics is beneficial to the colonies.
We now include this summary in Appendix Section S14, L 370-377

Measuring the fixation rate of mutations in antibiotics in our model is not trivial. For instance, some antibiotic genes can persist in the telomeres while remaining unexpressed. These

genes would drift for a while and later be duplicated into the part of the genome that antibiotic producing mutants express (the telomeres may act as a "diversity reservoir"). This would be difficult to control for. Moreover, we do not feel that our simplified model of antibiotic evolution deserves too much in-depth analysis: a more detailed model of antibiotic biosynthesis pathways would justify studying the precise impacts of drift and selection on the evolution of diversity. Nevertheless, our simplified model does reproduce the evolution of large AB diversity (as in the genus Streptomyces), and we know that this diversity is functional because most secreted antibiotics in the model are able to kill other bacteria (Fig. S18), but there are several limitations, which we report in the Discussion.

- Figure S16. Why did you change the format compared to figures S14 and S15? Keeping the same format would help comparing/understanding the figures.

We made the format of (now) Fig. S17 the same as the previous two figures.

- Figure S18. Question of curiosity. When division of labor evolves, the number of antibiotic genes seems rather constant whatever the volume of the antibiotic space. Why? Do you have any idea?

We do not know, but we hypothesize that when the volume of antibiotic space is sufficiently large, other factors contribute to the number of antibiotic genes, e.g. mutation rates, and the number of colonies that are affected by the antibiotics (the latter scales with the size and dimensionality of the lattice). These remain constant in these different simulations.

- Appendix 17. A remark. The computation of R involves a product of the parameter β_r and a quadratic sum. Hence, it is logical that R be highly robust to variations of β_r as the product is likely to be dominated by the quadratic sum.

The reviewer is correct, this is also why we test such a large range of values for this parameter. We now include this comment in the supplementary section text. L 441-443

- Caption of figure S23: I don't understand the last sentence of the caption.

Yes, it is a mistake, we only collected data from one time point.

- Line 392: "value" should be "values"

Yes, thanks!

- Line 402: the sentence is misleading. Please consider rephrasing (e.g. as in fig S25 caption).
We changed the sentence to:
"Division of labor persists in evolved genomes, when de-novo fragile site formation μ_n is set to zero. We initialized six simulations, each with one of three evolved genomes (two simulations with each initial genome)." L 482-483.

2nd Revision - Editorial Decision 19th Jan 2023

Thank you again for sending us your revised manuscript. We are now satisfied with the modifications made and I am pleased to inform you that your paper has been accepted for publication.

EMBO Press Author Checklist

Corresponding Author Name: Enrico Sandro, Colizzi	
Journal Submitted to: Molecular Systems Biology	
Manuscript Number: MSB-2022-11353	

USEFUL LINKS FOR COMPLETING THIS FORM
The EMBO Journal - Author Guidelines
EMBO Reports - Author Guidelines
Molecular Systems Biology - Author Guidelines
EMBO Molecular Medicine - Author Guidelines

Reporting Checklist for Life Science Articles (updated January
This checklist is adapted from Materials Design Analysis Reporting (MDAR) Checklist for Authors. MDAR establishes a minimum set of requirements in transparent reporting in the life sciences (see Statement of Task: 10.31222/osf.io/9sm4x). Please follow the journal's guidelines in preparing your
Please note that a copy of this checklist will be published alongside your article.

Abridged guidelines for figures
1. Data
The data shown in figures should satisfy the following conditions:
- the data were obtained and processed according to the field's best practice and are presented to reflect the results of the experiments in an accurate and unbiased manner.
- ideally, figure panels should include only measurements that are directly comparable to each other and obtained with the same assay.
- plots include clearly labeled error bars for independent experiments and sample sizes. Unless justified, error bars should not be shown for technical
- if n<5, the individual data points from each experiment should be plotted. Any statistical test employed should be justified.
- Source Data should be included to report the data underlying figures according to the guidelines set out in the authorship guidelines on Data

2. Captions
Each figure caption should contain the following information, for each panel where they are relevant:
- a specification of the experimental system investigated (eg cell line, species name).
- the assay(s) and method(s) used to carry out the reported observations and measurements.
- an explicit mention of the biological and chemical entity(ies) that are being measured.
- an explicit mention of the biological and chemical entity(ies) that are altered/varied/perturbed in a controlled manner.
- the exact sample size (n) for each experimental group/condition, given as a number, not a range;
- a description of the sample collection allowing the reader to understand whether the samples represent technical or biological replicates (including how many animals, litters, cultures, etc.).
- a statement of how many times the experiment shown was independently replicated in the laboratory.
- definitions of statistical methods and measures:
  - common tests, such as t-test (please specify whether paired vs. unpaired), simple χ2 tests, Wilcoxon and Mann-Whitney tests, can be unambiguously identified by name only, but more complex techniques should be described in the methods section;
  - are tests one-sided or two-sided?
  - are there adjustments for multiple comparisons?
  - exact statistical test results, e.g., P values = x but not P values < x;
  - definition of 'center values' as median or average;
  - definition of error bars as s.d. or s.e.m.

Please complete ALL of the questions below.
Select "Not Applicable" only when the requested information is not relevant for your study.

Materials

Newly Created Materials	Information included in the manuscript?	In which section is the information available? (Reagents and Tools Table, Materials and Methods, Figures, Data Availability Section)
New materials and reagents need to be available; do any restrictions apply?	Not Applicable	

Antibodies	Information included in the manuscript?	In which section is the information available? (Reagents and Tools Table, Materials and Methods, Figures, Data Availability Section)
For **antibodies** provide the following information:<br>- Commercial antibodies: RRID (if possible) or supplier name, catalogue number and or/clone number<br>- Non-commercial: RRID or citation	Not Applicable	

DNA and RNA sequences	Information included in the manuscript?	In which section is the information available? (Reagents and Tools Table, Materials and Methods, Figures, Data Availability Section)
**Short novel DNA or RNA including primers, probes**: provide the sequences.	Not Applicable	

Cell materials	Information included in the manuscript?	In which section is the information available? (Reagents and Tools Table, Materials and Methods, Figures, Data Availability Section)
**Cell lines:** Provide species information, strain. Provide accession number in repository **OR** supplier name, catalog number, clone number, and/**OR** RRID.	Not Applicable	
**Primary cultures:** Provide species, strain, sex of origin, genetic modification status.	Not Applicable	
Report if the cell lines were recently **authenticated** (e.g., by STR profiling) and tested for mycoplasma contamination.	Not Applicable	

Experimental animals	Information included in the manuscript?	In which section is the information available? (Reagents and Tools Table, Materials and Methods, Figures, Data Availability Section)
**Laboratory animals or Model organisms:** Provide species, strain, sex, age, genetic modification status. Provide accession number in repository **OR** supplier name, catalog number, clone number, **OR** RRID.	Not Applicable	
**Animal observed in or captured from the field:** Provide species, sex, and age where possible**.**	Not Applicable	
Please detail **housing and husbandry conditions**.	Not Applicable	

Plants and microbes	Information included in the manuscript?	In which section is the information available? (Reagents and Tools Table, Materials and Methods, Figures, Data Availability Section)
**Plants:** provide species and strain, ecotype and cultivar where relevant, unique accession number if available, and source (including location for collected wild specimens).	Not Applicable	
**Microbes:** provide species and strain, unique accession number if available, and source.	Not Applicable	

Human research participants	Information included in the manuscript?	In which section is the information available? (Reagents and Tools Table, Materials and Methods, Figures, Data Availability Section)
If collected and within the bounds of privacy constraints report on age, sex and gender or ethnicity for all study participants.	Not Applicable	

Core facilities	Information included in the manuscript?	In which section is the information available? (Reagents and Tools Table, Materials and Methods, Figures, Data Availability Section)
If your work benefited from core facilities, was their service mentioned in the acknowledgments section?	Not Applicable	

Design

Study protocol	Information included in the manuscript?	In which section is the information available? (Reagents and Tools Table, Materials and Methods, Figures, Data Availability Section)

If study protocol has been **pre-registered, provide DOI in the manuscript**. For clinical trials, provide the trial registration number **OR** cite DOI.	Not Applicable	
Report the **clinical trial registration number** (at ClinicalTrials.gov or equivalent), where applicable.	Not Applicable	

Laboratory protocol	Information included in the manuscript?	In which section is the information available? (Reagents and Tools Table, Materials and Methods, Figures, Data Availability Section)
Provide DOI OR other citation details if **external detailed step-by-step protocols** are available.	Not Applicable	

Experimental study design and statistics	Information included in the manuscript?	In which section is the information available? (Reagents and Tools Table, Materials and Methods, Figures, Data Availability Section)
Include a statement about **sample size** estimate even if no statistical methods were used.	Not Applicable	
Were any steps taken to minimize the effects of subjective bias when allocating animals/samples to treatment (e.g. **randomization procedure**)? If yes, have they been described?	Not Applicable	
Include a statement about **blinding** even if no blinding was done.	Not Applicable	
Describe **inclusion/exclusion criteria** if samples or animals were excluded from the analysis. Were the criteria pre-established?<br><br>If sample or data points were omitted from analysis, report if this was due to attrition or intentional exclusion and provide justification.	Not Applicable	
For every figure, are **statistical tests** justified as appropriate? Do the data meet the assumptions of the tests (e.g., normal distribution)? Describe any methods used to assess it. Is there an estimate of variation within each group of data? Is the variance similar between the groups that are being statistically compared?	Not Applicable	

Sample definition and in-laboratory replication	Information included in the manuscript?	In which section is the information available? (Reagents and Tools Table, Materials and Methods, Figures, Data Availability Section)
In the figure legends: state number of times the experiment was **replicated** in laboratory.	Not Applicable	
In the figure legends: define whether data describe **technical or biological replicates**.	Not Applicable	

Ethics

Ethics	Information included in the manuscript?	In which section is the information available? (Reagents and Tools Table, Materials and Methods, Figures, Data Availability Section)
Studies involving **human participants**: State details of **authority granting ethics approval** (IRB or equivalent committee(s), provide reference number for approval.	Not Applicable	
Studies involving **human participants**: Include a statement confirming that **informed consent** was obtained from all subjects and that the experiments conformed to the principles set out in the WMA Declaration of Helsinki and the Department of Health and Human Services Belmont Report.	Not Applicable	
Studies involving **human participants:** For publication of **patient photos**, include a statement confirming that consent to publish was obtained.	Not Applicable	
Studies involving experimental **animals**: State details of **authority granting ethics approva**l (IRB or equivalent committee(s), provide reference number for approval. Include a statement of compliance with ethical regulations.	Not Applicable	
Studies involving **specimen and field samples:** State if relevant **permits** obtained, provide details of authority approving study; if none were required, explain why.	Not Applicable	

Dual Use Research of Concern (DURC)	Information included in the manuscript?	In which section is the information available? (Reagents and Tools Table, Materials and Methods, Figures, Data Availability Section)
Could your study fall under dual use research restrictions? Please check biosecurity documents and list of select agents and toxins (CDC): https://www.selectagents.gov/sat/list.htm	Not Applicable	
If you used a select agent, is the security level of the lab appropriate and reported in the manuscript?	Not Applicable	
If a study is subject to dual use research of concern regulations, is the name of the **authority granting approval and reference number** for the regulatory approval provided in the manuscript?	Not Applicable	

Reporting

The MDAR framework recommends adoption of discipline-specific guidelines, established and endorsed through community initiatives. Journals have their own policy about requiring specific guidelines and recommendations to complement MDAR.

Adherence to community standards	Information included in the manuscript?	In which section is the information available? (Reagents and Tools Table, Materials and Methods, Figures, Data Availability Section)
State if relevant guidelines or checklists (e.g., **ICMJE, MIBBI, ARRIVE, PRISMA**) have been followed or provided.	Not Applicable	
For **tumor marker prognostic studies,** we recommend that you follow the **REMARK** reporting guidelines (see link list at top right). See author guidelines, under 'Reporting Guidelines'. Please confirm you have followed these guidelines.	Not Applicable	
For **phase II and III randomized controlled trials**, please refer to the **CONSORT** flow diagram (see link list at top right) and submit the CONSORT checklist (see link list at top right) with your submission. See author guidelines, under 'Reporting Guidelines'. Please confirm you have submitted this list.	Not Applicable	

Data Availability

Data availability	Information included in the manuscript?	In which section is the information available? (Reagents and Tools Table, Materials and Methods, Figures, Data Availability Section)
Have **primary datasets** been deposited according to the journal's guidelines (see 'Data Deposition' section) and the respective accession numbers provided in the Data Availability Section?	Not Applicable	
Were **human clinical and genomic datasets** deposited in a public access-controlled repository in accordance to ethical obligations to the patients and to the applicable consent agreement?	Not Applicable	
Are **computational models** that are central and integral to a study available without restrictions in a machine-readable form? Were the relevant accession numbers or links provided?	Yes	Data Availability
If publicly available data were reused, provide the respective **data citations in the reference list**.	Not Applicable	
